# Integrated analysis of metabolome, lipidome, and gut microbiome reveals the immunomodulation of *Astragali radix* in healthy human subjects

**DOI:** 10.1186/s13020-024-01045-2

**Published:** 2024-12-19

**Authors:** Wan-Yu Gui, Jun-Gang Yin, Jian-Cheng Liao, Hui-Zhi Luo, Qing You, Jia-Hui Gong, Jie Xiang, Jian-Dong Zou, Chang-Yin Li

**Affiliations:** 1 Department of Clinical Pharmacology, Jiangsu Province Hospital of Chinese Medicine, Affiliated Hospital of Nanjing University of Chinese Medicine, No. 155 Hanzhong Road, Nanjing, 210029 China; 2 Center of Good Clinical Practice, Jiangsu Province Hospital of Chinese Medicine, Affiliated Hospital of Nanjing University of Chinese Medicine, No. 155 Hanzhong Road, Nanjing, 210029 China

**Keywords:** *Astragali radix*, Immunomodulation, Lipidomics, Metabolomics, Gut microbiota

## Abstract

**Background:**

As a typical medicinal food homology species, Chinese herbal medicine *Astragali radix* (AR) has been widely used to regulate the human immune system worldwide. However, the human immunomodulation of AR and its corresponding mechanisms remain unclear.

**Methods:**

First, following a fortnight successive AR administration, the changes in immune cytokines and immune cells from 20 healthy human subjects were used as immune indicators to characterize the immunomodulatory effects of AR. Subsequently, ultra-high-performance liquid chromatography coupled with quadrupole-time-of-flight mass spectrometry (UHPLC-Q-TOF/MS) based lipidomics and metabolomics analysis was performed on human serum, urine, and feces samples to investigate the changes in metabolic profiles. Then, 16S rRNA gene sequencing of feces samples was adopted for the changes of human gut microbiota. Finally, correlation analysis was conducted on the gut microbiome, metabolome/lipidome data, and immune indicators.

**Results:**

AR displayed good safety in clinical use and posed a minor impact on gut microbiota major genera, global metabolic profiles, and immune cells. Meanwhile, AR could significantly up-regulate anti-inflammatory cytokines, down-regulate serum creatinine and pro-inflammatory cytokines, promote the anabolism of arginine, glycerolipid, sphingolipid, and purine, and the catabolism of phenylalanine and glycerophospholipid. Moreover, these AR-induced changes were closely correlated with significantly decreased *Granulicatella*, slightly higher *Bifidobacterium*, *Ruminococcus*, and *Subdoligranulum*, and slightly lower *Blautia*.

**Conclusion:**

The study clearly demonstrated that AR could modulate the human immune, by modifying the metabolism of amino acids, lipids, and purines in a microbiota-related way.

*Trial registration* ChiCTR, ChiCTR2100054765. Registered 26 December 2021-Prospectively registered, https://www.chictr.org.cn/historyversionpub.html?regno=ChiCTR2100054765

**Supplementary Information:**

The online version contains supplementary material available at 10.1186/s13020-024-01045-2.

## Introduction

Over the last few decades, with the gradual enhancement of public health consciousness, there has been a significant shift in the interest of consumers toward Medicinal Food Homology (MFH) species, which could not only satisfy hunger but also display several beneficial health properties, including the promotion of immune function and the prevention and management of specific diseases [1, 2]. Currently, up to 93 species of Chinese medicines have been identified as MFH by the Ministry of Health of China. Of them, *Astragali radix* (AR) has been commonly considered a promising MFH to regulate immunity, owing to its well-known temperate immunomodulation effect.

AR is the dried root of *Astragalus membranaceus* (Fisch.) Bge. var. *mongholicus* (Bge.) Hsiao or A. *membranaceus* (Fisch.) Bge. According to the theory of Traditional Chinese Medicine (TCM), AR could strengthen Qi (the vital substance essential for the body’s structure and life-sustaining activities) and to enhance Yang (the fundamental energy that powers metabolism and physiological functions), and it is widely understood that these functions are closely linked to the immunomodulation of humans. AR was once declared as the premier tonic among herbal treatments and is now acknowledged as a vital tonic for enhancing energy levels and modulating the immune system. As a promising MFH herb, AR has been widely accepted across the world. For example, early in the USA Dietary Supplement Health and Education Act of 1994, AR was already categorized as a legal dietary supplement. Consequently, AR tea has been approved as over-the-counter (OTC) health products in the U.S. dietary supplement market [3]. Given its famous immunomodulation and worldwide popularity, it is necessary to fully demonstrate the AR’s effect on the immune system and explore the underlying mechanisms through modern pharmacological studies.

Several researches have been attempted to investigate the immunomodulation effects of AR. A metabolomic study revealed that 30-day consecutive administration of AR could significantly alter glycerophospholipid, pyrimidine, and sphingolipid metabolisms in mice plasma, thereby regulating the immune function in healthy mice in a dose-dependent way [4]. Astragalus polysaccharide (APS) was demonstrated to be a topical mucosal adjuvant to enhance the growth-inhibitory effects of immune checkpoint inhibitors on pulmonary metastatic melanoma in mice [5]. In Oreochromis niloticus, dietary AR nanoparticles were proven to have a synergistic influence on growth performance, disease resistance, and immunological and antioxidant responses [6]. These results could provide some scientific evidence to support the immunomodulation benefits of AR at the animal or cellular levels. However, owing to the large difference between animal/cell and human, clinical studies are required to fill the gap and validate the results mentioned above, to further confirm the immune improvement of AR, and to explore the corresponding mechanisms. Moreover, AR consists of various types of constituents, including saponins, flavonoids, polysaccharides, and amino acids, which as a whole collectively contributed to its immunomodulation effects. Accordingly, holistic research approaches such as metabolomics (polar metabolites) and lipidomics (non-polar metabolites) analysis should be considered for the pharmacological research on AR.

Just like most MFHs, AR is usually orally administrated, however, previous studies have revealed that all three main types of constituents in AR, polysaccharides, saponins, and flavonoids are not easily absorbed into the blood. Accordingly, these primary constituents of AR are inevitably degraded by gut microbiota, thereby improving their digestive efficiency and also providing a consistent nutrient source for the microorganisms. Actually, growing evidence has indicated a strong interaction between MFH and gut microbiota, which can significantly influence human health and the progression of diseases [2]. The strong connection between gut microbiome and immunity has gained much more attention and become a hot issue. Consequently, we hypothesized that gut microbiota may serve as a possible target of AR to exhibit its comprehensive immunomodulatory function. In addition, the gut microbiota could release large numbers of metabolites such as short-chain fatty acids (SCFAs), choline, vitamins, and indole derivatives, playing extensive effects on various host organs. Therefore, the impact of AR on the gut microbiota may lead to alterations in the metabolome, contributing to the immunomodulation of humans.

**Fig. 1:**
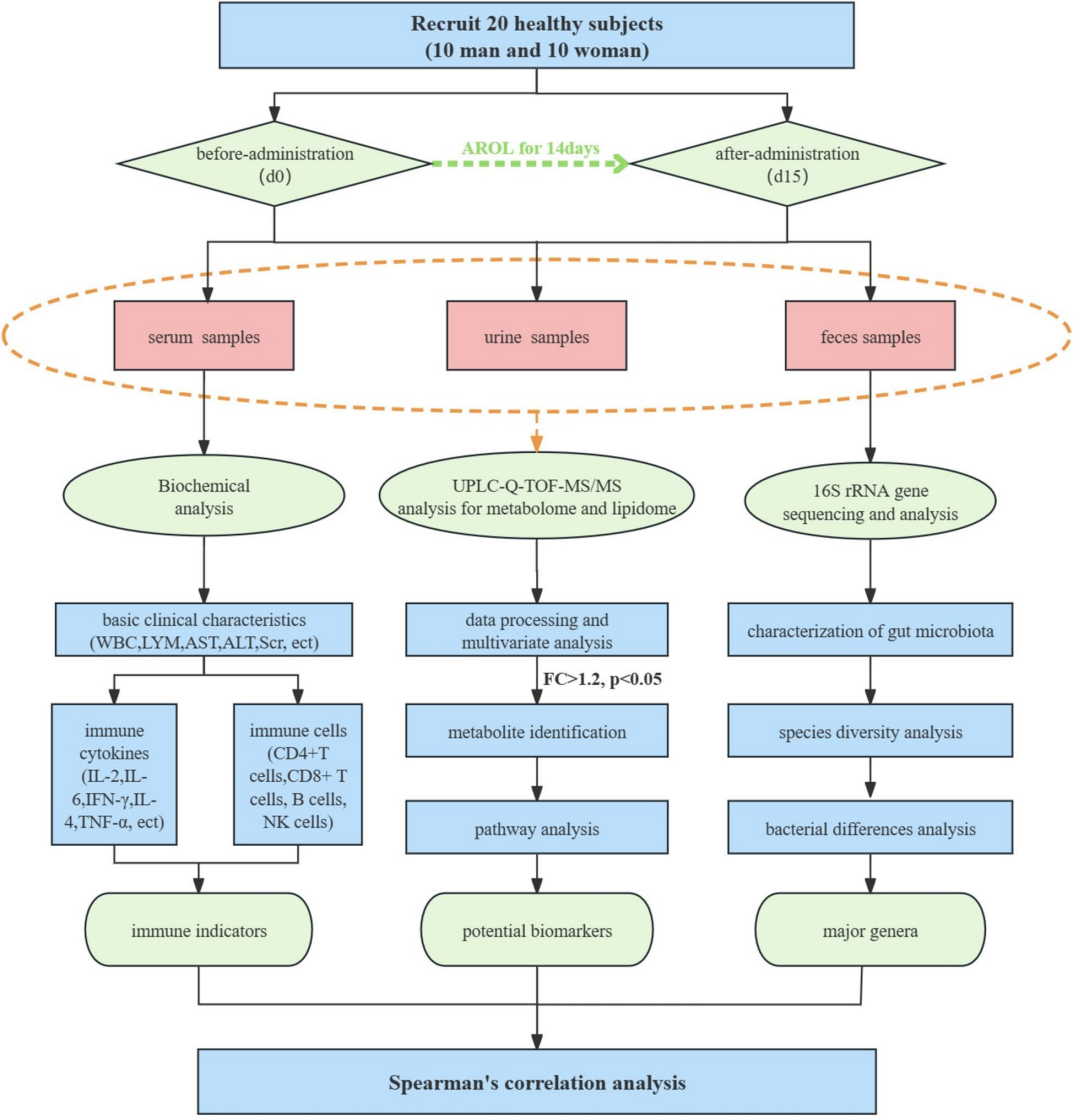
The schematic diagram of the present study

This study aimed to investigate the immunomodulatory function of AR in healthy human subjects and explore its underlying mechanisms by the integrated analysis of metabolome, lipidome, and gut microbiome. Twenty healthy subjects were recruited to be orally administrated AR for 14 consecutive days, to assess the modulatory effect of AR on human immunity by comparing the changes between before and after administration in biochemical indicators, endogenous metabolites, and gut microbiota. First, physiological and biochemical analysis was used for safety assessment and basic population characterization, while immune cells and cytokines were examined as immune indicators (IIs) to assess the immunomodulatory effects of AR in humans. Then, untargeted metabolomics analysis (MA) and lipidomics analysis (LA) of serum, urine, and feces were performed based on the ultra-high-performance liquid chromatography coupled with quadrupole-time-of-flight mass spectrometry (UHPLC-Q-TOF/MS) analytical platform, to screen and identify the potential biomarkers (PBs) and related metabolic pathways induced by AR administration. Next, 16S rRNA gene sequencing analysis was employed to identify the AR-induced significant changes in the gut microbiome and dominant genera. Finally, correlation analysis was performed among the IIs, dominant genera, and PBs to demonstrate the possible microbiota-associated metabolic mechanisms for AR immunomodulation. The schematic diagram of this study is presented in Fig. 1. To our best knowledge, this study is the first time to examine the metabolic effects of AR in humans, and the results would provide novel insights into the interplay between metabolism and gut microbiota in AR’s immunomodulatory mechanisms, thereby establishing a foundation for the future development of AR-related dietary supplements.

## Materials and methods

### Reagents and materials

Formic acid of LC/MS grade (Lot 214911, with a purity of 98%) was procured from Fisher Scientific (Waltham, MA, USA). Acetonitrile, methanol, and isopropanol of HPLC grade were obtained from Merck in the United States. Ammonium formate (Lot H3520, purity ≥ 99%) was acquired from Honeywell (Fluka, USA). Calibration solutions for the AB SCIEX Triple TOF™ system, specifically APCI positive (Lot A9311) and APCI negative (Lot M106480), were sourced from AB Sciex Pte. Ltd., located in the USA.

Astragali radix oral liquid (AROL, Lot 210046, Specification: 10 mL per bottle), as the most common oral preparation of AR provided by the Jiangsu Provincial Hospital of Chinese Medicine. The extraction process of AROL was as follows: boiling 1 kg of AR with 8 L of water for 2 h, followed by a second boiling with 6 L of water for an additional 1.5 h. The solutions obtained from the water extraction process were first filtered and then combined. Subsequently, they were concentrated using evaporation under reduced pressure, resulting in an extract with a relative density of 1.25 (50–60 °C). Then, the concentrated extract was precipitated using 75% ethanol and refrigerated for 24 h. Next, the supernatant was obtained and the ethanol was recovered and removed. After recovery of ethanol, water was incorporated into the extract solutions until the total volume achieved 700 mL and then was refrigerated again for another 24 h. The extract solutions were filtered again, and 265 mL of sucrose solution was added, then followed by purified water, until the total volume reached 1000 mL. After being well stirred, filled, and sanitized by steam at 100 °C for 30 min, AROL was finally obtained with a dosage of 1 g/mL.

Human 12-item cytokine assay kit (Lot 210913) was obtained from Qingdao Raisecare Biotechnology Co., Ltd., RAISECARE, China. Antibodie 6-Color TBNK (Lot 36990) for flow cytometry detection was purchased from BD, USA.

### Identification of the chemical constitution of AROL

The 50 μL of AROL mixed with 450 μL of methanol–water (1:1, *v/v*). Prior to use, the mixture was sonicated for 30 min and then centrifuged at 12,000*g* for 10 min at 4 °C. The prepared sample was used for UHPLC-Q-TOF/MS analysis, and the specific conditions are listed in Supplementary Information S1. The data obtained from UHPLC-Q-TOF/MS were then imported into PeakView 2.2 (AB SCIEX) for compound screening and identification. According to the AR components identified from AR preparations in our previous studies [7–9] and literatures [10]. The self-built chemical library of AR, including retention time, the mass-to-charge ratio of precursor and fragment ions, was constructed to facilitate the identification of components in AROL. By detailed comparison with the above library with PeakView software, the components in AROL were efficiently screened and identified, some of them could be further confirmed by comparing with the corresponding authentic standards if they are available. The detailed information of all 23 available AR chemical standards in our lab, including CAS number, source, and Lot number are summarized in Table S1.

### Subjects and study design

Twenty healthy subjects (10 men and 10 women) were recruited from Jiangsu Provincial Hospital of Chinese Medicine. The inclusion criteria were: (1) physically healthy with no history of heart, liver, kidney, gastrointestinal, neurological, or metabolic abnormalities; (2) age ranged from 18 to 40 years; (3) the calculation of body mass index (BMI) ranged from 19.0 to 26.0; (4) exhibiting a standard physical examination and typical laboratory results during the screening visit. Exclusion criteria were as follows: (1) biochemical tests show abnormalities such as blood count, liver function, renal function, etc.; (2) taking any other medication in the 2 weeks before the trial; (3) menstruating, pregnant, and lactating women. This research complied with the principles of the Helsinki Declaration and was approved by the ethics committee of the Chinese Clinical Trial Registry (no. ChiCTR2100054765). Before the trial initiation, each participant received detailed information about the research, and written informed consent was obtained from all individuals involved.

Participants were asked to complete the intervention by drinking one bottle (10 mL/day) of AROL after supper for 14 consecutive days (the daily dose was 10 g per adult). Fasting blood, urine, and feces were collected in the morning from the subjects before (Group d0) and after (Group d15) AROL administration. The blood samples were generally divided into two sections: one was immediately performed for blood routine examination and immune cell detection, and the other was allowed to stand at room temperature for 30 min then centrifuged at 1300*g* for 15 min at 15 °C to yield the corresponding serum aliquots. The serum aliquots were ultimately stored at − 80 °C for subsequent analysis of biochemical parameters as well as LA/MA. Urine and feces samples were collected half an hour after blood sampling and then were homogenized, aliquoted, and preserved at − 80 °C for further LA/MA and (or) gut microbiome analysis.

### Assessment of clinical pharmacological effect

The pharmacological effects of AROL administration on humans were investigated from the following three aspects: physiological functions, biochemical functions, and IIs. Physiological functions of the subjects were mainly characterized by gender, age, BMI, diastolic blood pressure (DBP), and systolic blood pressure (SBP), as well as blood routine parameters including white blood cell count (WBC), red blood cell count (RBC), platelet (PLT), neutrophil ratio (NEUT), hemoglobin (HGB), and lymphocyte (LYM). Liver function was assessed by the serum levels of aspartate aminotransferase (AST) and alanine aminotransferase (ALT), while renal function was indicated by the levels of blood urea nitrogen (BUN) and serum creatinine (Scr). 

Both serum immune cytokines and blood immune cells were analyzed as IIs. Briefly, the serum levels of eight immune cytokines (IFN-γ, TNF-α, IL-2, IL-4, IL-6, IL-10, IL-17, and IL-12P70) were determined using the human 12-item cytokine assay kit (Qingdao Raisecare Biotechnology Co., Ltd., RAISECARE, China), while the condition of all three types of immune cells including Thymus dependent lymphocytes (T cells), Bursa dependent lymphocytes (B cells), and Natural Killer (NK) cells were described by flow cytometer analysis. The detailed analysis procedures could be found in Supplementary Information S2.

### Metabolomics and lipidomics analysis

A comprehensive analysis of three biological sample types (serum, urine, and feces), was conducted to thoroughly characterize the metabolic changes induced by successive AROL administration. It is widely recognized that serum and feces are rich in both polar metabolites and lipids, while urine primarily comprises a significant quantity of polar metabolites. Therefore, in the current study, serum and feces samples were subjected to both LA and MA using a combination of Reverse-Phase Liquid Chromatography-Q-TOF/MS (RPLC-Q-TOF/MS) and Hydrophilic Interaction Liquid Chromatography-Q-TOF/MS (HILIC-Q-TOF/MS) techniques, while urine samples were merely analyzed to MA by HILIC-Q-TOF/MS.

#### Sample preparation

Serum and feces followed similar sample preparation methods. Firstly, 60 mg of feces sample was homogenized by adding water at 1:10 to obtain the homogenate. The thawed serum samples (40 μL) and feces homogenates (500 μL) underwent extraction using 750 μL of methyl tert-butyl ether (MTBE) and 225 μL of methanol. Next, 188 μL of water was added, and the sample was centrifuged for 10 min (12,000 g, 4 °C). The supernatant (350 μL) was collected and evaporated. The resulting dried sample was then re-dissolved in a 200 μL mixture of methanol and toluene (9:1, *v/v*) for LA analysis using RPLC-Q-TOF/MS. The bottom polar phase (300 μL) was re-extracted using 900 μL of a 1:1 (*v/v*) acetonitrile and isopropanol solution, shaken for 6 min and then centrifuged. The supernatant was transferred into Eppendorf tubes and subsequently concentrated by evaporation using a Speedvac vacuum concentrator. Following this, it was re-solubilized using 100 μL of acetonitrile and water (4:1, *v/v*). After centrifugation for 5 min, the supernatant was carefully moved to an autosampler for subsequent MA using HILIC-Q-TOF/MS.

Each urine sample was first thawed and vortexed at ambient temperature. Then, 120 μL portions from each sample were treated with 240 μL of methanol for deproteinization. The resulting mixture was vortexed for 30 s and subjected to centrifugation. Finally, the supernatant was carefully collected and injected into the HILIC-Q-TOF/MS system for analysis.

For each biological sample type, combine 20 μL of supernatant from each individual sample to create the appropriate mixed quality control (QC) sample.

#### Sample analysis

Chromatographic separation was conducted using an Agilent 1290 Infinity Ultra Performance Liquid Chromatography system. Mass detection was performed with the Triple TOF™ 5600 (AB SCIEX, USA), a hybrid triple Q-TOF mass spectrometer equipped with an electrospray ionization source. To maintain mass accuracy during detection, an automated calibration system for mass spectrometry was employed, with recalibration occurring every 2 h. This procedure employed APCI calibration solutions (both positive and negative), that were specifically developed for the AB SCIEX Triple TOF™ system. The specific RPLC/HILIC-Q-TOF/MS method is presented in Supplementary Information S3. The entire process of operations and data acquisition was conducted using Analyst TF 1.6 software (AB SCIEX, United States).

Different types of biological samples were analyzed separately, meanwhile, MA and LA in different mass ion modes should also be performed in different analytical runs. Therefore, a total of 10 analytical runs should be finished in the present study. In each run, all samples were injected in a random order. To ensure optimal performance of the analytical system at the beginning of the run, the pooled QC sample underwent five repetitions of analysis prior to the examination of the actual sample. The analytical system’s stability was carefully monitored by injecting the sample every ten throughout the analytical run.

#### Data quality assessment

Using PeakView (version 1.2), data quality was initially assessed by checking the overlap of QC samples in the total ion chromatograms (TICs). To further assess the stability and reproducibility of the analytical method, the RSD% values for *m/z*, RT, and intensity associated with the aforementioned features in the QC samples were calculated. This was done after randomly selecting five typical features with varying RT and *m/z* values in both positive and negative ion modes. Furthermore, the Principal Components Analysis (PCA) score plots' clustering kinds of QC samples could be considered a useful indicator of the quality of the MA and LA data.

#### Data analysis

The raw data obtained from UHPLC-Q-TOF/MS were analyzed using MS-DAIL software (version 4.9) for peak detection, matching, normalization, and alignment and the “80% rule” was used to eliminate missing peaks [11]. Then MS-FLO [12] (https://msflo.fiehnlab.ucdavis.edu) was utilized to optimize the MS features in the data matrices generated by MS-DAIL. This approach aims to eliminate inaccuracies in peak data, enhancing the overall quality of the dataset, it facilitating the generation of concise preprocessed data matrices ultimately.

The preprocessed data matrices were subsequently transferred into SIMCA-P 14.0 (Umetrics, Umea, Sweden) for conducting multivariate statistical analyses, which encompassed PCA and the partial least-squares discriminant analysis (PLS-DA), to assess the holistic metabolic changes induced by AR. It is important to emphasize that urine data matrices need to be normalized based on the total useful MS signals (TUMS) [13] way and if necessary, systematic error removal using random forest (SERRF) (https://slfan2013.github.io/SERRF-online/) could be also used as an effective normalization tool to improve the data quality, especially when QC samples were not clustered well and changed gradually with the injection order in PCA score plots [14]. The R^2^Y values of the PLS-DA surpassed 0.5, while the Q^2^ values were above 0.4, and the discrepancy between R^2^Y and Q^2^ was less than 0.4, indicating that the PLS-DA models were acceptable and could be used to yield the variable importance in projection (VIP) values for identifying significant variables. Meanwhile, using MetaboAnalyst 5.0 [15] (https://www.metaboanalyst.ca/), the (normalized) data matrices were subjected to the two-tailed paired t-test and fold change (FC) analysis to screen the significant variables induced by AROL administration. Variables with *p* < 0.05, and FC > 1.2 (or < 0.83) were considered as significant molecular features (SMFs) between groups.

#### Metabolite identification and metabolic pathway analysis

The AR-induced SMFs were mainly assigned or identified based on matching with the Laboratory-built database in MS-DAIL and comparing their quasi-molecular ions and fragment ions in PeakView. The associated SMFs were considered as preliminary identified PBs only when both the quasi-molecular and fragment ions closely aligned with known metabolites in the self-built database, exhibiting a high level of mass accuracy within 10 mDa. The identified PBs were then named uniformly according to the information provided by the Human Metabolome Database (HMDB, https://hmdb.ca/). Metabolic pathway enrichment analysis in MetaboAnalyst 5.0 was employed to further elucidate the PBs-related metabolic pathways. Finally, the PBs data matrices containing PB name, Inchikeys, PubChem ID, SMILES, *p*-value, and FC-value were then submitted to ChemRICH [16] (http://chemrich.fiehnlab.ucdavis.edu/) analysis for chemical similarity enrichment calculations of the PBs.

### Gut microbiome analysis

#### 16S rRNA gene sequencing analysis

Microbial genomic DNA was isolated from feces samples using the E.Z.N.A.® soil DNA kit (Omega Bio-tek, Norcross, GA, USA) according to the manufacturer’s instructions. The hypervariable V3-V4 region of bacterial 16S rRNA was amplified using the primer pair: 341F (CCTAYGGGRBGCASCAG) and 806R (5′-GGACTACHVGGGTWTCTAAT-3′) in a PCR reaction conducted on an ABI GeneAmp® 9700 PCR thermocycler (Applied Biosystems, Foster, CA, USA). The PCR products were isolated from a 2% agarose gel and subsequently purified with the AxyPrep DNA Gel Extraction Kit (Axygen Biosciences, Union City, CA, USA), following the manufacturer’s protocol. The purified DNA was quantified using a Quantus™ Fluorometer from Promega, based in Madison, WI, USA. According to normal methods from Majorbio BioPharm Technology Co. Ltd. (Shanghai, China), purified amplicons were pooled in equimolar quantities and paired-end sequenced using an Illumina MiSeq PE300 platform/NovaSeq PE250 platform (Illumina, San Diego, CA, USA).

#### Data analysis

Raw paired files were analyzed using the Mothur package (version 1.30.2). The following criteria were employed to eliminate reads for sequence QC: the presence of ambiguous bases, sequence length less than 380 bp, chimeric sequences, and any contaminant sequences. Following the normalization of data, operational taxonomic units (OTUs) were identified utilizing the SILVA reference database [17], with a similarity threshold set at 97%. Species diversity study and bacterial differentiation analysis were conducted using the Majorbio Cloud Platform (www.majorbio.com). Alpha diversity indices, comprising community richness (Chao and ACE indices) and diversity (Shannon and Simpson indices), were calculated using Mothur software (version 1.30.2). At the same time, Bar plots were made to represent the composition of species and the relative abundance of each species using R (version 3.3.1). The beta diversity Non-metric multidimensional scaling (NMDS) which using bray curtis distance with significance calculated by the PERMANOVA method and PLS-DA were both analyzed in QIIME (version 1.9.1). To evaluate the importance of variation in taxonomic abundance and distribution between groups, differential abundance analysis was carried out using the Wilcoxon signed-rank test and FDR-adjusted *p*-values.

### Spearman’s correlation analysis

The Omic Studio tools (https://www.omicstudio.cn) were used to calculate the Spearman’s correlation coefficients among significantly different indicators, dominant microbiota in the genus level, and PBs from the metabolome and lipidome of serum, urine, and feces. Using the *p*-value and Spearman’s correlation coefficient (*r*) as thresholds: *p* < 0.05 was considered to be significantly correlated.

### Statistical analysis

The results were displayed as mean ± SD of physiological functions, biochemical functions, and IIs. GraphPad Prism 8 software was used for one-way analysis of variance (ANOVA) and paired t-test. A denoting significance was set for *p* < 0.05. Descriptive statistics of the *p*-value and FC-value of identified PBs in serum, urine, and feces were completed in GraphPad Prism 8.

## Results

### Component analysis of AROL

AR is the only traditional Chinese medicine contained in AROL. The UHPLC-Q-TOF/MS TICs of AROL in the positive and negative ion modes are shown in Figure S1. Totally 69 representative compounds were finally identified, and their detailed information was summarized in Table S2. Of these, 23 compounds were subsequently validated through comparison with the corresponding standards. The component analysis results clearly demonstrated that AROL was mainly composed of AR-derived flavonoids, saponins, amino acids, and polysaccharides.

### Clinical pharmacological assessment

A total of twenty normal weight (BMI: 21.7 ± 1.7 kg·m^−2^) healthy adults between 24–36 years old were recruited to the protocol. All twenty subjects completed this study with high compliance and no significant changes in their dietary patterns were observed throughout the intervention period. Their blood pressures including SBP and DBP were also measured, and the SBP/DBP in Group d0 was 116.7 ± 8.7/72.2 ± 7.5 mmHg and 118.1 ± 8.5/75.10 ± 8.0 mmHg in Group d15. No notable differences were observed in physiological functions or liver function between Group d0 and Group d15 (Table S3), which confirmed that no serious adverse events were observed in subjects after AROL administration. However, Scr in renal function showed a significant decrease in Group d15.

**Fig. 2:**
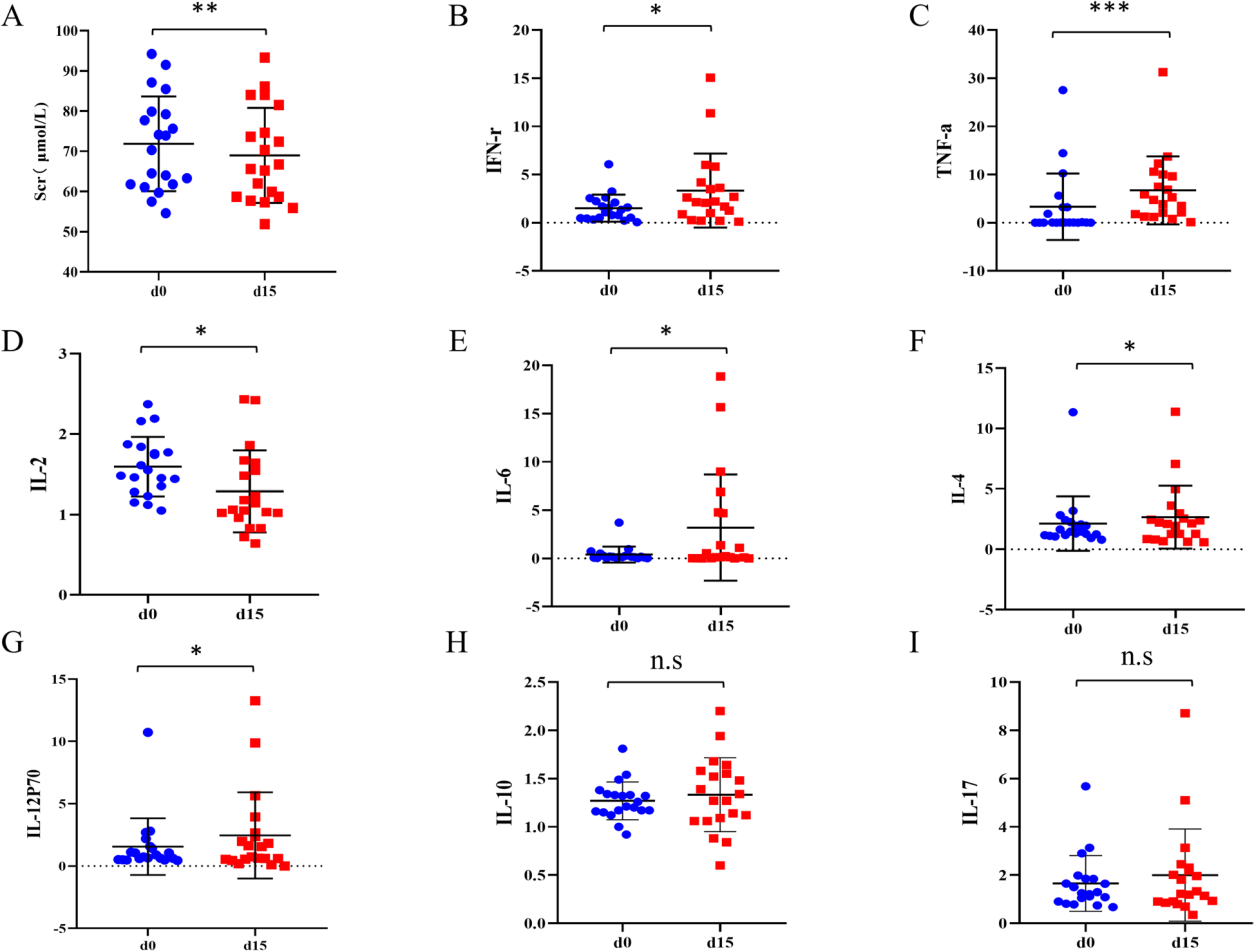
The modulation of human immunity after the administration of AROL. **A** Measurement of serum creatinine (Scr) in two groups. The impact of AROL on immune cytokines **B** TNF-α, **C** IFN-γ, **D** IL-2, **E** IL-4, **F** IL-6, **G** IL-12P70, **H** IL-10, and **I** IL-17. (*p < 0.05; **p < 0.01; ***p < 0.001, n.s means non-significant.)

Two types of IIs, cells, and cytokines, were then used to assess the modulation of human immunity after the administration of AROL. As for blood immune cells (Figure S2), there was an increase in the levels of CD4+ and CD8+ T cells, while a decline was observed in CD19+ (B cells) and CD3-CD16+CD56+ (NK cells) after AROL administration, however, these changes in immune cells showed no statistical significance. Unlike this, most of the immune cytokines were greatly affected by AROL administration. As shown in Fig. 2, five immune cytokines (IFN-γ, TNF-α, IL-4, IL-6, and IL-12P70) were significantly up-regulated in Group d15, while IL-2 was significantly down-regulated. IL-10 and IL-17 also showed a decreased trend but no significant change.

### Metabolomics and lipidomics analysis

#### Data quality assessment

**Fig. 3:**
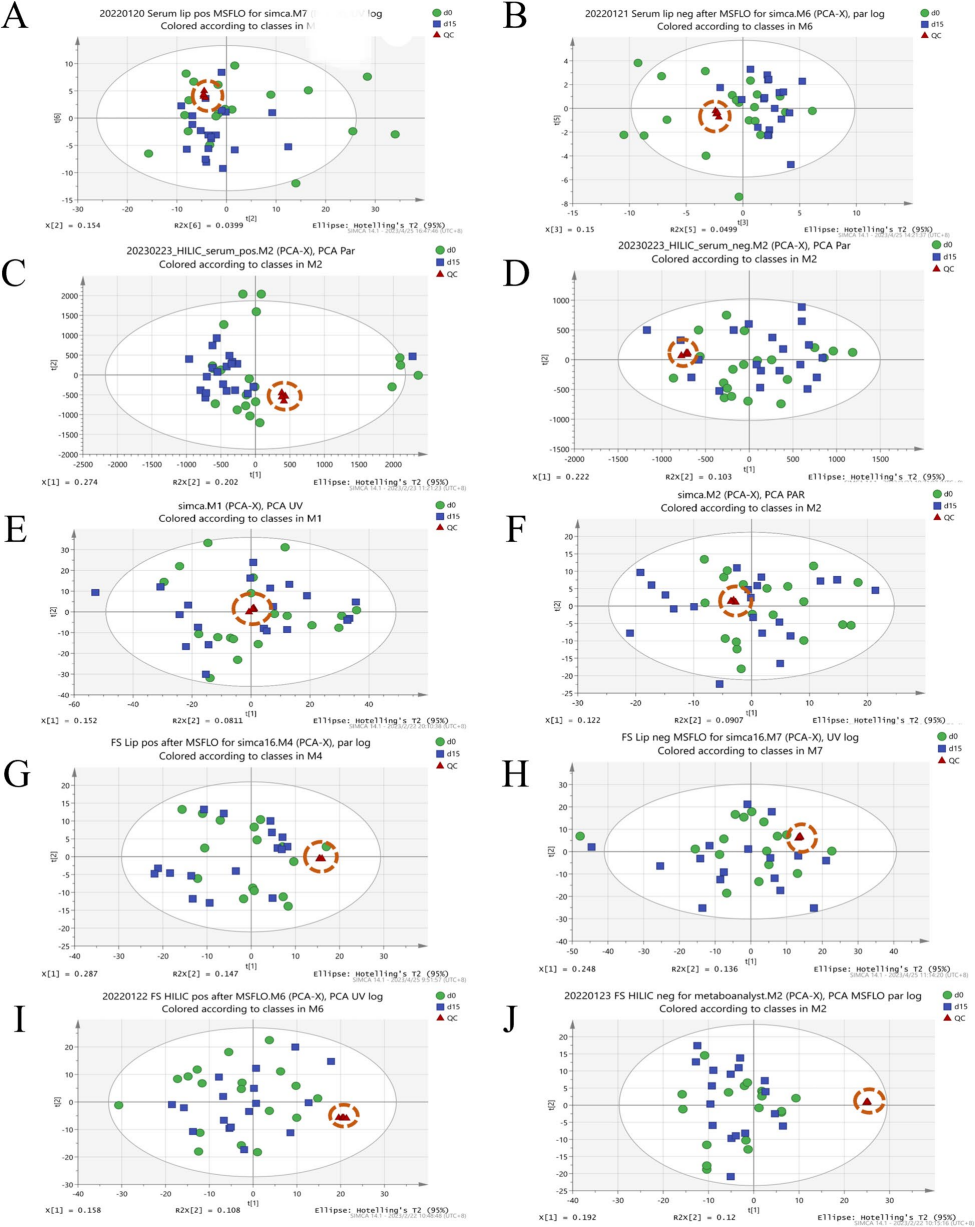
The relatively subtle impact of AROL administration on the metabolic profiles of healthy adults. PCA score plots (after SERRF) of Group d0 and Group d15: **A** Serum LA pos; **B** Serum LA neg; **C** Serum MA pos; **D** Serum MA neg; **E** Urine pos; **F** Urine neg; **G** Feces LA pos; **H** Feces LA neg; **I** Feces MA pos; **J** Feces MA neg. *LA* Lipidomics analysis, *MA* Metabolomics analysis, *pos* positive mode, *neg*: negative mode

As shown in Figure S3, the TICs of all the pooled QC samples displayed excellent overlap throughout each analytical run. This result indicated the instrumental system maintained high consistency during sample analysis, ensuring the collected metabolomics and lipidomics datasets from serum, urine, and feces were of superior quality. Meanwhile, throughout each analysis, the RSD% values for the *m/z* and RT of five characteristic features remained below 0.001% and 0.7%, respectively, and the RSD% values for intensity were consistently under 30% (Table S4), further indicating that the instrumental stability and repeatability were acceptable [18]. It should be noted that the cluster of QC samples in our original data was not always good enough, just as shown in Figure S4, suggesting that there may be some systematic error in these ten runs. SERRF is a normalization method based on QC samples, which could eliminate unnecessary system changes in large sample sets [14]. After being SERRF-normalized, the QC clustering was significantly improved (Fig. 3), clearly demonstrating the suitability and potential of SERRF normalization, so the SERRF-normalized data was adopted of all ten runs for further analysis in our study. Collectively, the raw data collected by our current RPLC/HILIC-Q-TOF/MS methods was demonstrated to be reliable and could reflect the real metabolic condition of the corresponding biological samples.

#### Metabolic profiling analysis

As an unsupervised analytical approach, PCA offers an impartial overview of the metabolic profiles for all samples. Visual inspection of PCA score plots in both LA and MA (Fig. 3) showed no obvious difference between Group d0 and Group d15, indicating AROL administration has a relatively subtle impact on the metabolic profiles of healthy adults. This could partially establish a solid foundation for considering AROL as a nutritional supplement. PLS-DA was subsequently employed to further investigate the variations in metabolic profiles between the groups. The PLS-DA score plots (Figure S5) show that the subjects could be divided into two distinct clusters corresponding to Group d0 and Group d15, demonstrating that successive AROL administration could produce some metabolic changes. However, not all the PLS-DA models could meet the given acceptance criteria in “Data analysis” section, and especially Q^2^Y values in the PLS-DA models from urine and feces were all less than 0.25, indicating there may be large and uncontrollable individual differences in the clinical samples. Accordingly, VIP values from PLS-DA were not used for the following feature screening. Instead, the two-tailed paired t-test and FC analysis were adopted to filter out the AR-induced SMFs (Figure S6). Table S5 summarizes the changes in the number of features during the entire data processing of LA and MA. In serum and feces samples, the number of features was high, and the identified PBs including different lipid and polar compounds by performing LA and MA respectively, which confirmed the necessity of our analysis using a combination of RPLC-Q-TOF/MS and HILIC-Q-TOF/MS techniques.

#### Serum lipidomics and metabolomics analysis

1166 and 1752 features were obtained from serum LA and MA respectively following MS-DIAL data preprocessing and MS-FLO data cleaning, from which a total of 209 and 82 SMFs were screened out according to the criteria of *p* < 0.05, and FC > 1.2 (or < 0.83). 51 and 38 differential metabolites were finally identified as AR-induced serum PBs, and their detailed information including* t*_R_, *m/z*, ion type, formula, name, PubChem CID, HMDB ID as well as *p* and FC values from group comparison, were shown in Table S6.

**Fig. 4:**
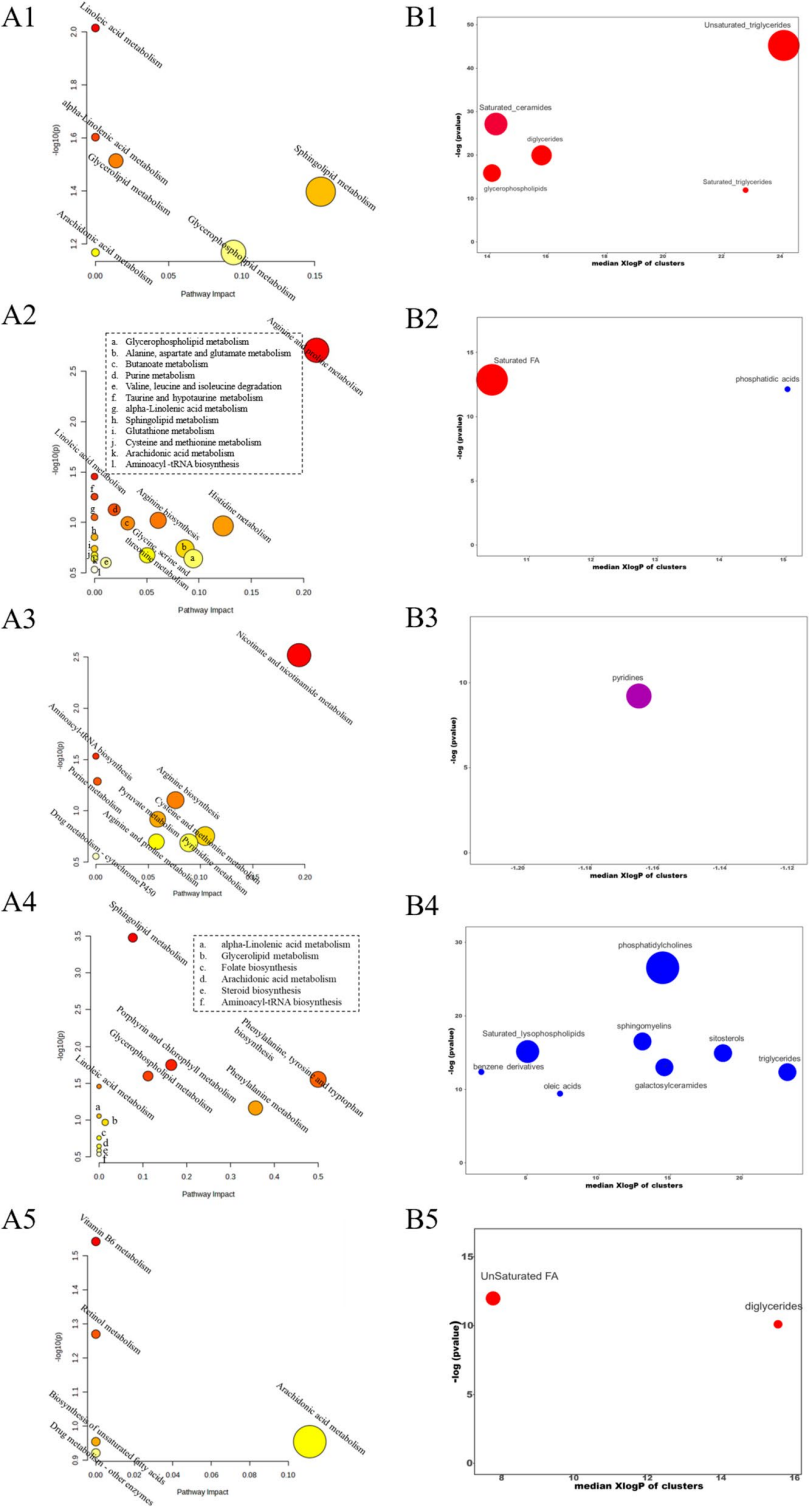
Identification and pathway analysis of serum, urine, and feces metabolites. **A** The pathway enrichment analysis of AROL-induced PBs. **B** Chemical similarity enrichment analysis of the PBs. Cluster colors give the proportion of increased or decreased compounds (red = increased, blue = decreased, purple = both increases and decreases) in each cluster. Dots size indicates the number of PBs in each cluster. The top of the Y-axis shows the most obvious clusters. (1) serum, LA; (2) serum, MA; (3) urine; (4) feces, LA; (5) feces, MA. *LA* Lipidomics analysis, *MA* Metabolomics analysis

Metabolic pathway enrichment analysis showed that only 11 (out of 51) LA-derived serum PBs could be associated with valid KEGG IDs. As a result, six pathways were enriched and each of these pathways was linked to lipids (Fig. 4A1). The major enriched pathways (*p* < 0.05) were linoleic acid metabolism, alpha-linolenic acid metabolism, glycerolipid metabolism, and sphingolipid metabolism. These pathways were enriched by three classes of metabolites, phosphatidylcholines (PC), TG, and sphinganine, which were all significantly up-regulated in the serum of Group d15. Up to 17 (out of 38) MA-derived serum PBs were found to be enriched for valid KEGG IDs, and consequently as many as 17 metabolic pathways were enriched (Fig. 4A2). Of them, the major pathways (*p* < 0.05) were arginine and proline metabolism and linoleic acid metabolism, with increased aminobutyric acid, L-proline, ornithine, and phosphatidylcholine in Group d15.

Since only 11 (out of 51) LA-derived serum PBs could be enriched with effective KEGG IDs, the metabolite enrichment analysis with ChemRICH was further performed. Data matrices of serum PBs were then subjected to ChemRICH analysis for the determination of chemical similarity enrichment of the PBs. Five clusters including triglycerides (TG), glycerophospholipids, diglycerides (DG), and ceramides (Cer) were enriched in the ChemRICH analysis of 51 serum PBs from LA (Fig. 4B1), and these clusters were significantly up-regulated in Group d15. As for 38 serum PBs from MA, only two clusters were enriched: Saturated Fatty Acids (SFAs) with an up-regulated tendency, and phosphatidic acids with a significant down-regulation in Group d15 (Fig. 4B2).

#### Urine metabolomics analysis

A total of 1752 features were detected in the MA, with 113 of them were screened out as SMFs. Finally, 41 differential metabolites were identified as AR-induced urine PBs, and their detailed information was summarized in Table S7. 26 of 41 identified urine PBs were found to be related to 9 metabolic pathways by pathway enrichment analysis (Fig. 4A3). Of them, the major pathways (*p* < 0.05) were aminoacyl-tRNA biosynthesis and nicotinate and nicotinamide metabolism, with an increase in l-arginine and a decrease in nicotinamide, *N*1-methyl-2-pyridone-5-carboxamide and l-methionine in Group d15. Moreover, three of the compounds were enriched to the cluster of pyridines (Fig. 4B3) through ChemRICH analysis, with a significant decrease in nicotinamide and *N*1-Methyl-2-pyridone-5-carboxamide and a significant increase in nitrendipine in Group d15.

#### Feces lipidomics and metabolomics analysis

After preliminary data processing by MS-DAIL and MS-FLO, 1968 and 2059 features were present in feces LA and MA, respectively, from which 198 and 82 SMFs were screened. Of them, 64 and 40 metabolites were finally identified as AR-induced feces PBs, and their detailed information was shown in Table S8. Significantly, only 27 out of the 64 feces PBs were enriched with valid KEGG IDs in LA, resulting in the enrichment of a total of 12 metabolic pathways through pathway enrichment analysis (Fig. 4A4). Five major enriched pathways (*p* < 0.05) were sphingolipid metabolism, glycerophospholipid metabolism, phenylalanine, tyrosine, and tryptophan biosynthesis, linoleic acid metabolism, and phenylalanine metabolism. These pathways were enriched by five classes of metabolites including SM, galactosylceramide, glucosylceramide, PC, and phenylalanine which were all significantly down-regulated in the feces of Group d15. Up to 19 (out of 40) MA-derived feces PBs could be enriched for valid KEGG IDs, as well as 5 metabolic pathways were enriched (Fig. 4A5). The major enriched pathway (*p* < 0.05) was vitamin B6 metabolism, with increased 2-Oxo-3-hydroxy-4-phosphobutanoic acid in Group d15.

According to ChemRICH analysis, 64 feces PBs from LA could be roughly divided into eight clusters, including PC, sphingomyelins (SM), saturated_lysophospholipids, sitosterols, TG, galactosylceramides, benzene derivatives, and oleic acids (Fig. 4B4). These clusters were all significantly down-regulated in Group d15. As for 40 feces PBs from MA, two clusters were enriched, DG and UFA both with an up-regulated tendency in Group d15 (Fig. 4B5).

#### Comprehensive analysis of serum, urine, and feces

Up to 234 AR-induced PBs were screened and identified from LA and MA, of which 89, 41, and 104 were in serum, urine, and feces, respectively. The identification of such a large number of PBs partly indicated that AR could regulate human metabolic profiles. Further analysis (Figure S7) showed that the median FC-values of these PBs in three samples after AR administration were all less than 2, and the median *p*-values were no more than 0.025, clearly demonstrating the mild regulatory effect of AR on human metabolome and lipidome. Chemical structure enrichment analysis of PBs resulted in 18 clusters, including 7, 1, and 10 from serum, urine, and feces. A total of 49 metabolic pathways were enriched by pathway enrichment analysis using Metaboanalyst, of which 23, 9, and 17 were from serum, urine, and feces. Venn analysis among serum, urine, and feces was performed for AR-induced PBs, their related pathways, and enriched compound clusters to find the common changes in all three biological samples. As shown in Figure S8A, no shared identified PBs were observed in the three samples, reflecting the advantage of the comprehensive metabolomic analysis performed in this study. 2-Benzylsuccinic acid was present in both urine and feces. Four lipid PBs, namely SM, Pantothenol, TG, and DG, were common in serum and feces. Besides, up to 99 PBs were only detected in feces, 85 PBs were merely present in serum and 51 PBs were unique in urine, fully demonstrating the necessity of simultaneous analysis of various biological samples.

Figure S8B illustrates that there are four pathways (purine metabolism, cysteine and methionine metabolism, arginine and proline metabolism, and arginine biosynthesis) were common in both serum and urine, and six pathways (glycerolipid metabolism, alpha-Linolenic acid metabolism, arachidonic acid metabolism, sphingolipid metabolism, glycerophospholipid metabolism, and linoleic acid metabolism) were common in serum and feces. Only aminoacyl-tRNA biosynthesis was simultaneously enriched in all three samples, corresponding to three amino acid metabolites including L-phenylalanine, L-proline, and L-arginine. Therefore, phenylalanine metabolism and arginine metabolism are considered to be the predominant metabolic pathways. Since many lipids within the same class often have a shared KEGG ID, the pathway enrichment analysis feature in MetaboAnalyst was not particularly effective for annotating lipid metabolites. In contrast, ChemRICH was able to partially enhance the understanding of the structural grouping and relationships among the lipid PBs. As shown in Figure S8C, three clusters (Cer, TG, and DG) were common in serum and feces. It is evident that there are more lipid metabolites and lipid metabolism pathways shared in serum and feces, and thus lipid metabolism pathways also serve as priority pathways regulated by AR. Therefore, it could be speculated that amino acid metabolism and lipid metabolism are the main metabolic pathways by which AR regulates human immunity.

### Gut microbiome analysis

#### Characterization of gut microbiota

In this study, feces samples were used to evaluate the improvement of AROL on the human gut microbiota. The 16S rRNA sequencing yielded a total of 4,142,541 optimized sequences. After being filtered by a 97% identity threshold, a total of 1077 OTUs was obtained for further gut microbiome analysis. Both the species accumulation curves and the rarefaction curves demonstrated that the sequencing depth, data quality, and the sample size were sufficient (Figure S9).

#### Species diversity analysis

**Fig. 5:**
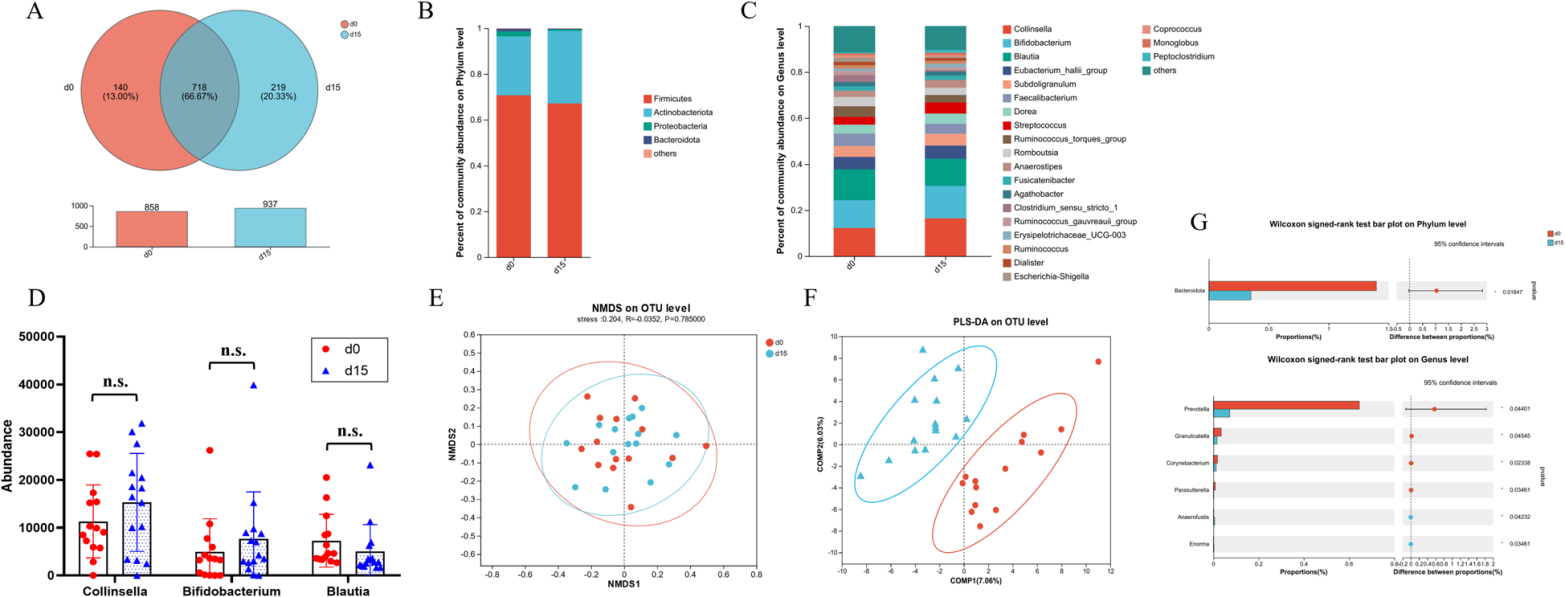
AROL regulated gut microbiota of healthy humans. **A** Shared and unique microbiota between Group d0 and Group d15. The structural composition of the gut microbiota in each group at the **B** phylum level and **C** genus-level (relative abundances are reported on the vertical axis). **D** Changes in the abundance of *Collinsella*, *Bifidobacterium*, and *Blautia* after AROL administration. **E** NMDS analysis on OTU level. **F** PLS-DA analysis on OTU level. **G** The difference in abundance at the phylum level and the genus level in Group d0 and Group d15. (*p < 0.05; **p < 0.01; ***p < 0.001)

As shown in Fig. 5A, Venn analysis revealed that Group d0 had 140 unique OTUs and Group d15 had 219 distinct OTUs, while up to 718 OTUs (accounting for 2/3 of the total OTUs) were common between the two groups, indicating that Group d15 possessed a slightly higher richness of gut microbiota than Group d0. Similarly, the alpha diversity analysis revealed no significant changes between Group d0 and Group d15, with the corresponding *p*-values of 0.810, 0.879, 0.395, and 0.348 for Chao, ACE, Shannon, and Simpson indices (Figure S10). The results revealed that the administration of AROL did not produce notable alterations in the abundance or diversity of the microbial community, indicating that AROL has a limited impact on the gut microbiome of healthy adults. To illustrate the effect of AROL administration on species composition, bar plots were employed at the phylum and genus taxonomic levels. As displayed in Fig. 5B, *Firmicutes*, *Actinobacteria*, *Proteobacteria*, and *Bacteroidota* were the dominant bacterial phylum in both groups and accounted for more than 95% of the bacteria in the microbial communities. In Group d15, *Firmicutes* showed a downward trend while *Actinobacteriota* displayed a clear upward trend. At the genus level, the fecal microbiome was dominated by *Collinsella*, *Bifidobacterium*, *Blautia* and *Eubacterium_hallii_group*, and *Subdoligranulum* (Fig. 5C). We further compared the abundance differences of the top three genera with high abundance (*Collinsella*, *Bifidobacterium*, and *Blautia*) between two groups. In Group d15, the abundance of *Collinsella* and *Bifidobacterium* exhibited an upward trend, whereas the levels of *Blautia* displayed a downward trend (Fig. 5D).

#### Bacterial differences analysis

As shown in Fig. 5E, the clustering of the two groups in the NMDS plot was nearly overlapped (*p* = 0.954), indicating the similar community composition between Group d0 and Group d15. However, the PLS-DA score plot revealed a clearer distinction between the two groups, which indicated that AROL exhibited significant modulatory effects on gut microbial community structure (Fig. 5F). Figure 5G showed that there are considerable variations in the abundance of phylum and genus between Group d0 and Group d15. Especially at the phylum level, a marked reduction in the abundance of *Bacteroidota* was observed in Group d15. At the genus level, administration of AROL resulted in significant changes in six genera. Specifically, *Anaerofustis* and *Enorma* exhibited substantial increases, whereas *Prevotella*, *Granulicatella*, *Corynebacterium*, and *Parasutterella* were notably reduced in Group d15.

### Spearman’s correlation analysis

Spearman’s correlation analysis was applied to explore the relationship among the changes in metabolic profiles, gut microbes, and significant IIs.

**Fig. 6:**
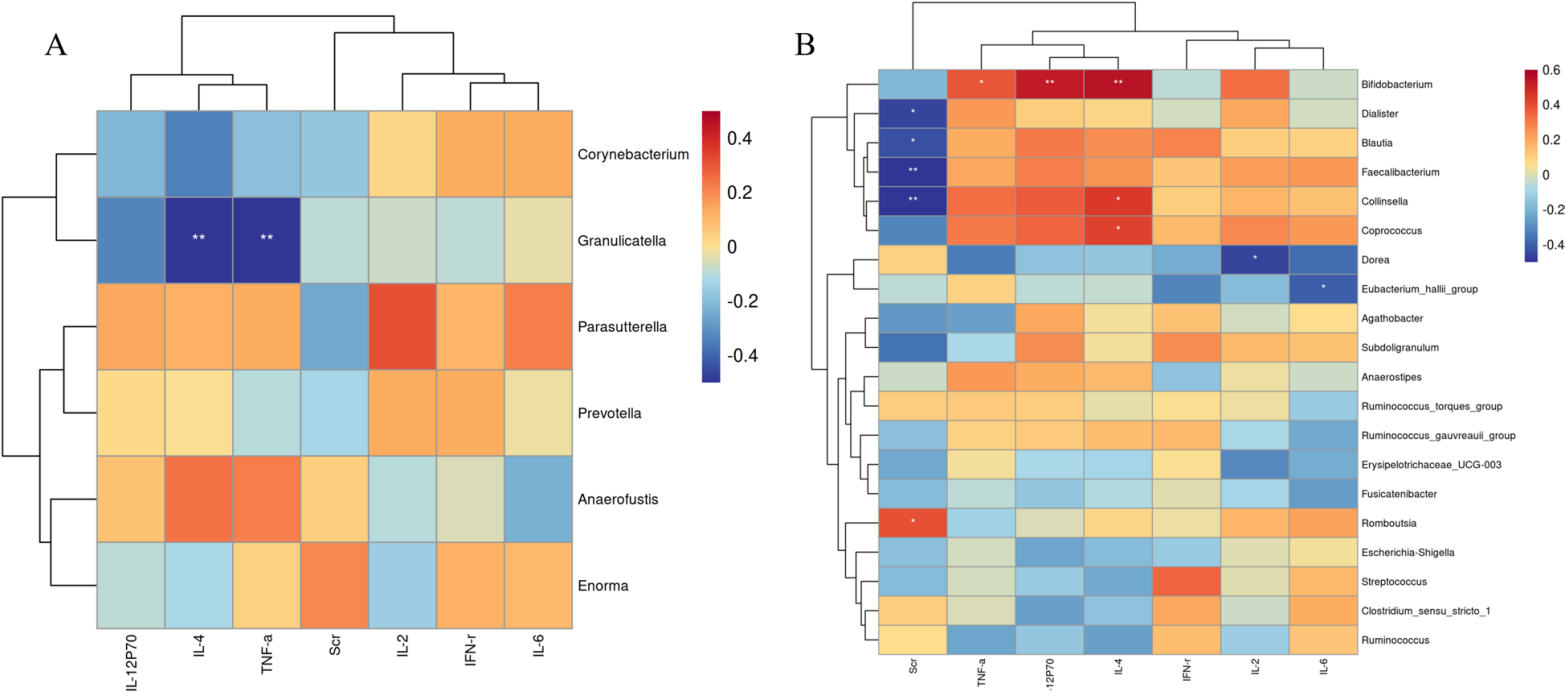
Spearman’s correlation analysis between genera and significant immune indicators (IIs). **A** Between 6 differential genera and seven significant IIs. **B** Between the 20 major genera of abundance and seven significant IIs. (*p < 0.05; **p < 0.01; ***p < 0.001)

Seven significant IIs were attempted to be correlated with six differential gut genera and twenty major abundant genera, respectively. As shown in Fig. 6A, of all the six different genera, only *Granulicatella* was negatively correlated with TNF-α and IL-4, and there was no more correlation between the remaining genera and significant IIs. Further correlation analysis between the differential genus *Granulicatella* and PBs, revealing a positive correlation with GlcCer (*p* < 0.05), SM (*p* < 0.05), and Lyso Phosphatidylethanolamine (LysoPE) (*p* < 0.05), a negative correlation with L-arginine (*p* < 0.01) and DG (*p* < 0.05). As shown in Fig. 6B, nine of twenty major genera were significantly correlated with at least one of the immune cytokines. Notably, *Bifidobacterium* was correlated with three indicators, including a positive correlation with IL-4 (*r* = 0.555, *p* = 0.002), IL-12P70 (r = 0.524, *p* = 0.004), and TNF-α (*r* = 0.381, *p* = 0.046). Additionally, Scr and five genera were significantly correlated, including a negative correlation with *Dialister* (*r* = − 0.466, *p* = 0.012), *Collinsella* (*r* = − 0.548, *p* = 0.002), *Blautia* (*r* = − 0.439, *p* = 0.019), and *Faecalibacterium* (*r* = −0.521,* p* = 0.004) and a positive correlation with *Romboutsia* (*r* = 0.393, *p* = 0.039). These findings suggested that the immunomodulation of AR may be closely associated with major genera rather than differential microbiota. In order to explore the possible microbiota-mediated metabolic mechanisms for the immunomodulation of AR, the top 20 major genera and seven significant IIs were further used to be correlated with AR-induced PBs from different biological samples.

**Fig. 7:**
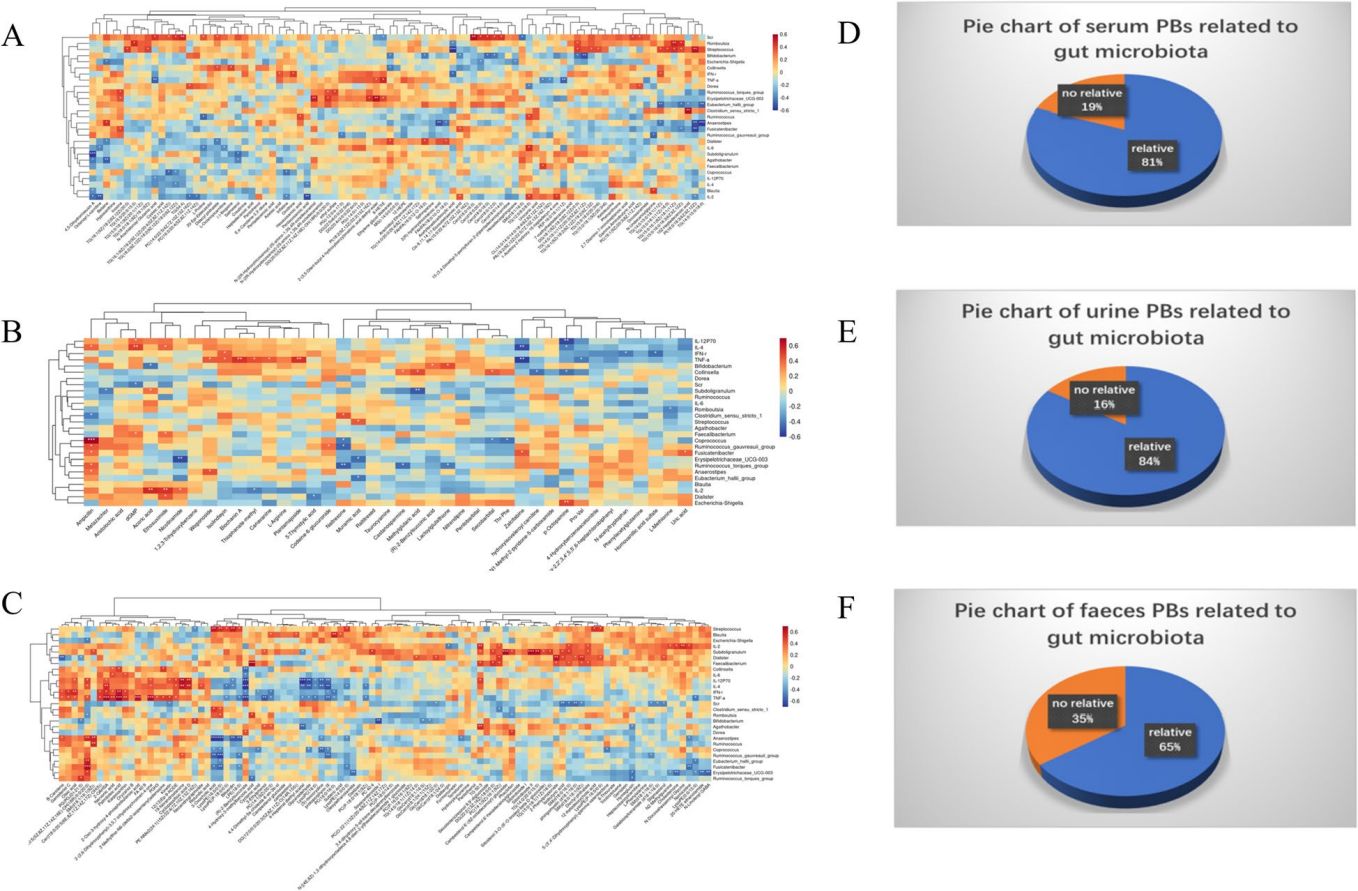
Spearman’s correlation analysis of 7 immune indicators, 20 major genera, and AR-induced PBs. Hierarchical clustering analysis between AR-induced PBs from **A** serum, **B** urine, and **C** feces and gut microbiota, as well as immune indices; red represents a positive correlation, while blue represents a negative correlation, the darker color indicates a stronger correlation (*p < 0.05; **p < 0.01; ***p < 0.001). The pie chart of AR-induced PBs from **D** serum, **E** urine, and **F** feces related to gut microbiota

AR-induced serum PBs correlation analysis demonstrated that (Fig. 7A) there were strong correlations between six major microbiota genera including *Bifidobacterium*, *Anaerostipes*, *Streptococcus*, *Clostridium*, *Erysipelotrichaceae*, and *Fusicatenibacter*, and four types of serum PBs including glycerides (eg., TG), sphingolipids (eg., Cer), FA, and carnitines. Generally, 81% of serum PBs and gut microbiota are directly correlated (Fig. 7D). Typically, TG showed a strong positive correlation with *Bifidobacterium* (*p* < 0.01) and *Streptococcus* (*p* < 0.05), while l-Proline was positively correlated with *Ruminococcus* (*r* = − 0.476, *p* = 0.010)*.* In addition, *Bifidobacterium* was also positively correlated with Insoine (*r* = 0.489, *p* = 0.006) and polyunsaturated fatty acids (PUFAs) (*p* < 0.05). There were also significant correlations between serum PBs and significant IIs, and especially, Scr showed a strong positive correlation with Cer (*p* < 0.05).

AR-induced urine PBs correlation analysis (Fig. 7B) demonstrated that there was a strong correlation between two genera including *Erysipelotrichaceae_UCG-003* and *Subdoligranulum,* and two PBs including Niacinamide and Methylglutaric acid. *Erysipelotrichaceae_UCG-003* was negatively correlated with Niacinamide (*r* = − 0.538, *p* = 0.003). Although only two urine PBs had a strong correlation with genera, up to 84% of urine PBs correlated with gut microbiota (Fig. 7E). In addition, there were also significant correlations between urine PBs and significant IIs, for example, IL-2 was positively correlated with ethosuximide (*r* = 0.535, *p* = 0.002) and acoric acid (*r* = 0.583,* p* < 0.001).

AR-induced feces PBs correlation analysis (Fig. 7C) demonstrated that there were six genera including *Bifidobacterium*, *Erysipelotrichaceae*, *Faecalibacterium*, *Ruminococcus*, *Streptococcus*, and *Subdoligranulum* correlated strongly with five types of feces PBs including SCFAs (eg., Isobutyric acid), Phenylalanine (eg., L-phenylalanine, 2-Phenylpropionate), glycerides (eg., DG, TG), glycerophospholipids (eg., LysoPE, PC), and sphingolipids (eg., SM, Cer). Overall, gut microbiota and 65% feces PBs were correlated (Fig. 7F). *Ruminococcus* and SM (*r* = 0.55, *p* = 0.005) showed a positive correlation while *Subdoligranulum* and Isobutyric acid (*r* = − 0.439, *p* = 0.033) showed a negative correlation. Notably, *Bifidobacterium* showed negative correlations with GlcCer (*p* < 0.05) and L-phenylalanine (*r* = − 0.537, *p* = 0.035). Meanwhile, Scr showed a strong negative correlation with Isobutyric acid (*r* = − 0.527, *p* = 0.008) and SM (*p* < 0.01), while TNF-α (*p* < 0.05) and IFN-γ (*p* < 0.05) showed strong positive correlations with Cer. In addition, PC showed strong negative correlations with IL-2 (*p* < 0.05), IL-4 (*p* < 0.05), and TNF-α (*p* < 0.05).

## Discussion

Considering that AR is usually orally administrated, AROL was selected as the representative preparation of AR in the present study. As shown in “Reagents and materials” section, the preparation process indicated that one bottle of AROL (10 mL) merely consists of AR water extract (corresponding to 10 mg of herbs), 2.65 mL of 85% (g/mL) sucrose solution (corresponding to 2.25 g sucrose) and purified water. Given sucrose and water were normally considered as less bioactive, we could reasonably attribute the effects of AROL to AR. Moreover, according to our previous studies, literatures, and online databases, all 68 identified compounds (except for sucrose) in AROL could be derived from AR, further demonstrating that AROL could be used to evaluate the regulation of AR on human immunity.

In this study, physiological and biochemical functions were used to describe the basic clinical characteristics of the subjects, while immune cells and immune cytokines were used to further evaluate the regulation of AR on human immunity. Unlike other basic clinical characteristics, the most common indicator of renal function Scr was significantly decreased after AROL administration, indicating the good nephroprotection potential of AR. Moreover, it has been shown that Scr could regulate CD8^+^ T cell activity through the ATP/AMPK-mediated TCR signaling pathway, thereby exerting the immunomodulatory effect [19] and accordingly, Scr may also be considered as a potential immune indicator.

As the typical IIs, all three kinds of blood immune cells (T, B, and NK) were not significantly changed by AROL administration. T cell, as the most crucial and functional immune cell in acquired immunity, has two primary subgroups CD4+ and CD8+ T cells. CD4+ T cells usually served as helper T cells, could significantly enhance and support the immune response, while CD8+ T cells could demonstrate a suppressive influence on immune responses [20]. There was an increased trend in CD4^+^ and CD8^+^ T cells in healthy volunteers after AR administration. Consistent with this, the study in Yorkshire pigs has also shown that APS enhanced the immune response by increasing the number of CD4^+^ and CD8^+^ T cells [21]. Consequently, activating CD4^+^ and CD8^+^ T cells and promoting T cell number and function may be considered as the manifestation of AR immunomodulation. Meanwhile, AR may also exert its immunomodulatory effect by decreasing the expression of B cells and NK cells.

Unlike immune cells, most immune cytokines were greatly altered by AROL, with six out of eight cytokines (IL-4, IL-6, TNF-α, IFN-γ, IL-12P70, IL-2) showing significant changes, demonstrating its better sensitivity as IIs. Among them, TNF-α, IFN-γ, and IL-2 were considered as pro-inflammatory cytokines, IL-4 and IL-12P70 were generally considered as anti-inflammatory cytokines, and IL-6 had both pro-inflammatory and anti-inflammatory effects. IL-4 is known to activate CD4+ T cells through the JAK1, JAK3, and STAT6 pathways, promoting the proliferation of both activated T and B cells [22]. IL-6 could activate B- and T-lymphocytes and trigger the acute phase response and the coagulation cascade [23]. TNF-α and IFN-γ were known as the primary mediators in inflammatory response and served an essential immunomodulatory function across a range of systemic and dermatologic conditions [24, 25]. IL-12P70 was crucial for CD8^+^ T cell immunity according to report [26]. The decrease of IL-2 could promote the growth of CD4+ T cells and the differentiation of NK cells, thereby inhibiting inflammatory response to regulate human immunity [27]. The up-regulation of anti-inflammatory cytokines (IL-4, IL-6, and IL-12P70) and the decrease of pro-inflammatory cytokine IL-2, indicated the immune enhancement effect of AR, while the increase of TNF-α and IFN-γ suggested that AR may possess complex mechanisms of immunomodulation, which required further investigation.

Recent research has revealed that immune regulation is significantly linked to changes in the intestinal environment, especially regarding the composition of gut microbiome [28]. As shown in Fig. 5G, AROL could induce significant changes of six different genera, of them, *Prevotella, Corynebacterium*, *Parasutterella*, and *Granulicatella* were decreased, while *Anaerofustis* and *Enorma* were increased. *Prevotella* is a widely present anaerobic Gram-negative bacteria belonging to Bacteroidota phylum in the healthy human gut. The increase in *Prevotella* abundance has been shown to correlate with enhanced T helper type 17 (Th17)-mediated mucosal inflammation [29]. In the present study, the decrease in *Prevotella* after drug administration indicated that AR may reduce inflammatory responses and regulate human immunity. *Corynebacterium* is classified as a nonlipophilic, nonfermentive, urease- and nitrate-positive species, which is regarded as a component of the bacterial flora found in the oropharyngeal region. Sakamoto et al. demonstrated that *Corynebacterium* induced inflammation mediated by innate lymphoid cells through a mechanism dependent on IL-7 receptors, S1P receptor 1, and CCR6, this process ultimately resulted in pyroptotic cell death [30]. As an important intestine genus in type 2 diabetes and obesity development, *Parasutterella* has been linked to inflammatory responses in the intestinal mucosa as well as systemic metabolic disturbances, which may indicate its involvement in the onset of low-grade systemic metabolic inflammation resulting from dysbiosis [31]. A recent study revealed that APS could improve immune dysfunction by downregulating *Parasutterella* abundance and regulating the related TLR4/NF-κB pathways in rats [32]. Similarly, we speculated that the decrease in the abundance of the above three inflammation-related genera *Prevotella*, *Corynebacterium*, and *Parasutterella*, may be the immunomodulation mechanism of AR. However, up to now, there have been no immunity-related publications on the other three genera (*Granulicatella*, *Anaerofustis*, and *Enorma*). Besides, among six different genera, only *Granulicatella* was negatively correlated with IL-4 and TNF-α. Further researches are still required to confirm the above findings.

Alternatively, we further explored the relationship between the major genera and the significant IIs, and the results demonstrated that *Bifidobacterium*, as the second most abundant major genus, displayed the most prominent correlation with the significant IIs in terms of both number and strength. *Bifidobacterium* was positively correlated with 3 indicators including IL-4, IL-12P70, and TNF-α. Similar findings were also shown in previous studies, *Bifidobacterium* as a well-known probiotic could enhance the proliferation and differentiation of CD4+ and CD8+ T cells, up-regulate anti-inflammatory cytokines IL-4 and IL-12P70 and down-regulate the pro-inflammatory cytokine TNF-α, and thereby promoting human immunity [33, 34]. Therefore, we speculated that AR could up-regulate *Bifidobacterium* to promote the release of cytokines such as TNF-α, IL-12P70, and IL-4 and the proliferation of CD4^+^ and CD8^+^ T cells, thereby modulating human immunity.

Previous studies have revealed that AR could modulate immunity by regulating endogenous substances and metabolic pathways in mice [4, 5]. In our study, the PCA analysis showed that AR has no dramatic effect on the human metabolic profile, suggesting the advantage of AR as an MFH. However, the PLS-DA analysis clearly showed that AR could alter some metabolites and pathways. A total of 234 metabolites were finally identified as AR-induced PBs, and accordingly, three types of main pathways induced by AR were enriched, including AA metabolisms (arginine metabolism, proline metabolism, phenylalanine metabolism), lipid metabolisms (glycerolipid metabolism, glycerophospholipid metabolism, sphingolipid metabolism, linoleic metabolism, alpha-linolenic acid metabolism), and purine nucleotide metabolisms. Furthermore, there were also strong correlations between the PBs involved in these pathways and the significant IIs. In particular, a positive correlation was observed between Scr and SM, Cer, and TG, while a negative correlation was found with Isobutyric acid. Furthermore, TNF-α and IFN-γ showed a positive association with Cer, both PC and LysoPC exhibited negative correlations with TNF-α, IL-4, and IL-2.

**Fig. 8:**
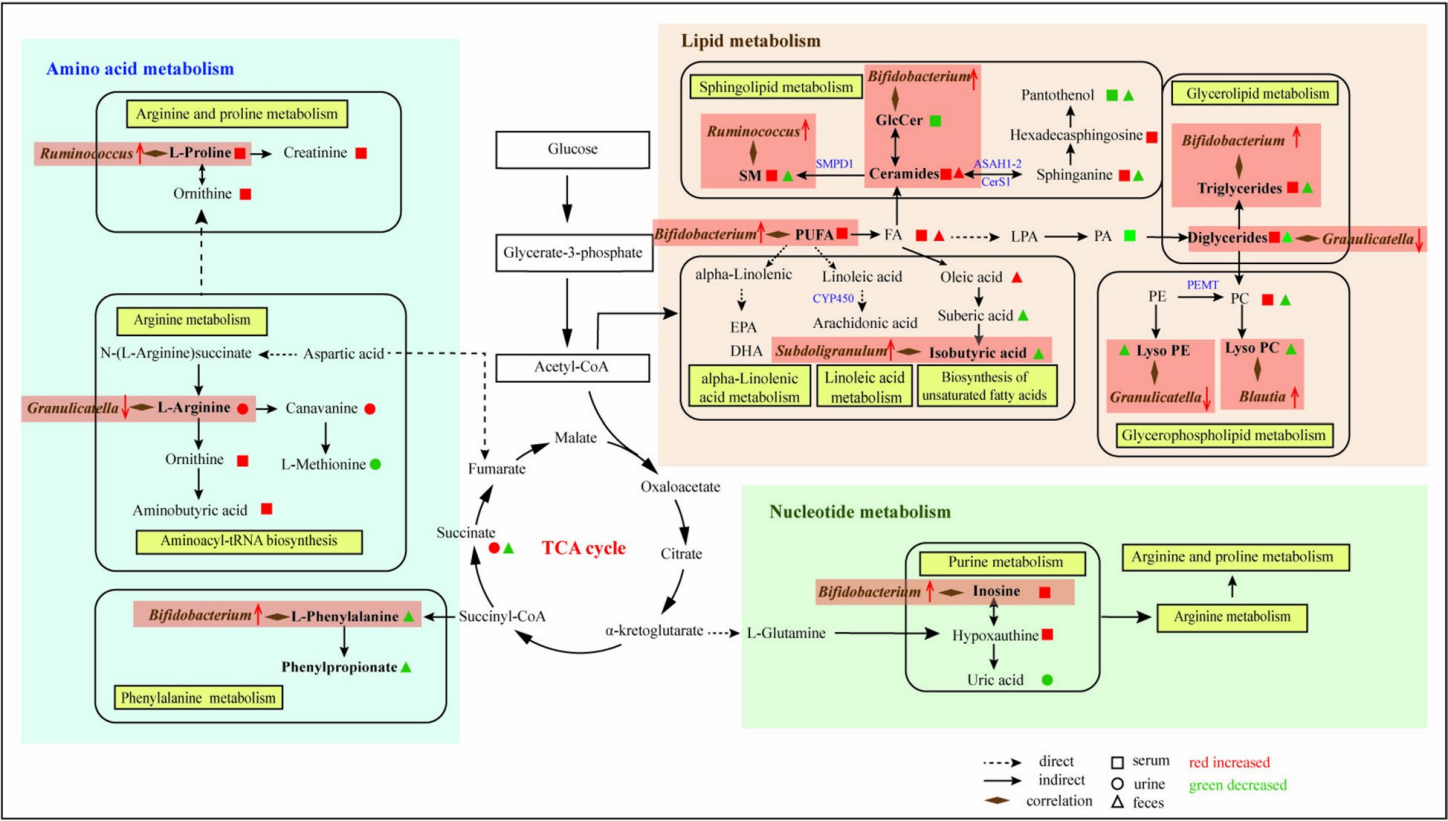
The proposed mechanisms of human immunity regulation by AROL. *FA* fatty acids, *SM* sphingomyelin, *GlcCer* glucosylceramide, *PUFA* polyunsaturated fatty acids, *PA* phosphatidic acid, *LPA* lysophosphatidic acid, *PE* phosphatidylethanolamine, *PC* phosphatidylcholine, *EPA* eicosapentaenoic acid, *DHA* docosahexaenoic acid, *SMPD1* sphingomyelin phosphodiesterase 1, *ASAH1-2* *N*-acyl sphingosine amidohydrolase 1–2, *CerS1* ceramide synthase 1, *CYP450* Cytochrome P450, *PEMT* phosphatidylethanolamine *N*-methyltransferase

Gut microbiota could greatly affect metabolic profiles [35]. The correlation analysis between the 20 major genera and AR-induced PBs showed that no less than 65% of PBs significantly correlated with gut microbiota in all samples. This suggested that AR may regulate the metabolism in the microbiota-related manner, thereby regulating human immunity, just as shown in Fig. 8. Among the various types of correlation between major genera and different PBs, it is worth noting that there were strong correlations between *Bifidobacterium* and AA metabolism, lipid metabolism, and purine metabolism. Therefore, we reasonably proposed that AR could upregulate the abundance of *Bifidobacterium* in human, and modulate metabolic pathways of AAs, lipids, and nucleotides, thereby promoting the proliferation of CD4^+^ and CD8^+^ T cells and the release of cytokines such as IL-4, IL-6, IL-12P70, TNF-α, and IFN-γ, to display the regulation on human immunity.

AAs possessed important functions in both nutrition and health, and exerted an important role in both adaptive and innate immunity by adjusting immune cell activation and antibody formation [36]. Changes in AAs and the subsequent biochemical pathways have increasingly emerged as a key area developing innovative pharmaceuticals and therapeutic targets that may regulate immunity [37]. Just as mentioned above, three AA metabolic pathways corresponding to arginine, proline, and phenylalanine metabolisms were regulated by AR. The previous study has revealed that the activation of phenylalanine catabolic pathway to convert phenylalanine to phenylpyruvate could promote the neutrophil-evasive state [38]. Our observations revealed that several intermediates associated with the aromatic amino acid metabolic pathway, such as L-phenylalanine, 2-phenylpropionate, and phenylacetylglutamine, were notably reduced following AROL administration, indicating the activation of phenylalanine catabolism. At the same time, a negative relationship was noted between the increase of *Bifidobacterium* levels and the decrease in L-phenylalanine. Therefore, we hypothesized that AR could up-regulate *Bifidobacterium* to promote L-phenylalanine catabolism, then weaken human neutrophil chemotaxis, to exert the immunomodulatory effects. Arginine and proline have been proven to regulate the immune function in aquatic animals [39], and the elevated L-arginine concentrations may trigger widespread metabolic alterations, such as a transition from glycolysis to oxidative phosphorylation in activated T cells, thereby facilitating the production of T cells [40]. Our current results demonstrated that AR could significantly upregulate the level of L-arginine and L-proline and downregulate L-methionine production from L-arginine in human urine or feces. It’s important to highlight that the increase of L-proline was found to be positively correlated with *Ruminococcus* in the present study, but no related literature is available currently. In addition, it has been reported that L-methionine may be related to immune response modulation and redox interactions [41], and could decrease the IL-4 level as well as the infiltration of macrophage and lymphocyte. Our current results showed that AR could lead to a decrease in L-methionine in urine samples and an increased trend in IL-4 in serum. So, we could hypothesize that AR may promote L-arginine synthesis and catabolism, to simulate T-cell activation and IL-4 release, and finally modulate human immunity.

Similar to AAs, lipid metabolisms were also widely affected by AROL. Considering the great number of identified lipids PBs as well as their excellent correlation with IIs, lipid metabolisms could even be considered as the priority pathway for AR regulation of human immunity. Generally, five major lipid metabolic pathways were enriched in the present study, including glycerophospholipid metabolism, glycerolipid metabolism, sphingolipid metabolism, SCFA metabolism, and PUFAs metabolisms (alpha-linolenic acid metabolism, linoleic acid metabolism). Glycerophospholipids, as the fundamental components of bio-membranes, are typically involved in signaling pathways and immune responses [42]. The intermediates in glycerophospholipid metabolism PC and LysoPC in feces samples were reduced by AROL, and these changes exhibited a negative correlation with TNF-α, IL-4, and IL-2, and showed a positive correlation with *Blautia*. Accordingly, we inferred that AR could downregulate *Blautia* abundance and the levels of PC and LysoPC, thereby stimulating the release of cytokines (IL-2, IL-4, and TNF-α) to regulate human immunity. Similar to PC and LysoPC, after AROL administration, various glycerolipids including DG and TG were significantly decreased in human feces, but increased in human serum, indicating that AR could promote glycerolipids into the circulation system. As a typical compound in triglyceride metabolism, TG (12:0/8:0/8:0) was significantly up-regulated in serum by AROL, which was positively correlated with *Bifidobacterium*, IL-4, and IL-2. As a result, AR may up-regulate *Bifidobacterium*, promote the production of glycerolipids, simulate IL-4 release, and inhibit IL-2 release. Sphingolipids were able to affect immune function by binding to CD1 protein and have been used as chemical attractants for immune cells [43]. They could change CD4^+^ T cell function by regulating cell activation, survival, and proliferation. As a representative compound in sphingolipids, SM(d18:1/16:1(9Z)) was significantly increased after AROL administration and it was positively correlated with *Ruminococcu*s. We speculated that AR could enrich *Ruminococcus*, promote the synthesis of SM, and further promote CD4^+^ T cell proliferation to exert its immunomodulatory effect. SCFAs were proven to promote the expansion of Treg cells and improve effector T cell functions [44]. Of them, butyrate has the potential to enhance memory capabilities and boost the antiviral cytotoxic functions of CD8+ T cells in mice [45]. AROL could reduce the level of isobutyric acid in feces, which was negatively correlated with Scr and *Subdoligranulum*. Therefore, AR may enrich *Subdoligranulum*, and promote butyrate catabolism, thereby promoting CD8^+^ T cell and inhibiting Scr production, to regulate human immunity. Besides, regulation of PUFA metabolisms including linoleic acid metabolism and alpha-linolenic acid metabolism were considered as the major immunomodulatory mechanisms of APS [46], which was also consistent with our findings, but the specific mechanisms still need further exploration.

Purine nucleoside metabolism was also a metabolic pathway in human immune regulation. Purine nucleosides, including adenosine and its main metabolite inosine, served as essential building blocks for DNA and RNA. Adenosine as a potent signaling molecule engaged with its receptors and significantly influencing the regulation of inflammation [47]. Inosine was synthesized through the activation of the adenosine receptor A2A, and its production can be enhanced by B. *pseudolongum* in the intestine. This process has been shown to improve the response to immunotherapy in mice with colon cancer during anticancer treatment [48]. Inosine has the potential to inhibit the activation of lymphocytes and neutrophil macrophages in vitro, while also mitigating the pro-inflammatory effects of LysoPS in vivo [49]. Consistently, this study revealed that AROL significantly upregulated the serum level of inosine which was positively correlated with *Bifidobacterium*, indicating that AR could up-regulate *Bifidobacterium* to promote the synthesis of inosine, and accordingly attenuate inflammation to exert the immune regulatory effects.

Collectively, the present study revealed that AR could induce significant changes in only six less abundant feces genera, comprehensively promote the anabolism of arginine, glycerolipids, sphingolipids, and purines, as well as the catabolism of phenylalanine and glycerophospholipids in a microbiota-related manner, and accordingly significantly regulate immune cytokines and change immune cells to some extent, thereby exerting the immunomodulation of human. These findings could provide some scientific clinical evidence to support AR as the leader of tonics among herbal medicines and promote the subsequent development of AR-related products as dietary supplements or functional foods. However, our current study also had some limitations or issues to be further addressed. In our current study, only 20 healthy subjects were selected due to time and financial constraints. However, our self-control study design could compensate for the above limitations to some extent, especially considering that each subject normally displayed certain metabolic profiles during a relatively short period like 15 days. Moreover, all 20 selected subjects were aged between 18 and 40 years old, with equal numbers of males and females, which could partly attenuate the inter-individual difference. The results of the current study will be further confirmed through upcoming large-scale clinical trials involving a larger sample size. From the perspective of technology, the results obtained from untargeted metabolomics and lipidomics, including AR-induced PBs and related metabolic pathways, were not always reliable and should be further validated by targeted metabolomics and lipidomics or related molecular mechanism studies. Meanwhile, our current gut microbiome analysis relied heavily on the 16S rRNA-based classification of respective OTUs, which primarily concentrated on the genus level and has not been further explored at the species level. Accordingly, our results may not fully capture the metabolic capabilities of these bacteria, which exhibit significant variation even among the same OTUs, along with their intricate interactions with human hosts and the associated health implications [50]. Additionally, further research on the microbiota is necessary to elucidate the specific roles of the six differential genera, as well as major genera like *Bifidobacterium*, in the immunomodulation of AR. Moreover, the correlation among the IIs, dominant genera, and PBs was just simply constructed by Spearman’s correlation analysis, and the corresponding results needed to be further validated. From the perspective of study design, the diet of the volunteers was not controlled during the period of the population experiment, and inclusion criteria did not well match the studies on gut microbiota, which, to some extent, may influence the reliability of the findings in our present study. However, considering that each subject normally displayed certain diet habits and stable gut microbiota, our self-control study design could greatly reduce the risk of producing unreliable results. Meanwhile, all biological samples were collected after being fasted for approximately 12 h (from 8 p.m. to 8 a.m. of the next day), which could reduce the impact of dietary intake on our results. Despite this, further similar investigations should consider controlling the diet of subjects in an inpatient setting and adopting more prudent inclusion criteria to control the variation of gut microbiota between subjects.

## Conclusion

In the present study, a comprehensive analysis of metabolomics and lipidomics of human serum, urine, and feces was combined with gut microbiota profiling, to clarify the immunomodulatory effects of AR and the associated mechanisms in healthy Chinese individuals for the first time. Fortnight successive administration of AROL showed excellent safety to all 20 healthy volunteers. AROL could remarkably down-regulate Scr and pro-inflammatory cytokines, and up-regulate anti-inflammatory cytokines while slightly change the global metabolic profiles, activate T cells, and inhibit NK cells and B cells, leading to immunomodulation effects on humans. AR could significantly promote the anabolism of arginine, glycerophospholipid, sphingolipid, and purine, and the catabolism of phenylalanine and glycerophospholipid. As for gut microbiota, AR could not only remarkably down-regulate the abundance of *Granulicatella*, but also change major genera such as *Bifidobacterium*, *Ruminococcus*, *Blautia*, and *Subdoligranulum*, which were well correlated with both the AR-induced PBs and significant IIs. The present study not only provided reliable clinical evidence to support AR as a promising immunomodulatory tonic, but also greatly promoted and facilitated the growth of AR-related MFH, functional foods, and dietary supplements.

## Supplementary Information

Supplementary Material 1.

## Data Availability

Data will be made available on request.

## Abbreviations

MFH: Medicinal food homology
AR: *Astragali radix*
TCM: Traditional Chinese Medicine
OTC: Over the counter
APS: Astragalus polysaccharide
UHPLC-Q-TOF/MS: Ultra-High-Performance Liquid Chromatography coupled with Quadrupole-Time-Of-Flight Mass Spectrometry
MA: Metabolomics analysis
LA: Lipidomics analysis
SCFAs: Short chain fatty acids
PBs: Potential biomarkers
IIs: Immune indicators
AROL: Astragali radix oral liquid
BMI: Body Mass Index
SBP: Systolic blood pressure
DBP: Diastolic blood pressure
WBC: White blood cell count
RBC: Red blood cell count
HGB: Hemoglobin
PLT: Platelet
NEUT: Neutrophil ratio
LYM: Lymphocyte
AST: Aspartate aminotransferase
ALT: Alanine aminotransferase
Scr: Serum creatinine
BUN: Blood urea nitrogen
T cells: Thymus dependent lymphocytes
B cells: Bursa dependent lymphocytes
NK cells: Natural killer cells
HILIC-Q-TOF/MS: Hydrophilic Interaction Liquid Chromatography-Q-TOF/MS
RPLC-Q-TOF/MS: Reverse-Phase Liquid Chromatography-Q-TOF/MS
QC: Quality control
TICs: Total ion chromatograms
PCA: Principal components analysis
PLS-DA: Partial Least-Squares Discriminant Analysis
TUMS: Total useful MS signals
SERRF: Systematic error removal using random forest
VIP: Variable importance in projection
FC: Fold change
SMFs: Significant molecular features
HMDB: Human metabolome database
OTU: Operational taxonomic unit
NMDS: Non-metric multidimensional scaling
ANOVA: Analysis of variance
TG: Triglycerides
DG: Diglycerides
Cer: Ceramides
SFAs: Saturated fatty acids
PC: Phosphatidylcholine
SM: Sphingomyelins
LysoPE: Lyso phosphatidylethanolamine
PUFAs: Polyunsaturated fatty acids
