# Supplementary material for: Integrated analysis of metabolome, lipidome, and gut microbiome reveals the immunomodulation of Astragali radix in healthy human subjects
Source: Chin Med. 2024 Dec 19;19:174. doi: 10.1186/s13020-024-01045-2 (PMC11657124; doi:10.1186/s13020-024-01045-2)
Supplement: Supplementary file 1 — Supplementary Material 1. [file 13020_2024_1045_MOESM1_ESM.docx]

**Supplementary Information:**

## S1 Instrumentation and conditions for the chemical analysis of AROL

The UPLC separation was performed on a series Agilent 1290 Infinity Ultra Performance Liquid Chromatography system equipped with a binary pump (G4220A), a column Oven (G1316C), an autosampler (G4226A), and a temperature control module for the automatic sampler (G1330B), using a Waters ACQUITY UHPLC HSS T3 column (2.1×100 mm, 1.8 μm). The mobile phase consisted of water containing 0.1% formic acid (A) and acetonitrile containing 0.1% formic acid (B), with a gradient program as follows: 0-3.5 min, 99% A; 3.5-8 min, 99%-80% A; 8-10 min, 80%-78% A; 10-13 min, 78%-75% A; 13-14min, 75%-60% A; 14-22 min, 60%-35% A; 22-25 min, 35% A; 25-26 min, 35%-99% A; 26-31 min, 99% A, at a flow rate was 0.4 mL/min and the temperature of the column was maintained at 30°C. The injection volume was 5 μL and the autosampler temperature was 4°C. Mass detection was performed on the Triple TOF 5600 (AB SCIEX, USA), a hybrid triple Q-TOF mass spectrometer equipped with an ESI source. Both positive and negative ion modes were employed to provide the abundant adduct ions for validating the presence of each potential compound, as well as the fragment ions for structural characterization. The conditions of the TOF/MS scan type were as follows: TOF masses range was set at m/z 80–1200; accumulation time: 0.15 s; ion source gas 1: 60 psi; ion source gas 2: 60 psi; curtain gas: 35 psi; temperature: 500°C; declustering potential: 80 V; collision energy: 10 eV; ion spray voltage floating was set at 4500 and 5500 V in the negative and positive ion mode, respectively. The options of information-dependent acquisition (IDA), dynamic background subtraction, and high sensitivity mode were chosen. Major IDA switch criteria were as follows: intensity exceeds 50 cps, exclusion isotope within 4 Da, mass tolerance 50 mDa, and the maximum number of candidate ions to monitor per cycle 12. For the product ion scan type, the parameters were almost the same as the TOF-MS scan type except that the TOF masses range was set at m/z 50-1200, accumulation time was set at 0.035 s, and collision voltage was set at 40 ± 20 eV. A mass spectrometry automatic calibration delivery system (AB SCIEX) was employed to auto-calibrate the mass accuracy of the detection per 2 h, by using APCI positive and negative calibration solutions for the AB SCIEX Triple TOF system. All the operations and acquisitions were controlled by Analyst TF 1.6 software (AB SCIEX).

## S2 Methods for the detection of immune cells and immune cytokines

The human 12-item cytokine assay kit (Qingdao Raisecare Biotechnology Co., Ltd., RAISECARE, China) was used to detect the serum levels of eight immune cytokines including IL-2, IL-6, IL-10, IFN-γ, IL-17, IL-4, IL-12P70, and TNF-α. 25 μL each of substrate, standard, capture microsphere antibody, and detection antibody were mixed thoroughly, and then put into an incubator and incubated for 2 h, protected from light and at room temperature. Add 25 μL of SA-PE, incubate for 0.5 h, add 500 μL washing buffer, vortex for a few seconds, centrifuge at 300-500 *g* for 5 min, pour out the liquid slowly, invert on absorbent paper, and add 150 μL-300 μL of washing buffer to each tube for testing (Beckman Coulter EPICS XL flow cytometer). Subsequently, 25 μL each of the experimental buffer, sample, capture microspheres, and detection antibody were added to each sample tube and mixed thoroughly as described above. All steps in this experiment were performed according to the manufacturer's instructions.

The Beckman Coulter EPICS XL flow cytometer (Beckman Coulter, Miami, Fla.) was utilized for flow cytometer analysis, to describe the condition of all three types of immune cells including Thymus dependent lymphocytes (T cells), Bursa dependent lymphocytes (B cells), and Natural Killer (NK) cells. Especially, CD19+ (corresponding to B cells), CD3-CD16+CD56+ (corresponding to NK cells), CD3+ (corresponding to T cells), and two types of T cells including CD3+CD4+CD8- (CD4+) and CD3+CD4-CD8+ (CD8+), were included to assess the human immunomodulatory effects of AROL. Flow cytometer alignment, calibration, and spectral compensation were performed according to the laboratory’s operating procedure. For specimen preparation, 100 mL of EDTA-anticoagulated blood was incubated with premixed antibodies for 20 min and erythrocytes were lysed with the ImmunoPrep reagent system (Beckman Coulter) on a Multi-Q-Prep workstation and analyzed within 6 h of lysis. The percentage of lymphocytes expressing CD4+ T cells or CD8+ T cells was determined according to the published guidelines for Flow Cytometric Immunophenotyping. A lymphocyte light scatter gate was manually drawn on a dot plot of side versus forward light scatter parameters. All operations and analysis were performed using System II software and the tetraONE System (version 1.0; Beckman Coulter).

## S3 Methods for RPLC/HILIC-Q-TOF/MS analysis

S3.1 RPLC-Q-TOF/MS analysis

For lipidome profiling, chromatographic separation was conducted utilizing a Waters ACQUITY UPLC^®^ CSH^TM^ C18 column (2.1 × 100 mm, 1.7 μm). The temperature of the column was kept constant at 65°C, while the autosampler was configured to operate at 4°C. The mobile phase A consists of 5 mM ammonium formate in a mixture of acetonitrile and water (6:4, *v/v*), whereas mobile phase B is composed of isopropanol and acetonitrile (9:1, *v/v*), also containing 5 mM ammonium formate. Gradient elution was conducted at a constant flow rate of 0.4 mL/min as detailed below: 0-2 min, 15%-30% B; 2-2.5 min, 30%-48% B; 2.5-11 min, 48%-82% B; 11-11.5 min, 82%-99% B; 11.5-12 min, 99% B; 12-12.1 min, 99%-15% B; 12.1-15 min, 15% B, and the injection volume was 2 μL.

The parameters for the TOF-MS scan type were established as follows: the mass range was adjusted to *m/z* 50-1500; the accumulation time was set to 0.15 s; ion source gas 1 and gas 2 were both configured at 60 psi; the curtain gas was at 35 psi; the temperature was fixed at 600°C; declustering potential was set to 80 V; collision energy was 10 eV; and the ion spray voltage was configured to 4500 V for negative-ion mode and 5500 V for positive-ion mode. The options of IDA, dynamic background subtraction, and high sensitivity were chosen. The primary criteria for IDA switching were established as follows: Intensity exceeds 50 cps, exclusion isotope within 4 Da, mass tolerance 50 mDa, and a maximum of 12 candidate ions to monitor during each cycle. The Product ion scan type had almost identical specifications except for the TOF mass range (*m/z* 25-1000), accumulation time (0.035 s), and collision voltage (40 ± 20 eV).

S3.2 HILIC-Q-TOF/MS analysis

Serum and feces HILIC-Q-TOF/MS analysis shared the common chromatographic conditions. Chromatographic separation was performed on a Waters ACQUITY UPLC^®^ BEH Amide column (2.1 × 150 mm, 1.7 μm) at 45°C, with a gradient elution consisting of phase A (5 mM ammonium formate in water) and phase B (5 mM ammonium formate in acetonitrile: water (95:5, *v/v*) at a constant flow rate of 0.25 mL/min. The gradient system was optimized as follows: 0-2 min, 100% B; 2-7.7 min, 100%-70% B; 7.7-9.5 min, 70%-40% B; 9.5-10.25 min, 40%-30% B; 10.25-12.75 min, 30%-100% B; 12.75-17 min, 100% B. An injection volume of 3 μL was employed.

Urine MA employed another chromatographic condition equipped with a Waters ACQUITY UPLC^®^ HSS T3 column (2.1 × 100 mm, 1.8 μm) at 35°C. The autosampler was set at 4°C, while the mobile phase consisted of two components: (A) 5 mM ammonium formate dissolved in water and (B) a mixture of 5 mM ammonium formate in acetonitrile and water (95:5, *v/v*). The flow rate was 0.25 mL/min, with an injection volume of 5 μL. The gradient was set as follows: 0-2 min, 1% B; 2-19 min, 1%-20% B; 19-20 min, 20%-30% B; 20-25 min, 30%-50% B; 25-26 min, 50%-80% B; 26-28 min, 80% B; 28-29 min, 80%-1% B; 29-35min, 1% B.

For HILIC-Q-TOF/MS analysis, the TOF mass ranges in both the TOF-MS scan type and the Product ion scan type were set at *m/z* 50-1000, while the remaining Mass Spectrometry settings were the same as for RPLC-Q-TOF/MS.

**Figure legends**

**Figure S1.** The UHPLC-Q-TOF/MS total ion chromatogram of AROL. (A) Positive ion mode. (B) Negative ion mode.

**Figure S2.** Flow cytometry analysis of human blood immune cells after AROL administration. (A) CD4+ (T cell), CD8+ (T cell); (B) CD3+ (T cell), CD19+ (B cell); (C) CD3-CD16+CD56+ (NK cell); (D) Changes in the proportion of immune cells in Group d0 and Group d15.

**Figure S3.** The overlaid total ion chromatograms from pooled QC samples of human serum, urine, and feces. (A) Serum LA pos; (B) Serum LA neg; (C) Serum MA pos; (D) Serum MA neg; (E) Urine pos; (F) Urine neg; (G) Feces LA pos; (H) Feces LA neg; (I) Feces MA pos; (J) Feces MA neg; (QC, Quality Control; LA: Lipidomics Analysis; MA: Metabolomics Analysis; pos: Positive mode; neg: Negative mode.)

**Figure S4.** PCA score plots (before SERRF) of Group d0 and Group d15. (A) Serum LA pos; (B) Serum LA neg; (C) Serum MA pos; (D)Serum MA neg; (E) Urine pos; (F) Urine neg; (G) Feces LA pos; (H) Feces LA neg; (I) Feces MA pos; (J) Feces MA neg; (LA: Lipidomics Analysis; MA: Metabolomics Analysis.)

**Figure S5.** Cluster analysis of metabolites after the administration of AROL (PLS-DA score plots). (A) Serum LA pos, R^2^Y=0.993, Q^2^Y=0.744; (B) Serum LA neg, R^2^Y=0.762, Q^2^Y=0.394; (C) Serum MA pos, R^2^Y=0.929, Q^2^Y=0.529; (D)Serum MA neg, R^2^Y=0.98, Q^2^Y=0.471; (E) Urine pos, R^2^Y=0.857, Q^2^Y=0.17; (F) Urine neg, R^2^Y=0.858, Q^2^Y=0.158; (G) Feces LA pos, R^2^Y=0.59, Q^2^Y=0.0771; (H) Feces LA neg, R^2^Y=0.79, Q^2^Y=0.11; (I) Feces MA pos, R^2^Y=0.867, Q^2^Y=0.249; (J) Feces MA neg, R^2^Y=0.955, Q^2^Y=0.224; (LA: Lipidomics Analysis; MA: Metabolomics Analysis.)

**Figure S6.** Volcano analysis of metabolites between two groups (Volcano plots). (A) Serum LA pos; (B) Serum LA neg; (C) Serum MA pos; (D)Serum MA neg; (E) Urine pos; (F) Urine neg; (G) Feces LA pos; (H) Feces LA neg; (I) Feces MA pos; (J) Feces MA neg; (LA: Lipidomics Analysis; MA: Metabolomics Analysis.) (*p*<0.05, FC<1.2 or FC>0.83)

**Figure S7.** Histogram analysis of the (A) *p*-value and (B) FC-value of AROL-induced PBs in serum, urine, and feces samples.

**Figure S8.** Venn diagrams showing unique or overlapped modulation of three samples on AROL-induced (A) PBs, (B) pathways, and (C) metabolic clusters of healthy humans.

**Figure S9**. Characterization of gut microbiota. (A) The rarefaction curves on sobs index. (B) The pan analysis and (C) core analysis.

**Figure S10.** Alpha diversity analysis. (A) Ace index. (B) Chao index. (C) Shanno index. (D) Simpson index.

**Table legends**

**Table S1** The information of standard compounds in AR.

**Table S2.** Representative compounds of AROL identified by UHPLC-Q-TOF-MS.

**Table S3.** Changes in physiological functions, biochemical functions, immune cells, and immune cytokines between Group d0 and Group d15. (**p*<0.05, ***p*<0.01, ****p*<0.001)

**Table S4.** Reliability of analytical methods based on the RSD% values of the RT, m/z, and Intensity by UHPLC-Q-TOF/MS.

**Table S5.** Changes in the number of features during data processing.

**Table S6.** The list of 89 immunomodulation PBs of AROL in serum.

**Table S7.** The list of 41 immunomodulation PBs of AROL in urine.

**Table S8.** The list of 104 immunomodulation PBs of AROL in feces.


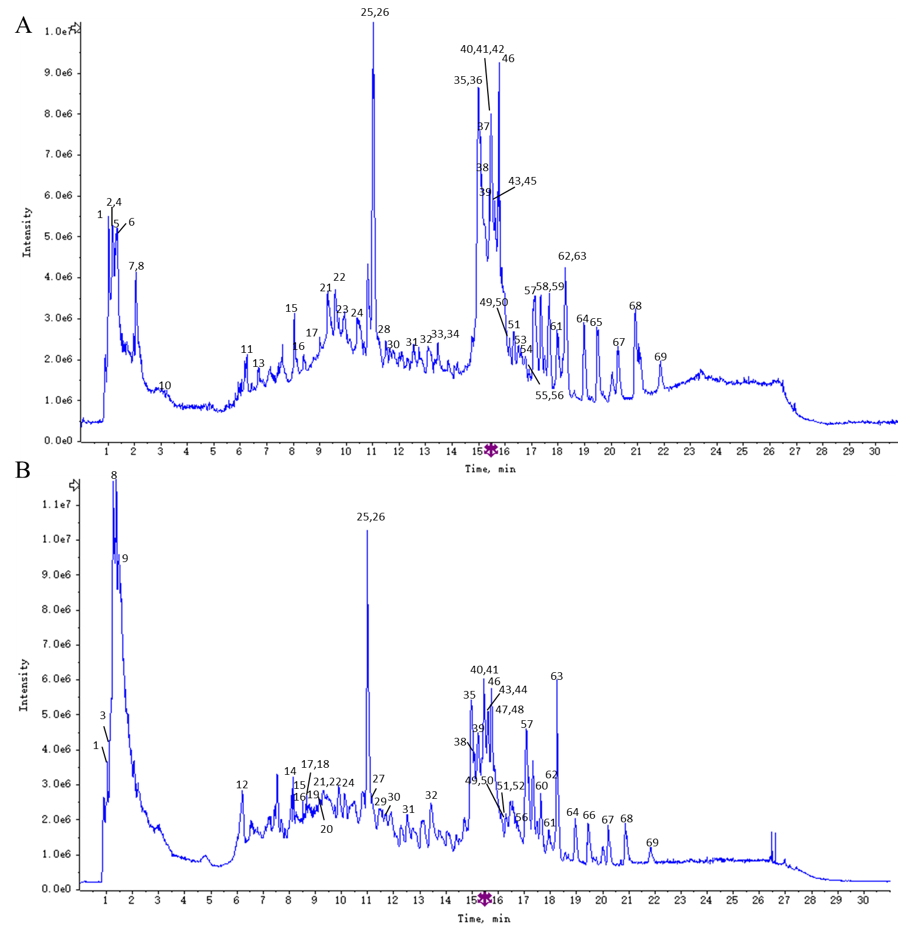


Figure S1. The UHPLC-Q-TOF/MS total ion chromatogram of AROL. (A) Positive ion mode. (B) Negative ion mode.


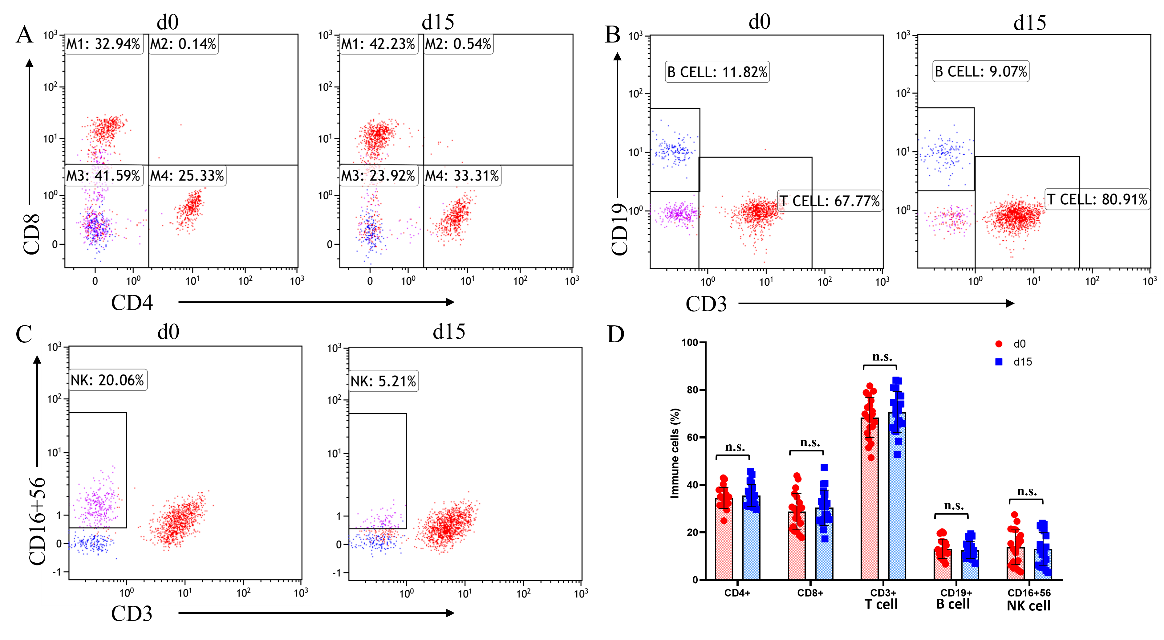


## Figure S2. Flow cytometry analysis of human blood immune cells after AROL administration. (A) CD4+ (T cell), CD8+ (T cell); (B) CD3+ (T cell), CD19+ (B cell); (C) CD3-CD16+CD56+ (NK cell); (D) Changes in the proportion of immune cells in Group d0 and Group d15.


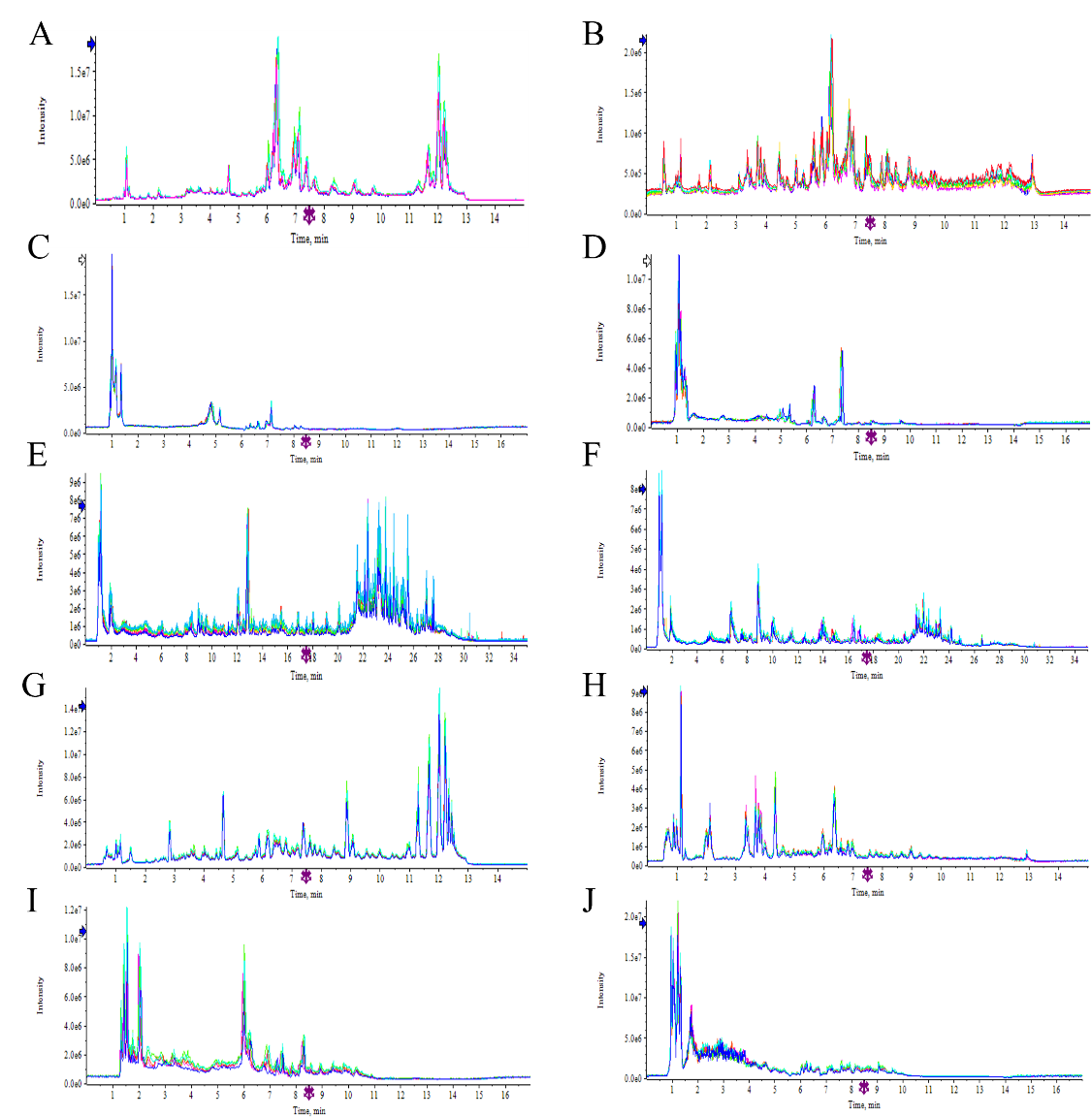


## Figure S3. The overlaid total ion chromatograms from pooled QC samples of human serum, urine, and feces. (A) Serum LA pos; (B) Serum LA neg; (C) Serum MA pos; (D) Serum MA neg; (E) Urine pos; (F) Urine neg; (G) Feces LA pos; (H) Feces LA neg; (I) Feces MA pos; (J) Feces MA neg; (QC, Quality Control; LA: Lipidomics Analysis; MA: Metabolomics Analysis; pos: Positive mode; neg: Negative mode.)


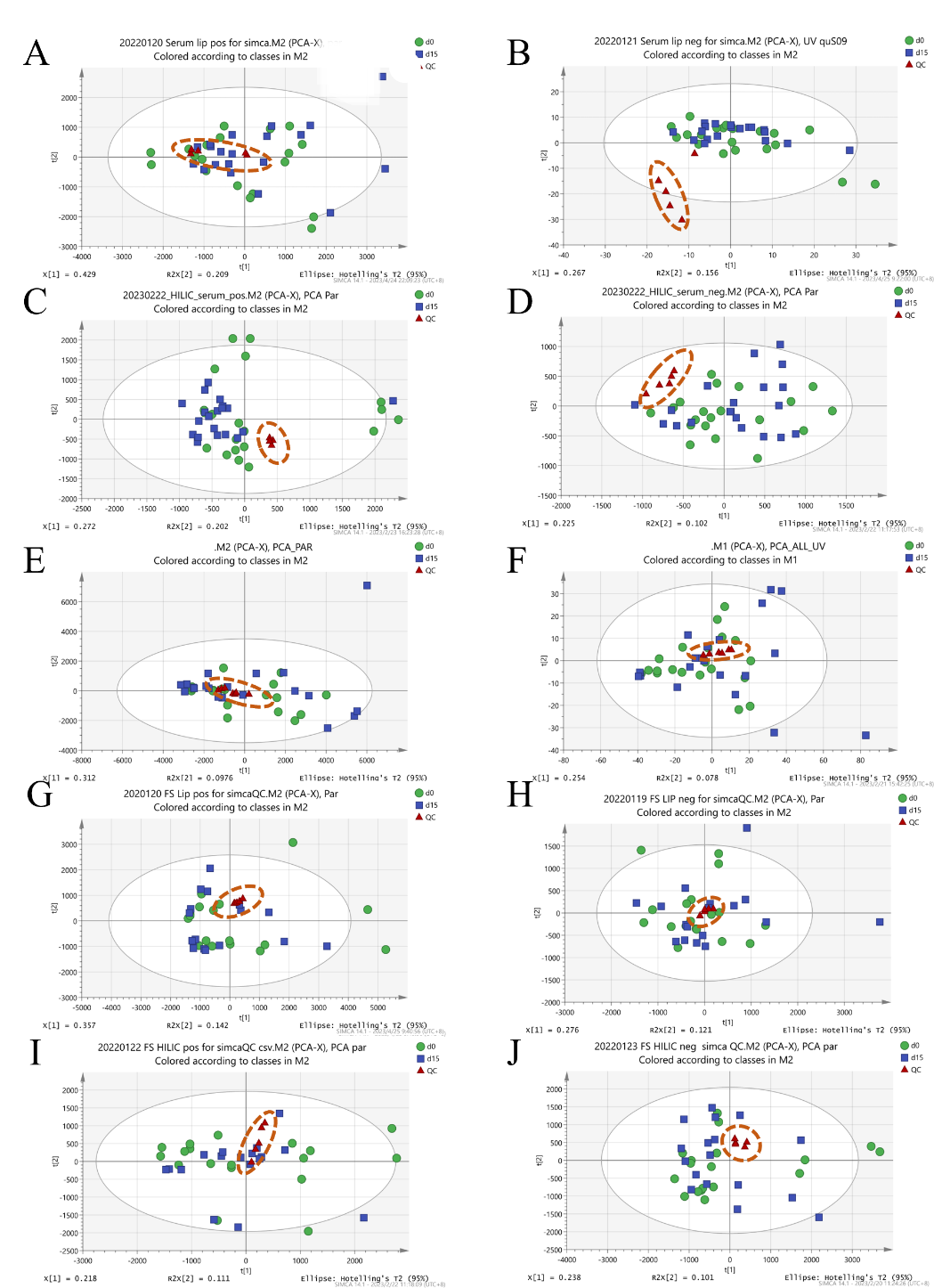


## Figure S4. PCA score plots (before SERRF) of Group d0 and Group d15. (A) Serum LA pos; (B) Serum LA neg; (C) Serum MA pos; (D)Serum MA neg; (E) Urine pos; (F) Urine neg; (G) Feces LA pos; (H) Feces LA neg; (I) Feces MA pos; (J) Feces MA neg; (LA: Lipidomics Analysis; MA: Metabolomics Analysis.)


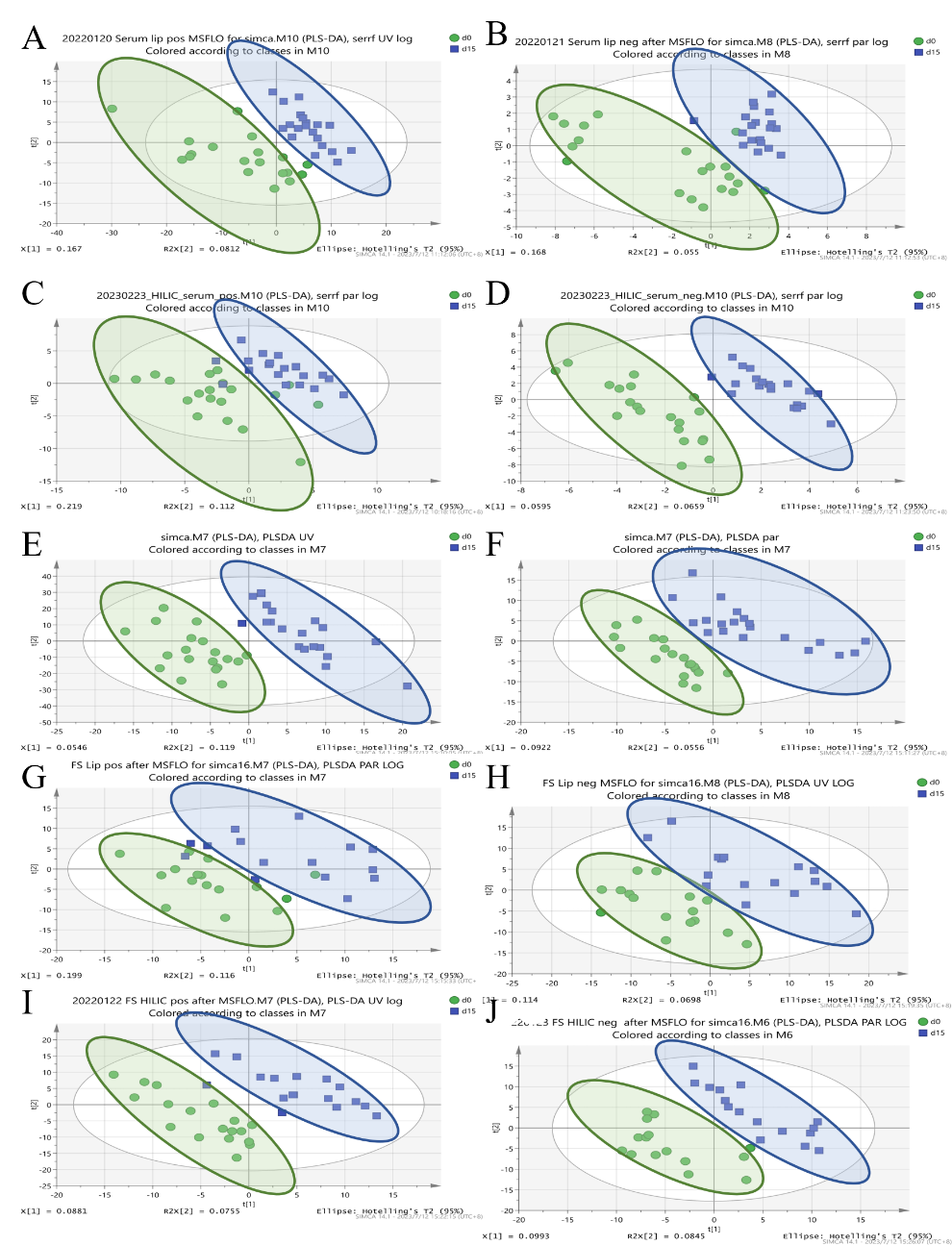


## Figure S5. Cluster analysis of metabolites after the administration of AROL (PLS-DA score plots). (A) Serum LA pos, R^2^Y=0.993, Q^2^Y=0.744; (B) Serum LA neg, R^2^Y=0.762, Q^2^Y=0.394; (C) Serum MA pos, R^2^Y=0.929, Q^2^Y=0.529; (D)Serum MA neg, R^2^Y=0.98, Q^2^Y=0.471; (E) Urine pos, R^2^Y=0.857, Q^2^Y=0.17; (F) Urine neg, R^2^Y=0.858, Q^2^Y=0.158; (G) Feces LA pos, R^2^Y=0.59, Q^2^Y=0.0771; (H) Feces LA neg, R^2^Y=0.79, Q^2^Y=0.11; (I) Feces MA pos, R^2^Y=0.867, Q^2^Y=0.249; (J) Feces MA neg, R^2^Y=0.955, Q^2^Y=0.224; (LA: Lipidomics Analysis; MA: Metabolomics Analysis.)


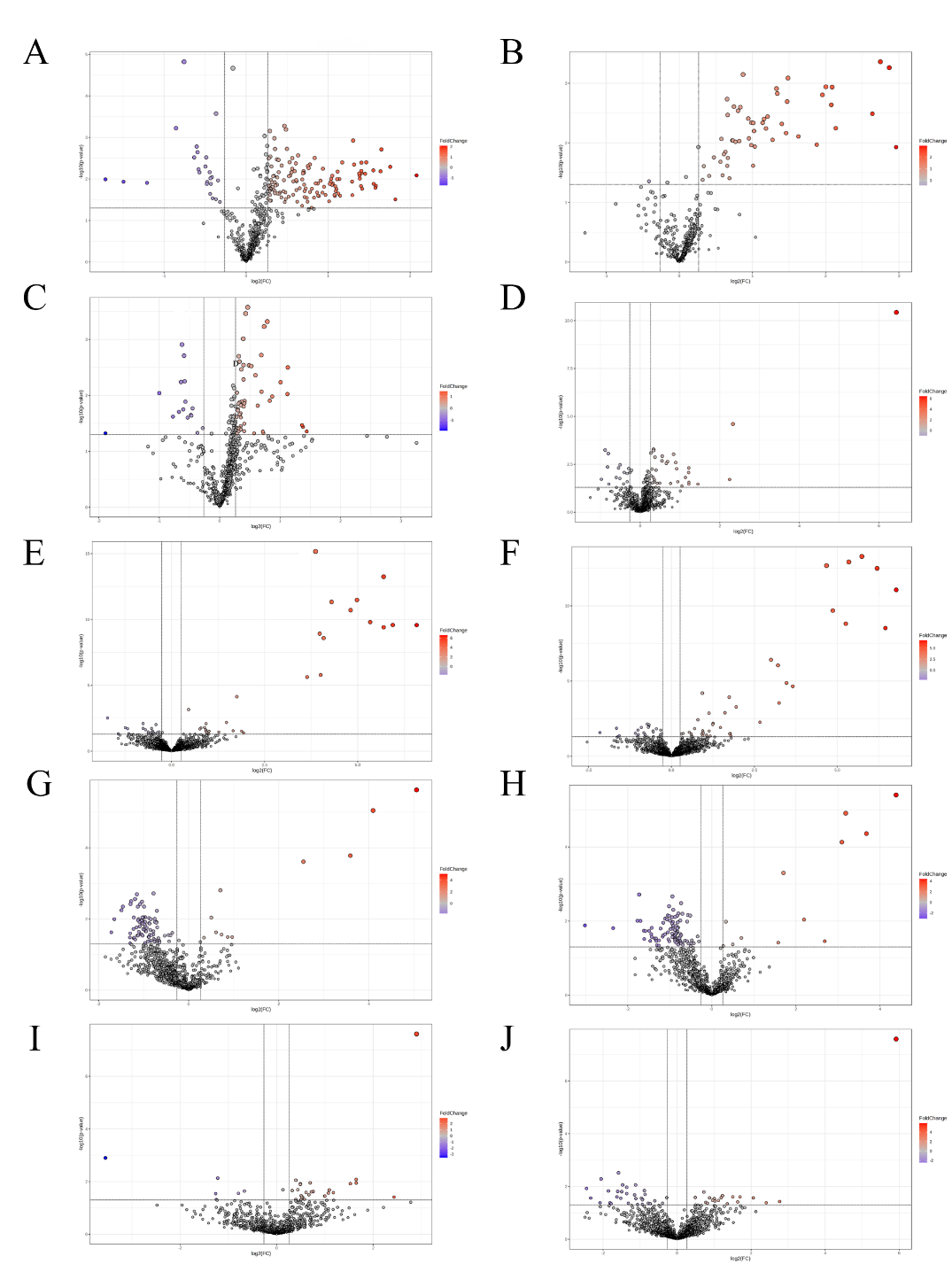


## Figure S6. Volcano analysis of metabolites between two groups (Volcano plots). (A) Serum LA pos; (B) Serum LA neg; (C) Serum MA pos; (D)Serum MA neg; (E) Urine pos; (F) Urine neg; (G) Feces LA pos; (H) Feces LA neg; (I) Feces MA pos; (J) Feces MA neg; (LA: Lipidomics Analysis; MA: Metabolomics Analysis.) (*p*<0.05, FC<1.2 or FC>0.83)


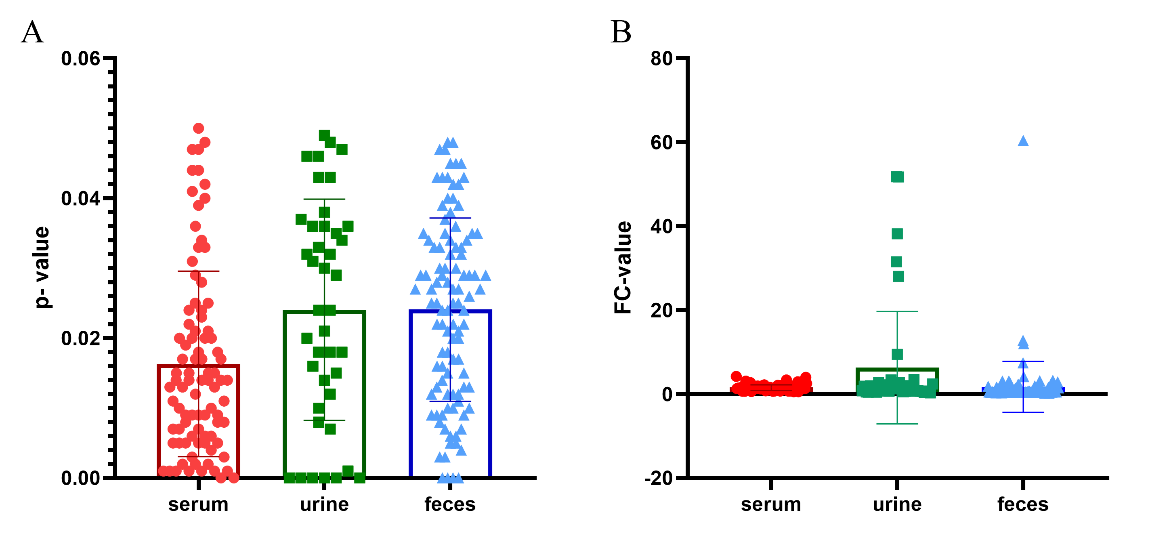


## Figure S7. Histogram analysis of the (A) p-value and (B) FC-value of AROL-induced PBs in serum, urine, and feces samples.


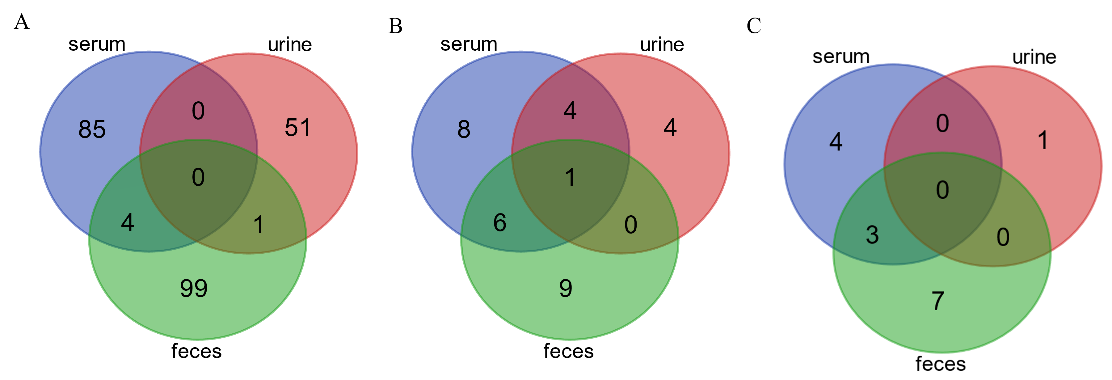


## Figure S8. Venn diagrams showing unique or overlapped modulation of three samples on AROL-induced (A) PBs, (B) pathways, and (C) metabolic clusters of healthy humans.


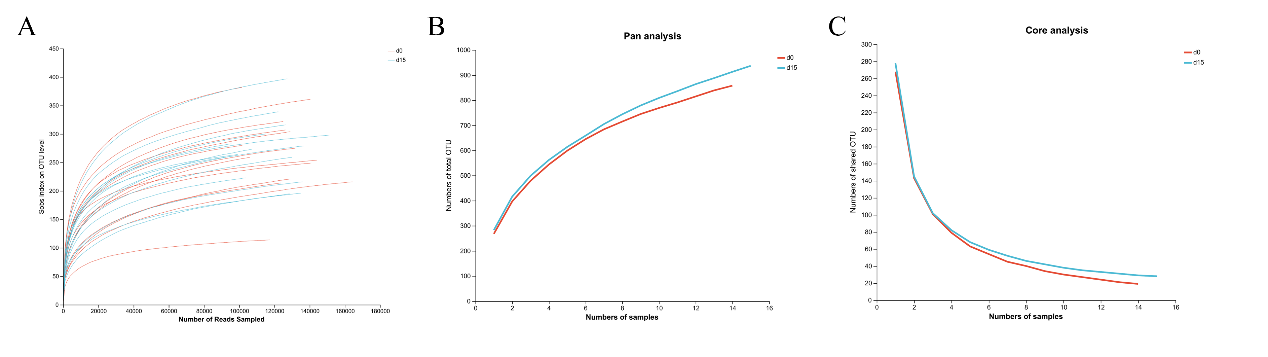


## Figure S9. Characterization of gut microbiota. (A) The rarefaction curves on sobs index. (B) The pan analysis and (C) core analysis.


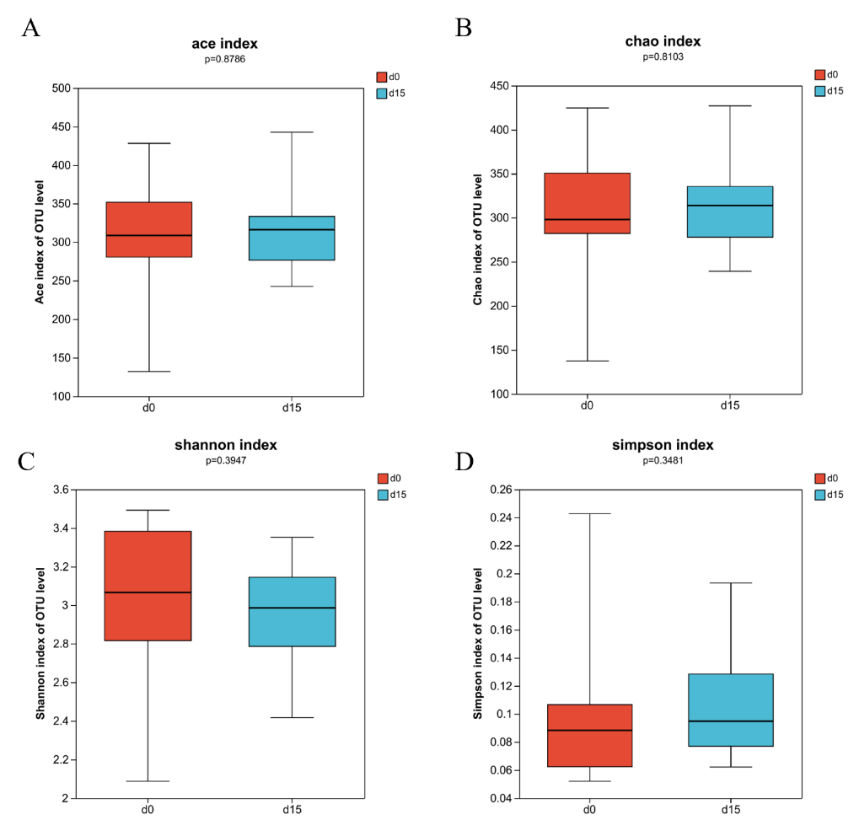


## Figure S10. Alpha diversity analysis. (A) Ace index. (B) Chao index. (C) Shanno index. (D) Simpson index.

## Table S1 The information of standard compounds in AR. (The purity of each standard compound was above 98%.)

| No. | Name | CAS Number | Source | Lot Number |
| --- | --- | --- | --- | --- |
| 1 | Soyasaponin I | 51330-27-9 | Nanjing Liangwei BioTechnology Ltd (Nanjing, China) | lw18020501 |
| 2 | Astragaloside II | 84676-89-1 |  | lw17110607 |
| 3 | Isoferulic acid | 537-73-5 |  | lw17112706 |
| 4 | Astraisoflavan-7-O-β-D-glucoside | 94367-42-7 |  | lw18012612 |
| 5 | 3-Hydoxy-9,10-dimethoxyptercarpan | 73340-41-7 |  | lw18020205 |
| 6 | Astragaloside IV | 84687-48-4 |  | lw18013010 |
| 7 | 7,2'-dihydroxy-3,4'-dimethoxyisofiavane | 94367-43-8 |  | lw18020104 |
| 8 | Formononetin | 485-72-3 |  | lw17090801 |
| 9 | Isoastragaloside | 86764-11-6 |  | lw18020103 |
| 10 | Astragaloside I | 84680-75-1 |  | lw17100910 |
| 11 | L-Phenylalanine | 63-91-2 | Chengdu Aiboke BioTechnology Ltd (Chengdu, China) | AF20080254 |
| 12 | L-Tryptophan | 73-22-3 |  | AF21040653 |
| 13 | Citric acid | 77-92-9 |  | AF9102004 |
| 14 | Sucrose | 57-50-1 |  | AF20110803 |
| 15 | L-Tyrosine | 60-18-4 |  | AF20080252 |
| 16 | 4-Hydroxybenzoic acid | 99-96-7 | Shanghai Yuanye Bio-Technology Ltd (Shanghai, China) | JBZ-0291 |
| 17 | Calycosin | 20575-57-9 |  | Y05J11H115098 |
| 18 | Ononin | 486-62-4 |  | 136531 |
| 19 | Calycosin-7-glucoside | 20633-67-4 | National Institutes for Food and Drug Control (Beijing, China) | M-020-150130 |
| 20 | Isoastragaloside I | 84676-88-0 | Chengdu Must Bio-Technology Ltd (Chengdu, China) | MUST-21050103 |
| 21 | Isoastragaloside IV | 136033-55-1 |  | MUST-20081706 |
| 22 | Cyclocephaloside II | 215776-78-6 |  | MUST-20060607 |
| 23 | Astragaloside III | 84687-42-3 |  | MUST-21050511 |

## Table S2 Representative compounds of AROL identified by UHPLC-Q-TOF-MS..

| NO. | t*_R_* (min) | Ion type | Extraction Mass(m/z) | Observed Mass(m/z) | Error (ppm) | Intensity | Formula | Name | Fragment ions (*m/z*, Area%) |
| --- | --- | --- | --- | --- | --- | --- | --- | --- | --- |
| 1 | 1.05 | [M+H]+ | 175.11895 | 175.11894 | -0.1 | 508037 | C_6_H_14_N_4_O_2_ | L-Arginine | 175.1183(7.36),70.0684(57.12),60.0594(8.18) |
| 2 | 1.11 | [M+H]+ | 120.06552 | 120.06558 | 0.5 | 45930 | C_4_H_9_NO_3_ | Threonine | 120.0662(2.7),74.0626(26.2),74.0252(3.54),56.0537(60.06),55.0217(0.95) |
| 3 | 1.12 | [M-H]- | 146.04588 | 146.047 | 7.7 | 39000 | C_5_H_9_NO_4_ | DL-Glutamic acid | 146.0475(16.19),128.0355(25.58),102.0576(49.56),85.0295(2.59),84.0449(1.86),74.0261(3.07),56.0545(1.14) |
| 4 | 1.19 | [M+H]+ | 325.11292 | 325.11285 | -0.2 | 209657 | C_12_H_20_O_10_ | Lactosan | 163.0603(5.05),145.0504(13.94),127.04(21.29),115.0403(1.76),101.025(1.6),99.047(1.39),97.0302(5.18),85.0308(16.44),69.0368(7.01),57.0376(2.81),55.0222(1.94) |
| 5 | 1.28 | [M+H]+ | 116.07061 | 116.07081 | 1.8 | 280720 | C_5_H_9_NO_2_ | DL-proline | 116.0713(4.29),70.0679(84.37),69.9345(0.51),69.8532(1.02),68.0521(5.38),53.0435(0.95) |
| 6^*^ | 1.41 | [M+H]+ | 343.12349 | 343.12334 | -0.4 | 23647 | C_12_H_22_O_11_ | Sucrose | 163.0601(12.42),145.0504(17.84),127.0392(16.05),115.0442(2.06),109.0297(3.41),97.0305(3.33),85.0314(25.13),69.0357(4.31),57.0354(1.37) |
| 7 | 2.06 | [M+H]+ | 124.0393 | 124.03925 | -0.4 | 74603 | C_6_H_7_N_5_O | 7-Methylguanine | 124.0412(27.19),123.6631(0.41),122.0227(1.15),106.0315(1.4),94.0306(0.46),80.0534(18.72),79.8063(0.34),79.0439(3.24),78.7988(0.4),78.0585(0.2),78.0377(19.67),77.8788(0.15),77.8352(0.32),77.7957(0.62),53.0862(0.09),53.0659(0.36),53.0597(0.14),53.0433(6.5),52.0543(0.32),52.024(9.69),51.0286(5.57),51.0152(0.32),50.0336(0.21),50.0199(2.51) |
| 8^*^ | 2.11 | [M-H]- | 191.01973 | 191.02041 | 3.6 | 1019931 | C_6_H_8_O_7_ | Citric acid | 129.0202(3.05),111.0109(28.26),87.0112(24.19),86.7388(0.59),85.0324(11.54),67.0223(9.33),59.0166(1.3),57.0384(8.9) |
| 9^*^ | 2.37 | [M+NO3]- | 243.06116 | 243.06265 | 6.1 | 59772 | C_9_H_11_NO_3_ | L-Tyrosine | 243.0591(12.23),200.0584(13.27),152.0354(6.64),140.0367(3.64),1122.0261(5.42),111.0218(6.16),110.0256(22.1),82.0315(11.41),68.0155(3.68),66.0377(5.75) |
| 10 | 3.05 | [M+H]+ | 245.07681 | 245.0762 | -2.5 | 19523 | C_9_H_12_N_2_O_6_ | Uridine | 152.0567(60.6),135.031(29.68) |
| 11 | 6.07 | [M+H]+ | 284.09895 | 284.09871 | -0.8 | 149112 | C_10_H_13_N_5_O_5_ | Guanosine | 113.0354(67.1),96.0102(7.3),70.0315(9),69.0364(0.95) |
| 12^*^ | 6.69 | [M-H]- | 164.0717 | 164.07244 | 4.5 | 88974 | C_9_H_11_NO_2_ | L-Phenylalanine | 164.0717(14.36),147.0448(43.28),146.5895(1.85),117.0313(0.35),103.0548(23.45),91.0559(3.35),77.0421(2.81),72.011(7.03),69.9991(0.82) |
| 13 | 6.7 | [M+H]+ | 203.13902 | 203.13918 | 0.8 | 3227 | C_9_H_18_N_2_O_3_ | L-Alanyl-L-norleucine | 157.1256(32.78),132.1046(15.03),105.0705(6.7),86.1034(18.2),86.0963(27.3) |
| 14^*^ | 8.04 | [M-H]- | 203.0826 | 203.08345 | 4.2 | 123764 | C_11_H_12_N_2_O_2_ | L-Tryptophan | 203.0824(15.27),186.055(2),159.0922(5.45),142.0661(15.36),130.0666(1.68),117.0534(1.69),116.0505(43.62),115.6906(0.49),74.0259(7.34),72.0097(1.03) |
| 15 | 8.39 | [M+H]+ | 625.17631 | 625.17497 | -2.2 | 30699 | C_28_H_32_O_16_ | Complanatuside | 463.122(17.73),301.0707(67.71),269.0448(3.97),241.0493(2.27) |
| 16 | 8.52 | [M+NH4]+ | 456.18642 | 456.18616 | -0.6 | 1082 | C_21_H_26_O_10_ | Cnidimol 7-glucoside | 123.0442(36.79),107.0494(9.74) |
| 17 | 8.68 | [M+COOH]- | 655.15049 | 655.15007 | -0.6 | 3734 | C_27_H_30_O_16_ | Kaempferol 3-O-sophoroside | 609.1976(2.27),609.1472(30.72),447.0955(22.87),327.0454(3.47),309.0383(4.69),285.043(10.5),285.0317(0.85),284.041(0.84),195.0676(1.4),151.0034(1.54) |
| 18^*^ | 8.71 | [M-H]- | 137.02442 | 137.02503 | 4.5 | 9833 | C_7_H_6_O_3_ | 4-Hydroxybenzoic acid | 137.0235(6.47),93.035(75),92.0317(2.46),65.0423(13.78) |
| 19 | 9.09 | [M-H]- | 579.17193 | 579.17667 | 8.2 | 11979 | C_27_H_32_O_14_ | Liquiritigenin-7,4-diglucoside | 579.224(0.86),579.1844(31.64),417.1294(7.84),417.1061(2.2),401.1165(5.26),373.1455(2.79),255.0702(10.53),254.0824(6.31),239.0685(3.51),149.0255(0.59),135.0086(2.08),133.0594(3.17),119.0512(1.7),118.0495(10.43) |
| 20 | 9.15 | [M+COOH]- | 639.15558 | 639.15593 | 0.5 | 26780 | C_27_H_30_O_15_ | Genistein 7-O-glucosylglucoside | 639.2007(0.57),639.1549(4.61),593.1483(3.26),432.0959(3.22),431.0991(67.79),431.0499(0.57),311.0533(0.7),269.0475(2.46),268.0365(9.63) |
| 21 | 9.5 | [M-H]- | 195.06628 | 195.06643 | 0.8 | 5791 | C_10_H_12_O_4_ | 3,4-Dimethoxyphenylacetic acid | 195.0611(1.65),180.043(6.35),165.06(10.72),151.0381(1.45),150.033(59.4),122.0361(20.41) |
| 22 | 9.59 | [M+H]+ | 377.14556 | 377.14469 | -2.3 | 43153 | C_17_H_20_N_4_O_6_ | (-)-Riboflavin | 377.1464(40.91),359.1512(0.45),359.1356(2.19),243.0871(23.16),226.0572(0.95),200.0814(1.71),198.0661(6.87),197.0887(0.45),172.0864(10.34),170.073(1.62),157.0699(0.76),145.0758(1.32),69.0363(0.36),57.0375(0.52) |
| 23 | 9.85 | [M+H]+ | 166.08626 | 166.08626 | 0.1 | 1035 | C_9_H_11_NO_2_ | L-Phenylalanine isomer | 120.0859(45.6),103.0588(11.99),95.0604(7.68),79.0576(10.51),78.0356(3.48),77.0405(20.74) |
| 24 | 9.91 | [M+H]+ | 463.12349 | 463.12296 | -1.2 | 29705 | C_22_H_22_O_11_ | Rhamnocitin 3-glucoside | 301.0686(48.53),286.0465(1.94),269.0431(7.56),253.0839(3.57),241.0484(5.9),213.0525(4.73),197.0589(1.42),137.0212(1.81) |
| 25^*^ | 11 | [M+COOH]- | 491.1184 | 491.11907 | 1.4 | 2238621 | C_22_H_22_O_10_ | Calycosin-7-glucoside | 491.1196(5.54),284.0657(1.25),283.3967(1.08),283.0597(40.29),268.0365(25.23),267.029(3.1),240.0424(2.68),239.0343(3.48),211.0393(2.54),184.0531(1.05) |
| 26 | 11.14 | [M-H]- | 463.0882 | 463.08747 | -1.6 | 4095 | C_21_H_20_O_12_ | Isoquercitrin | 1.733519(60),0.736011(60),0.293752(60),0.574667(60),0.767776(60),1.421943(90),0.603133(60),0.231297(60) |
| 27 | 11.25 | [M+H]+ | 477.13914 | 477.13819 | -2 | 36567 | C_23_H_24_O_11_ | Astraisoflavan glucoside | 315.0847(66.69),300.0614(13.76),255.0653(1.67),167.0342(1.39) |
| 28^*^ | 11.44 | [M-H]- | 193.05063 | 193.05117 | 2.8 | 14066 | C_10_H_10_O_4_ | Isoferulic acid | 193.0524(11.13),178.0283(13),149.0636(1.03),149.0554(3.66),135.0416(1.52),134.0377(47.35),133.0302(17.24),132.0201(2.91),106.0385(2.17) |
| 29 | 11.75 | [M+H]+ | 433.11292 | 433.11209 | -1.9 | 28930 | C_21_H_20_O_10_ | Genistin | 271.0581(73.02),153.018(3.11) |
| 30 | 12.58 | [M+H]+ | 285.07575 | 285.07567 | -0.3 | 16461 | C_16_H_12_O_5_ | Glycitein | 285.1303(12.79),285.0752(20.04),270.0499(8.16),253.0501(6.14),241.0446(0.42),213.0542(7.9),197.0581(4.68),169.0626(2.81),139.0543(0.42),137.0242(6.55),134.0362(2.08),115.0556(0.87), |
| 31 | 12.75 | [M+H]+ | 419.17004 | 419.16982 | -0.5 | 8134 | C_22_H_26_O_8_ | Syringaresinol | 330.106(6.3),315.0846(6.04),217.0855(6.09),205.0877(5.01),173.061(5.59),167.07(3.22),156.058(2.21),127.0551(1.55),117.0716(1.13),115.0549(1.51) |
| 32 | 12.84 | [M+H]+ | 301.07066 | 301.07073 | 0.2 | 12025 | C_16_H_12_O_6_ | Chrysoeriol | 301.0679(32.82),286.0478(8.13),269.0461(4.53),229.0494(6.69),213.0583(1.88),153.019(3.65),152.0098(3.18),139.0569(1.96),128.0606(2.29),124.0174(2.06) |
| 33 | 12.89 | [M+H]+ | 285.07575 | 285.07542 | -1.2 | 1042 | C_16_H_12_O_5_ | Wogonin | 285.0809(19.98),257.0827(15.18),229.0849(34.03),197.057(13.29),185.0583(6.44),137.0292(11.08) |
| 34^*^ | 14.95 | [M+COOH]- | 475.12349 | 475.12431 | 1.7 | 1093824 | C_22_H_22_O_9_ | Ononin | 475.1227(4.24),268.0702(1.9),267.0653(67.28),252.042(16.42),251.0348(1.98),223.0383(1.84) |
| 35 | 14.99 | [M+H]+ | 269.08084 | 269.08046 | -1.4 | 131609 | C_16_H_12_O_4_ | Formononetin Isomer | 269.0807(24.57),253.0486(7.6),237.0543(6.19),213.0909(3.75),197.0597(10.79),181.0648(3.03) |
| 36 | 15.02 | [M+H]+ | 489.13914 | 489.13752 | -3.3 | 257595 | C_24_H_24_O_11_ | 6''-O-Acetylglycitin | 285.0742(67.63),270.0518(9.03) |
| 37 | 15.11 | [M+COOH]- | 505.13405 | 505.13414 | 0.2 | 14831 | C_23_H_24_O_10_ | 6,4 '- Dimethoxyisoflavone-7-O-glucoside | 505.2745(5.7),505.13(1.43),329.0667(2.09),298.0771(2.08),297.0772(47.52),285.0689(0.37),282.0532(21.41),267.0274(1.85),254.0554(3.71),239.0326(5.48) |
| 38 | 15.23 | [M+H]+ | 595.20213 | 595.20085 | -2.2 | 36121 | C_28_H_34_O_14_ | Poncirin | 531.1803(5.06),247.0932(2.79),233.0818(1.7),209.079(2.23),203.0688(1.88),191.0698(2.57),167.0699(2.55),151.0758(1.04) |
| 39 | 15.48 | [M+H]+ | 301.10705 | 301.10675 | -1 | 486437 | C_17_H_16_O_5_ | Methylnissolin | 301.1053(3.24),269.0776(1.83),167.0695(26.27),152.0466(6.85),151.0381(1.82),147.0431(2.86),134.0366(6.05),123.044(2.83),106.0421(1.79),105.0347(2.63),78.049(1.81) |
| 40^*^ | 15.46 | [M+COOH]- | 507.1497 | 507.15008 | 0.7 | 1211137 | C_23_H_26_O_10_ | Astraisoflavan-7-O-D-glucoside | 507.1512(17.29),461.1449(1.86),299.0924(36.06),284.0687(16.95),269.0451(18.06),241.0503(1.95),207.0505(0.52) |
| 41^*^ | 15.47 | [M+H]+ | 301.10705 | 301.10631 | -2.5 | 486437 | C_17_H_16_O_5_ | 3-hydroxy-9,10-dimethoxy-pterocarpan | 301.1053(3.24),269.0776(1.83),167.0695(26.27),152.0466(6.85),151.0381(1.82),147.0431(2.86),134.0366(6.05),123.044(2.83),106.0421(1.79),105.0347(2.63),78.049(1.81) |
| 42^*^ | 15.63 | [M+H]+ | 465.17552 | 465.17396 | -3.4 | 68922 | C_23_H_28_O_10_ | 7,2'-Dihydroxy-3',4'-dimethoxyisoflavane-7-O-glucoside | 167.0702(33.93),123.045(13.71) |
| 43 | 15.61 | [M-H]- | 301.10815 | 301.10793 | -0.7 | 12466 | C_17_H_18_O_5_ | Isomucronulatol | 301.1072(19.85),300.999(7.26),286.0873(2.89),271.0632(17.61),256.0337(6.08),253.0517(10.03),149.0231(6.77),147.0443(0.69),135.0461(3.11),135.0373(0.66),125.0218(4.67),121.0312(6.92) |
| 44 | 15.63 | [M+H]+ | 465.17552 | 465.17396 | -3.4 | 68922 | C_23_H_28_O_10_ | Astraisoflavanin | 6.912929(503),59.36791(6427),3.343585(431),4.180819(469),3.85567(442),3.435234(405),4.259877(562),23.99259(2565) |
| 45^*^ | 15.74 | [M-H]- | 283.0612 | 283.0614 | 0.7 | 1784795 | C_16_H_12_O_5_ | Calycosin | 283.0595(4.13),268.0357(9.17),267.0295(3.99),240.0408(9.11),239.0333(8.05),211.0383(10.41),195.0442(4.9),184.0514(3.32),183.0443(3.58),148.0157(3.67),135.0078(3.12),91.0193(1.63) |
| 46 | 15.97 | [M+H]+ | 947.52101 | 947.5158 | -5.5 | 72366 | C_47_H_78_O_19_ | AstragalosideV/VI/VII | 947.517(18.9),587.3895(2.29),473.3584(3.04),455.3516(9.03),437.3397(12.53),419.3283(6.46),311.2324(0.47),305.1594(1.16),297.2203(1.04),143.1067(8.65),141.1271(0.65),127.1139(0.58),125.0967(1.59),123.117(0.56) |
| 47 | 15.98 | [M+H]+ | 315.08631 | 315.08562 | -2.2 | 95110 | C_17_H_14_O_6_ | Kumatakenin | 315.0853(25.53),300.0619(13.52),283.0587(2.33),255.0647(2.32),243.0644(3.61),240.0411(2.65),212.0478(2.03),167.0348(2.81) |
| 48 | 16.31 | [M+H]+ | 269.08084 | 269.08047 | -1.4 | 7938 | C_16_H_12_O_4_ | 4'-Hydroxy-5-methoxyflavone | 269.0795(23.03),254.0585(7.82),253.0513(8.76),237.0546(7.57),225.0688(2.55),213.0927(3.03),197.0619(8.23),181.0633(3.56),152.0633(1.16),141.0714(4.26),81.0729(1.87) |
| 49 | 16.29 | [M+COOH]- | 517.13405 | 517.13445 | 0.8 | 134255 | C_24_H_24_O_10_ | 6'-O-acetyl formononetin | 517.1364(10.62),309.0783(0.97),281.0838(0.67),267.0668(64.05),252.0433(17.58),224.0513(0.61),223.0478(0.88),208.0533(0.44) |
| 50^*^ | 16.34 | [M+COOH]- | 829.45801 | 829.45875 | 0.9 | 42881 | C_41_H_68_O_14_ | Isoastragaloside IV | 830.4654(1.56),829.4636(8.47),785.466(0.77),784.4567(28.4),783.5469(0.22),783.4554(53.99),621.4006(0.6),489.3512(0.59),221.0665(0.33),161.0433(0.53),101.0248(0.31),89.0248(0.19) |
| 51 | 16.36 | [M-H]- | 271.0612 | 271.06106 | -0.5 | 2721 | C_15_H_12_O_5_ | Naringenin | 271.148(1.56),271.0599(30.36),211.1282(6.87),209.1499(6.16),187.0392(6.47),177.0231(6.29),151.0028(18.67),135.0409(4.95),119.0474(13.45),107.0142(2.94),65.0016(2.29) |
| 52 | 16.45 | [M+H]+ | 271.0601 | 271.05966 | -1.6 | 5592 | C_15_H_10_O_5_ | Apigenin | 271.0875(3.68),271.0592(38.07),253.0591(1.95),243.0662(4.99),197.0585(3.08),153.0197(5.73),149.0238(1.93),141.0712(4.62),121.0303(3.21),91.056(3.03) |
| 53 | 16.57 | [M+COOH]- | 549.16027 | 549.16042 | 0.3 | 68399 | C_25_H_28_O_11_ | Methylnissolin-acetyl-3-O-glucoside | 550.1766(4.01),549.2467(4.01),549.1937(2),549.1662(40.32),457.1795(2.74),299.095(30.56),284.0696(5.77),284.0517(0.72),269.0492(7.41),269.0412(2.46) |
| 54 | 16.59 | [M+H]+ | 301.10705 | 301.10649 | -1.9 | 11306 | C_17_H_16_O_5_ | Methylnissolin isomer | 301.1392(5.53),301.1066(9.39),286.0459(5.7),241.0835(3.19),167.0685(24.75),164.0462(2.39),152.0458(4.18),151.0381(1.38),134.0391(5.21),123.0416(1.66),78.0484(1.65) |
| 55 | 16.92 | [M+H]+ | 301.07066 | 301.06956 | -3.7 | 39439 | C_16_H_12_O_6_ | Pratensein | 301.0697(18.6),286.0485(5.84),269.0451(5.33),241.0495(6.64),230.059(2),229.0493(12.1),187.037(1.64),155.0486(1.2),153.0182(6.19),152.0126(1.1),134.037(4.22) |
| 56 | 17.14 | [M+COOH]- | 871.46858 | 871.47019 | 1.8 | 14652 | C_43_H_70_O_15_ | Astragaloside II isomer | 872.5018(2.13),872.4699(5.95),872.4412(0.44),871.4707(29.34),826.4711(5.6),826.4351(2.32),825.4645(24.97),783.462(7.44),783.4295(0.29),766.44(3.47),765.4351(10.29) |
| 57^*^ | 17.35 | [M+H]+ | 785.46818 | 785.46456 | -4.6 | 171021 | C_41_H_68_O_14_ | Astragaloside IV | 473.3616(3.97),455.3507(8.13),437.3407(13.04),419.3295(6.71),143.1067(17),125.0968(3.63) |
| 58^*^ | 17.35 | [M+H]+ | 785.46818 | 785.46456 | -4.6 | 171021 | C_41_H_68_O_14_ | Astragaloside III | 437.3407(13.04),143.1067(17) |
| 59^*^ | 17.65 | [M-H]- | 267.06628 | 267.0667 | 1.6 | 1041060 | C_16_H_12_O_4_ | Formononetin | 267.0678(5.76),253.0463(3.14),252.3593(1.27),252.0427(19.5),251.0356(10.87),224.0484(5.33),223.0403(14.35),208.0542(3.29),195.0455(11.28),167.0505(3.93),135.0098(3.08),132.0227(4.95),104.028(1),91.0205(1.98) |
| 60 | 18 | [M+H]+ | 301.10705 | 301.10659 | -1.5 | 470546 | C_17_H_16_O_5_ | 3-Hydoxy-9,10-dimethoxyptercarpan isomer | 167.0699(23.71),152.0472(9),134.0367(7.38),123.0448(3.32),106.0423(2.76),105.0343(2.45),78.0488(2.2) |
| 61^*^ | 18.2 | [M+COOH]- | 987.51592 | 987.51584 | -0.1 | 76553 | C_48_H_78_O_18_ | Soyasaponin I | 942.5194(43.86),941.5883(0.16),941.516(49.99) |
| 62^*^ | 18.25 | [M+COOH]- | 871.46858 | 871.46965 | 1.2 | 1323881 | C_43_H_70_O_15_ | Astragaloside II | 872.4764(27.87),872.0684(1.45),871.4729(41.31),826.4742(2.99),825.4686(11.45),765.4474(4.01),179.0557(0.44),59.0164(0.54) |
| 63^*^ | 18.99 | [M+H]+ | 827.47875 | 827.47513 | -4.4 | 113183 | C_43_H_70_O_15_ | Isoastragaloside | 455.3486(7.89),437.3391(13.58),419.3291(6.3),143.1066(14.69),125.0962(3.38) |
| 64 | 19.45 | [M+H]+ | 285.07575 | 285.07507 | -2.4 | 9900 | C_16_H_12_O_5_ | Wogonin isomer | 285.0753(20.96),270.0524(40.99),242.0526(3.83),213.0538(2.39),168.0057(11.59) |
| 65^*^ | 19.48 | [M+COOH]- | 871.46858 | 871.47035 | 2 | 459433 | C_43_H_70_O_15_ | Cyclocephaloside II | 872.4761(28.47),872.0676(1.35),871.4738(42.65),826.472(3.42),825.469(11.33),765.4464(2.91),59.016(0.38) |
| 66 | 20.27 | [M+H]+ | 869.48931 | 869.48611 | -3.7 | 80908 | C_45_H_72_O_16_ | Isoastragaloside I isomer | 473.3568(2.36),455.3509(5.18),437.3421(8.18),217.071(10.8),157.0497(13.66),143.1073(13.95),125.0976(3.42),97.0308(1.45) |
| 67^*^ | 20.92 | [M+COOH]- | 913.47914 | 913.48038 | 1.4 | 364670 | C_45_H_72_O_16_ | Isoastragaloside I | 914.4859(29.03),914.092(0.58),913.4828(50.34),868.484(1.89),867.4798(7.85),825.4624(1.94),59.0155(0.39) |
| 68^*^ | 21.85 | [M+COOH]- | 913.47914 | 913.48059 | 1.6 | 173608 | C_45_H_72_O_16_ | Astragaloside I | 914.4867(28.45),913.4855(57.18),867.4778(5.61),826.4762(0.18),59.0166(0.26) |
| 69 | 23.43 | [M+H]+ | 911.49988 | 911.49574 | -4.5 | 7289 | C_47_H_74_O_17_ | Acetytastragaloside | 713.4186(7.02),419.331(6.49),259.0823(7.84),199.0595(6.49),184.0749(2.94),157.0505(3.13),143.1088(11.24),139.0398(7.49),125.0978(2.34),97.0303(2.53) |

* Identified by comparison with reference standards.

## Table S3 Changes in physiological functions, biochemical functions, immune cells, and immune cytokines between Group d0 and Group d15. (**p*<0.05, ***p*<0.01, ****p*<0.001)

| Variables | Group d0 | Group d15 | *p*-value |
| --- | --- | --- | --- |
| SBP | 116.7±8.683 | 118.1±8.470 | 0.5586 |
| DBP | 72.15±7.450 | 75.10±8.026 | 0.2357 |
| WBC | 6.273±1.7 | 6.174±1.34 | 0.7008 |
| RBC | 4.802±0.5489 | 4.811±0.5256 | 0.8171 |
| HGB | 143.3±14.21 | 143.1±14.05 | 0.7868 |
| PLT | 265.2±48.69 | 272.9±46.91 | 0.1335 |
| NEUT | 56.64±8.558 | 55.03±9.404 | 0.1490 |
| LYM | 33.36±7.575 | 35.29±8.305 | 0.0694 |
| AST | 17.55±4.454 | 17.8±3.888 | 0.7807 |
| ALT | 19.55±11.95 | 17.5±10.14 | 0.4678 |
| BUN | 4.916±1.168 | 4.738±1.221 | 0.3799 |
| Scr | 71.87±11.79 | 68.97±11.83^**^ | 0.0051 |
| CD3+ | 68.39±8.363 | 70.65±8.612 | 0.1640 |
| CD3+CD4+CD8- | 34.49±4.371 | 35.5±4.712 | 0.4786 |
| CD3+CD4-CD8+ | 28.77±7.706 | 30.47±7.38 | 0.3684 |
| CD19+ | 13.14±3.992 | 12.51±3.554 | 0.4292 |
| CD3-CD16+CD56+ | 13.77±7.326 | 13.05±6.881 | 0.6328 |
| IL-2 | 1.594±0.3685 | 1.288±0.5083^*^ | 0.0175 |
| IL-6 | 0.397±0.8195 | 3.183±5.496^*^ | 0.0199 |
| IL-10 | 1.27±0.1954 | 1.333±0.383 | 0.3947 |
| IFN-γ | 1.51±1.407 | 3.339±3.831^*^ | 0.0255 |
| IL-17 | 1.654±1.158 | 1.999±1.912 | 0.1251 |
| IL-4 | 2.139±2.262 | 2.666±2.596^*^ | 0.0419 |
| IL-12P70 | 1.561±2.274 | 2.459±3.459^*^ | 0.045 |
| TNF-α | 3.325±6.903 | 6.717±7.064^***^ | 0.0004 |

## Table S4 Reliability of analytical methods based on the RSD% values of the RT, *m/z*, and Intensity by UHPLC-Q-TOF/MS.

| Features | Ion mode | | RT (min) | | RT RSD (%) | | *m/z* (Da) | *m/z* RSD(%) | Intensity | Intensity RSD (%) |
| --- | --- | --- | --- | --- | --- | --- | --- | --- | --- | --- |
| Serum lipidomics analysis | | | | | | | | | | |
| F1 | | [M+H]+ | | 0.99 | | 0.45 | 376.26 | <0.001 | 161895.80 | 6.41 |
| F2 | | [M+H]+ | | 3.23 | | 0.26 | 358.37 | <0.001 | 41548.60 | 26.02 |
| F3 | | [M+H]+ | | 4.64 | | 0.30 | 675.68 | <0.001 | 196209.00 | 24.92 |
| F4 | | [M+H]+ | | 7.66 | | 0.46 | 811.67 | <0.001 | 107207.20 | 14.83 |
| F5 | | [M+H]+ | | 10.57 | | 0.29 | 533.53 | <0.001 | 17492.80 | 16.48 |
| F6 | | [M-H]- | | 1.76 | | 0.31 | 564.33 | <0.001 | 50969.33 | 13.85 |
| F7 | | [M-H]- | | 3.81 | | 0.11 | 281.25 | <0.001 | 140203.67 | 9.36 |
| F8 | | [M-H]- | | 5.85 | | 0.11 | 747.57 | <0.001 | 113489.33 | 6.81 |
| F9 | | [M-H]- | | 6.18 | | 0.13 | 802.56 | <0.001 | 278478.67 | 13.97 |
| F10 | | [M-H]- | | 8.44 | | 0.17 | 845.68 | <0.001 | 11512.83 | 19.87 |
| Serum metabolomics analysis | | | | | | | | | | |
| F1 | [M+H]+ | | 0.99 | | 0.31 | | 338.34 | <0.001 | 5940552.60 | 5.98 |
| F2 | [M+H]+ | | 2.26 | | 0.37 | | 146.08 | <0.001 | 26554.60 | 5.71 |
| F3 | [M+H]+ | | 5.87 | | 0.39 | | 160.13 | <0.001 | 17187.80 | 6.55 |
| F4 | [M+H]+ | | 6.58 | | 0.17 | | 144.10 | <0.001 | 207732.60 | 6.56 |
| F5 | [M+H]+ | | 8.29 | | 0.07 | | 130.05 | <0.001 | 78140.60 | 3.02 |
| F6 | [M-H]- | | 1.33 | | 0.44 | | 238.08 | <0.001 | 183259.75 | 14.86 |
| F7 | [M-H]- | | 2.26 | | 0.37 | | 146.08 | <0.001 | 26554.60 | 5.71 |
| F8 | [M-H]- | | 4.44 | | 0 | | 802.56 | <0.001 | 29092.00 | 11.17 |
| F9 | [M-H]- | | 6.25 | | 0.09 | | 414.18 | <0.001 | 167166.25 | 9.31 |
| F10 | [M-H]- | | 8.49 | | 0.06 | | 145.06 | <0.001 | 119471.00 | 6.39 |
| Urine metabolomics analysis | | | | | | | | | | |
| F1 | [M+H]+ | | 1.96 | | 0.47 | | 146.09 | <0.001 | 262709.89 | 18.10 |
| F2 | [M+H]+ | | 7.27 | | 0.59 | | 151.06 | <0.001 | 111487.33 | 20.17 |
| F3 | [M+H]+ | | 12.84 | | 0.26 | | 265.12 | <0.001 | 2651161.44 | 17.18 |
| F4 | [M+H]+ | | 16.84 | | 0.10 | | 432.28 | <0.001 | 309187.67 | 15.23 |
| F5 | [M+H]+ | | 19.09 | | 0.11 | | 520.33 | <0.001 | 392926.67 | 14.06 |
| F6 | [M-H]- | | 1.56 | | 0.21 | | 387.12 | <0.001 | 50969.33 | 13.85 |
| F7 | [M-H]- | | 2.81 | | 0.18 | | 281.25 | <0.001 | 140203.67 | 9.36 |
| F8 | [M-H]- | | 5.85 | | 0.11 | | 747.57 | <0.001 | 113489.33 | 6.81 |
| F9 | [M-H]- | | 6.18 | | 0.13 | | 802.56 | <0.001 | 278478.67 | 13.97 |
| F10 | [M-H]- | | 8.44 | | 0.17 | | 845.68 | <0.001 | 11512.83 | 19.87 |
| Feces lipidomics analysis | | | | | | | | | | |
| F1 | [M+H]+ | | 0.99 | | 0 | | 376.26 | <0.001 | 69250.80 | 7.55 |
| F2 | [M+H]+ | | 2.69 | | 0 | | 324.29 | <0.001 | 115388.80 | 11.76 |
| F3 | [M+H]+ | | 4.66 | | 0.09 | | 338.34 | <0.001 | 2788772.00 | 8.07 |
| F4 | [M+H]+ | | 8.43 | | 0.06 | | 758.22 | <0.001 | 163612.80 | 12.99 |
| F5 | [M+H]+ | | 11.29 | | 0.04 | | 896.77 | <0.001 | 933483.80 | 11.43 |
| F6 | [M-H]- | | 0.61 | | 0.73 | | 160.13 | <0.001 | 21612.20 | 12.97 |
| F7 | [M-H]- | | 3.42 | | 0.13 | | 485.36 | <0.001 | 60480.40 | 8.51 |
| F8 | [M-H]- | | 5.87 | | 0 | | 385.35 | <0.001 | 1015053.00 | 12.62 |
| F9 | [M-H]- | | 8.43 | | 0.06 | | 758.22 | <0.001 | 163612.80 | 12.99 |
| F10 | [M-H]- | | 12.19 | | 0.04 | | 824.77 | <0.001 | 295802.40 | 7.96 |
| Feces metabolomics analysis | | | | | | | | | | |
| F1 | [M+H]+ | | 1.29 | | 0.69 | | 663.45 | <0.001 | 188549.00 | 8.38 |
| F2 | [M+H]+ | | 2.84 | | 0.31 | | 143.07 | <0.001 | 118980.20 | 22.86 |
| F3 | [M+H]+ | | 5.81 | | 0.26 | | 137.05 | <0.001 | 197735.20 | 24.92 |
| F4 | [M+H]+ | | 7.45 | | 0.48 | | 380.22 | <0.001 | 377784.2 | 21.58 |
| F5 | [M+H]+ | | 8.28 | | 0.41 | | 165.13 | <0.001 | 1526246.00 | 8.05 |
| F6 | [M-H]- | | 0.94 | | 0.58 | | 467.30 | <0.001 | 330654.80 | 6.41 |
| F7 | [M-H]- | | 2.84 | | 0.31 | | 143.07 | <0.001 | 118980.20 | 14.22 |
| F8 | [M-H]- | | 4.65 | | 0.18 | | 135.03 | <0.001 | 231870.40 | 8.03 |
| F9 | [M-H]- | | 7.32 | | 0.11 | | 180.06 | <0.001 | 53589.40 | 6.08 |
| F10 | [M-H]- | | 9.58 | | 0.14 | | 96.96 | <0.001 | 71516.00 | 14.62 |

## Table S5 Changes in the number of features during data processing.

|  | Data processing | Lipidome | pos | neg | Metabolome | pos | neg |
| --- | --- | --- | --- | --- | --- | --- | --- |
| Serum | MS-DAIL | 1322 | 798 | 524 | 1826 | 762 | 1064 |
|  | MS-FLO | 1166 | 674 | 492 | 1752 | 715 | 1037 |
|  | SMFs (FC>1.2 or <0.83, *p*<0.05) | 209 | 156 | 53 | 115 | 66 | 49 |
|  | PBs (Positive and negative ions combined) | 51 |  |  | 38 |  | |
| Urine | MS-DAIL | - | - | - | 1826 | 762 | 1064 |
|  | MS-FLO | - | - | - | 1752 | 715 | 1037 |
|  | SMFs (FC>1.2 or <0.83, *p*<0.05) | - | - | - | 113 | 49 | 64 |
|  | PBs (Positive and negative ions combined) | - |  |  | 41 |  | |
| Feces | MS-DAIL | 2295 | 1189 | 1106 | 2725 | 1087 | 1638 |
|  | MS-FLO | 1968 | 977 | 991 | 2059 | 1022 | 1037 |
|  | SMFs (FC>1.2 or <0.83, *p*<0.05) | 198 | 92 | 106 | 82 | 32 | 50 |
|  | PBs (Positive and negative ions combined) | 64 |  |  | 40 |  | |

## Table S6 The list of 89 immunomodulation PBs of AROL in serum.

| No. | RT  (min) | *m/z* (Da) | Ion Type | Formula | Identity | Pubchem CID | HMDB ID | Trend | *p*- value | FC  d15/d0 |
| --- | --- | --- | --- | --- | --- | --- | --- | --- | --- | --- |
| **Lipidomics analysis** | | | | | | | | | | |
| PB1 | 0.64 | 239.2014 | [M-H]- | C_15_H_28_O_2_ | Pentadecenoic acid | 53887649 | HMDB0302623 | ↑ | 0.002 | 4.22 |
| PB2 | 0.68 | 321.2437 | [M-H]- | C_20_H_34_O_3_ | 8-HETrE | 35025143 | HMDB0060052 | ↑ | 0.014 | 1.55 |
| PB3 | 1.03 | 440.2287 | [M+NO_3_]- | C_22_H_34_O_5_ | 2-(3,5-Ditert-butyl-4-hydroxy benzyl) malonic acid diethyl ester | 4407717 | HMDB0248901 | ↑ | 0.044 | 0.75 |
| PB4 | 1.05 | 309.1742 | [M-H]- | C_17_H_34_O_4_ | MG (i-14:0/0:0/0:0) | 51693118 | HMDB0072867 | ↑ | 0.009 | 1.65 |
| PB5 | 1.13 | 421.2276 | [M-H]- | C_20_H_38_O_7_S | Dioctyl sulfosuccinate | 11339 | HMDB0251779 | ↑ | 0.001 | 2.53 |
| PB6 | 1.14 | 317.2168 | [M-H]- | C_20_H_30_O_3_ | 12-HEPE | 10041593 | HMDB0010202 | ↑ | 0.004 | 1.92 |
| PB7 | 1.16 | 272.2586 | [M+H]+ | C_16_H_33_NO_2_ | Hexadecasphingosine | 8890 | HMDB0242181 | ↑ | 0.008 | 2.74 |
| PB8 | 1.53 | 302.3052 | [M+H]+ | C_18_H_39_NO_2_ | Sphinganine | 91486 | HMDB0000269 | ↑ | 0.007 | 3.11 |
| PB9 | 2.54 | 400.3777 | [M+H]+ | C_24_H_49_NO_3_ | TG (14:0/20:0/20:4(8Z,11Z,14Z,17Z)) | 131753348 | HMDB0042206 | ↑ | 0.010 | 0.74 |
| PB10 | 2.54 | 356.3518 | [M+H]+ | C_22_H_45_NO_2_ | Arachidoyl Ethanolamide | 3787294 | HMDB0248559 | ↑ | 0.006 | 2.93 |
| PB11 | 3.02 | 550.4452 | [M+H]+ | C_33_H_59_NO_5_ | 15-(3,4-Dimethyl-5-pentylfuran-2-yl) pentadecenoylcarnitine | 156962899 | HMDB0241841 | ↑ | 0.025 | 2.21 |
| PB12 | 3.69 | 244.1894 | [M+H]+ | C_13_H_25_NO_3_ | N-Undecanoylglycine | 454092 | HMDB0013286 | ↓ | 0.017 | 1.57 |
| PB13 | 4.42 | 283.2642 | [M-H]- | C_18_H_36_O_2_ | 7-methyl heptadecanoic acid | [15609](https://pubchem.ncbi.nlm.nih.gov/compound/15609) | HMDB0340358 | ↓ | 0.001 | 4.01 |
| PB14 | 4.68 | 913.5868 | [M-H]- | C_49_H_87_O_13_P | PI (18:2(9Z,12Z)/22:2(13Z,16Z)) | 52927785 | HMDB0009856 | ↑ | 0.025 | 1.26 |
| PB15 | 4.88 | 484.4717 | [M+H]+ | C_30_H_61_NO_3_ | Cer (d18:0/12:0) | 53481046 | HMDB0011758 | ↑ | 0.013 | 2.08 |
| PB16 | 5.06 | 859.5361 | [M-H]- | C_45_H_81_O_13_P | PI (18:0/18:3(6Z,9Z,12Z)) | 52927736 | HMDB0009810 | ↑ | 0.011 | 1.98 |
| PB17 | 5.20 | 711.4937 | [M+Na]+ | C_45_H_68_O_5_ | DG (20:4n3/0:0/22:6n3) | 131802071 | HMDB56380 | ↑ | 0.008 | 2.07 |
| PB18 | 5.20 | 653.4386 | [M+H]+ | C_33_H_65_O_10_P | PG (i-14:0/i-13:0) | 131823327 | HMDB0116694 | ↑ | 0.009 | 1.44 |
| PB19 | 5.21 | 556.5277 | [M+NH_4_]+ | C_34_H_66_O_4_ | FAHFA (16:0/12-O-18:0) | 86290202 | HMDB0112113 | ↑ | 0.015 | 2.12 |
| PB20 | 5.24 | 824.5537 | [M+HCOO]- | C_44_H_78_NO_8_P | PC (14:0/22:5(4Z,7Z,10Z,13Z,16Z)) | 53479341 | HMDB0007890 | ↑ | 0.018 | 1.39 |
| PB21 | 5.37 | 340.357 | [M+H]+ | C_22_H_45_NO | Docosanamide | 76468 | HMDB0000583 | ↓ | 0.005 | 3.38 |
| PB22 | 5.64 | 512.5024 | [M+H]+ | C_32_H_65_NO_3_ | Cer (d18:0/14:0) | 10255824 | HMDB0011759 | ↑ | 0.006 | 1.48 |
| PB23 | 5.71 | 826.5589 | [M+HCOO]- | C_44_H_80_NO_8_P | PC (16:0/20:4(5Z,8Z,11Z,14Z)) | 10747814 | HMDB08014 | ↑ | 0.042 | 1.25 |
| PB24 | 5.91 | 739.5237 | [M+Na]+ | C_47_H_72_O_5_ | DG (22:5n6/0:0/22:5n6) | 131766824 | HMDB0056359 | ↑ | 0.011 | 1.53 |
| PB25 | 5.91 | 681.4717 | [M+H]+ | C_35_H_69_O_10_P | PG (i-12:0/a-17:0) | 131823290 | HMDB0116657 | ↑ | 0.028 | 1.35 |
| PB26 | 5.93 | 584.5615 | [M+NH4]+ | C_36_H_70_O_4_ | FAHFA (18:0/10-O-18:0) | 101527491 | HMDB0112131 | ↑ | 0.017 | 1.66 |
| PB27 | 6.50 | 540.5338 | [M+H]+ | C_34_H_69_NO_3_ | Cer (d18:0/16:0) | 5283572 | HMDB0011760 | ↑ | 0.020 | 2.13 |
| PB28 | 7.15 | 612.5947 | [M+NH4]+ | C_38_H_74_O_4_ | Ethylene glycol distearate | 61174 | HMDB32260 | ↑ | 0.015 | 1.93 |
| PB29 | 7.37 | 568.5652 | [M+H]+ | C_36_H_73_NO_3_ | Cer (d18:0/18:0) | 5283573 | HMDB0011761 | ↑ | 0.009 | 0.75 |
| PB30 | 7.94 | 636.5546 | [M+NH4]+ | C_39_H_70_O_5_ | DG (18:1(9Z)/18:2(9Z,12Z)/0:0) | 9543722 | HMDB0007219 | ↑ | 0.020 | 1.22 |
| PB31 | 8.24 | 596.5961 | [M+H]+ | C_38_H_77_NO_3_ | Cer (d18:0/20:0) | 5283574 | HMDB0011764 | ↑ | 0.018 | 2.15 |
| PB32 | 8.85 | 685.5426 | [M-H]- | C_43_H_74_O_6_ | DG (20:5(5Z,8Z,11Z,14Z,16E)-OH (18R)/0:0/20:0) | 157004460 | HMDB0296354 | ↑ | 0.036 | 1.33 |
| PB33 | 8.86 | 124.9921 | [M-H]- | C_2_H_6_O_4_S | ethyl sulfate | 57448826 | HMDB0031233 | ↑ | 0.024 | 2.01 |
| PB34 | 9.09 | 656.6176 | [M+H]+ | C_40_H_81_NO_5_ | N-(2R-Hydroxydocosanoyl)-2S-amino-1,3S,4R-octadecanetriol | 25201875 | HMDB0035469 | ↑ | 0.029 | 1.67 |
| PB35 | 9.72 | 670.6282 | [M+H]+ | C_41_H_83_NO_5_ | N-(2R-Hydroxytricosanoyl)-2S-amino-1,3S,4R-octadecanetriol | 25200791 | HMDB0035470 | ↑ | 0.021 | 1.71 |
| PB36 | 10.93 | 894.7538 | [M+NH4]+ | C_57_H_96_O_6_ | TG (16:1(9Z)/18:2(9Z,12Z)/20:4(5Z,8Z,11Z,14Z)) | 9544361 | HMDB0005447 | ↑ | 0.022 | 1.28 |
| PB37 | 11.29 | 870.7516 | [M+NH4]+ | C_55_H_96_O_6_ | TG (16:1(9Z)/18:2(9Z,12Z)/18:2(9Z,12Z) | 25240366 | HMDB0005446 | ↑ | 0.001 | 1.40 |
| PB38 | 11.30 | 896.7691 | [M+NH4]+ | C_57_H_98_O_6_ | TG (18:2(9Z,12Z)/18:2(9Z,12Z)/18:2(9Z,12Z)) | 5322095 | HMDB0005474 | ↑ | 0.033 | 1.23 |
| PB39 | 11.65 | 872.769 | [M+NH4]+ | [C_55_H_98_O_6_](https://pubchem.ncbi.nlm.nih.gov/#query=C55H98O6) | TG (14:0/18:1(11Z)/20:3(5Z,8Z,11Z)) | 9544107 | HMDB0042343 | ↑ | 0.009 | 1.30 |
| PB40 | 11.75 | 808.7428 | [M+NH4]+ | C_50_H_94_O_6_ | TG (14:0/15:0/18:1(11Z)) | 131753246 | HMDB0042100 | ↑ | 0.014 | 1.22 |
| PB41 | 11.79 | 834.7513 | [M+NH4]+ | C_52_H_96_O_6_ | TG (15:0/16:1(9Z)/18:1(11Z)) | 165285363 | HMDB0043200 | ↑ | 0.024 | 1.49 |
| PB42 | 11.81 | 860.765 | [M+NH4]+ | C_54_H_98_O_6_ | TG (15:0/18:0/18:3(6Z,9Z,12Z)) | 131754192 | HMDB0043062 | ↑ | 0.014 | 1.30 |
| PB43 | 11.84 | 886.7809 | [M+NH4]+ | C_56_H_100_O_6_ | TG (15:0/18:1(9Z)/20:3n6) | 9544152 | HMDB0043267 | ↑ | 0.007 | 1.62 |
| PB44 | 12.03 | 900.8007 | [M+NH4]+ | C_57_H_102_O_6_ | TG (54:4) | 9544255 | HMDB0258949 | ↑ | 0.020 | 1.28 |
| PB45 | 12.08 | 810.7505 | [M+NH4]+ | C_50_H_96_O_6_ | TG (14:0/15:0/18:0) | 131753240 | HMDB0042094 | ↑ | 0.019 | 1.53 |
| PB46 | 12.10 | 836.7655 | [M+NH4]+ | C_52_H_98_O_6_ | TG (15:0/18:1(11Z)/16:0) | 9543991 | HMDB0043222 | ↑ | 0.033 | 1.23 |
| PB47 | 12.13 | 888.7976 | [M+NH4]+ | C_56_H_102_O_6_ | TG (15:0/18:0/20:3n6) | 9544126 | HMDB0043064 | ↑ | 0.023 | 1.32 |
| PB48 | 12.29 | 890.8129 | [M+NH4]+ | C_56_H_104_O_6_ | TG (15:0/18:1(9Z)/20:1(11Z)) | 9544102 | HMDB0043260 | ↑ | 0.007 | 1.32 |
| PB49 | 12.34 | 852.8006 | [M+NH4]+ | C_53_H_102_O_6_ | TG (15:0/20:0/15:0) | 131754205 | HMDB0043076 | ↑ | 0.005 | 1.70 |
| PB50 | 12.35 | 904.8283 | [M+NH4]+ | C_57_H_106_O_6_ | TG (18:0/18:1(9Z)/18:1(9Z)) | 42627601 | HMDB0005403 | ↑ | 0.014 | 1.28 |
| PB51 | 12.35 | 930.8467 | [M+NH4]+ | C_59_H_108_O_6_ | TG (18:1(9Z)/18:2(9Z,12Z)/20:0) | 131750410 | HMDB0005459 | ↑ | 0.001 | 1.27 |
| **Metabolomics analysis** | | | | | | | | | | |
| PB1 | 0.93 | 544.4562 | [M+NH4]+ | C_31_H_58_O_6_ | TG (12:0/8:0/8:0) | 45278891 | HMDB0094820 | ↑ | 0.047 | 1.29 |
| PB2 | 0.94 | 745.488 | [M-H]- | C_43_H_71_O_8_P | PA (18:2(9Z,12Z)/22:5(7Z,10Z,13Z,16Z,19Z)) | 131821941 | HMDB0114969 | ↓ | 0.001 | 0.57 |
| PB3 | 0.97 | 1287.845 | [M-H]- | C_69_H_126_O_17_P_2_ | CL (14:0/14:0/14:0/18:4(6Z,9Z,12Z,15Z)) | 1[31792930](https://pubchem.ncbi.nlm.nih.gov/compound/131792930) | HMDB0304967 | ↓ | 0.034 | 0.58 |
| PB4 | 0.99 | 432.2767 | [M-H]- | C_25_H_39_NO_5_ | N-Arachidonoyl Glutamic acid | 52922057 | HMDB0241995 | ↓ | 0.005 | 0.69 |
| PB5 | 0.99 | 381.3758 | [M-H]- | C_25_H_50_O_2_ | Pentacosanoic acid | 10468 | HMDB0002361 | ↑ | 0.002 | 1.46 |
| PB6 | 1.00 | 395.3891 | [M-H]- | C_26_H_52_O_2_ | Hexacosanoic acid | 10469 | HMDB0002356 | ↑ | 0.031 | 1.35 |
| PB7 | 1.00 | 414.2141 | [M+NH_4_]+ | C_20_H_28_O_8_ | 4,5-Dihydroniveusin A | 45783165 | HMDB0032844 | ↑ | 0.021 | 1.27 |
| PB8 | 1.01 | 338.2035 | [M+H]+ | C_22_H_27_NO_2_ | Inflatine | 101616 | HMDB0254137 | ↑ | 0.048 | 1.42 |
| PB9 | 1.01 | 133.101 | [M+H]+ | C_5_H_12_N_2_O_2_ | Ornithine | 6262 | HMDB0000214 | ↑ | 0.041 | 1.36 |
| PB10 | 1.01 | 189.1145 | [M+H]+ | C_9_H_16_O_4_ | Azelaic acid | 19347555 | HMDB0000784 | ↑ | 0.040 | 1.42 |
| PB11 | 1.02 | 269.2491 | [M-H]- | C_17_H_34_O_2_ | Heptadecanoic acid | 10465 | HMDB0002259 | ↑ | 0.015 | 1.25 |
| PB12 | 1.03 | 303.2324 | [M-H]- | C_20_H_32_O_2_ | Cis-8,11,14,17-Eicosatetraenoic acid | 11722594 | HMDB0002177 | ↓ | 0.008 | 0.76 |
| PB13 | 1.03 | 325.2367 | [M+H]+ | C_19_H_32_O_4_ | 1-Acetoxy-2-hydroxy-16-heptadecyn-4-one | 115175 | HMDB0031007 | ↓ | 0.020 | 0.63 |
| PB14 | 1.03 | 267.233 | [M-H]- | C_17_H_32_O_2_ | 10Z-Heptadecenoic acid | 5312435 | HMDB0060038 | ↑ | 0.001 | 1.79 |
| PB15 | 1.06 | 475.3768 | [M+H]+ | C_30_H_50_O_4_ | 20-Epi-Eldecalcitol | 19283 | HMDB0245562 | ↑ | 0.015 | 1.27 |
| PB16 | 1.06 | 565.4098 | [M+H]+ | C_40_H_52_O_2_ | E,e-Carotene-3,3'-dione | 6443740 | HMDB0002193 | ↑ | 0.014 | 1.24 |
| PB17 | 1.07 | 447.346 | [M+H]+ | C_28_H_46_O_4_ | Didecyl phthalate | 33599 | HMDB0251217 | ↑ | 0.002 | 1.25 |
| PB18 | 1.08 | 112.9869 | [M-H]- | C_4_H_2_O_4_ | Acetylenedicarboxylic acid | 371 | HMDB0247933 | ↓ | 0.005 | 0.82 |
| PB19 | 1.11 | 702.5386 | [M+H]+ | C_39_H_76_NO_7_P | PEP-16:0/18:1(11Z)) | 53480845 | HMDB0011341 | ↓ | 0.017 | 0.73 |
| PB20 | 1.11 | 709.4887 | [M-H]- | C_40_H_71_O_8_P | PA (15:0/22:4(7Z,10Z,13Z,16Z)) | 52928750 | HMDB0114827 | ↓ | 0.017 | 0.70 |
| PB21 | 1.31 | 311.2067 | [M+H]+ | C_20_H_26_N_2_O | (-)-Ibogaine | 14151360 | HMDB0242213 | ↑ | 0.044 | 1.32 |
| PB22 | 1.33 | 416.2845 | [M+H]+ | C_22_H_41_NO_4_S | Salmeterol | 5152 | HMDB0015073 | ↑ | 0.039 | 1.27 |
| PB23 | 1.40 | 206.1384 | [M+H]+ | C_9_H_19_NO_4_ | Pantothenol | 131204 | HMDB0004231 | ↓ | 0.001 | 0.65 |
| PB24 | 2.74 | 139.0508 | [M+H]+ | C_6_H_6_N_2_O_2_ | Urocanic acid | 736715 | HMDB0000301 | ↓ | 0.013 | 0.68 |
| PB25 | 3.03 | 104.0712 | [M+H]+ | C_4_H_9_NO_2_ | Aminobutyric acid | 119 | HMDB0000112 | ↑ | 0.009 | 1.62 |
| PB26 | 3.94 | 310.2005 | [M+Na]+ | C_15_H_29_NO_4_ | L-Octanoylcarnitine | 11953814 | HMDB0000791 | ↑ | 0.005 | 1.31 |
| PB27 | 4.21 | 286.2005 | [M+H]+ | C_15_H_27_NO_4_ | Octenoyl-L-carnitine | 129692230 | HMDB0240723 | ↑ | 0.014 | 1.33 |
| PB28 | 4.45 | 114.0668 | [M+H]+ | C_4_H_7_N_3_O | Creatinine | 588 | HMDB0000562 | ↑ | 0.003 | 2.19 |
| PB29 | 4.59 | 747.5644 | [M+HCOO]- | C_39_H_79_N_2_O_6_P | SM (d18:1/16:0) | 9939941 | HMDB0010169 | ↑ | 0.050 | 1.31 |
| PB30 | 5.63 | 137.0465 | [M+H]+ | C_5_H_4_N_4_O | Hypoxanthine | 790 | HMDB0000157 | ↑ | 0.000 | 1.73 |
| PB31 | 5.82 | 267.0732 | [M-H]- | C_10_H_12_N_4_O_5_ | Inosine | 6021 | HMDB0000195 | ↑ | 0.005 | 1.89 |
| PB32 | 6.63 | 235.165 | [2M+H]+ | C_5_H_11_NO_2_ | Betaine | 247 | HMDB0000043 | ↑ | 0.000 | 1.35 |
| PB33 | 7.11 | 116.0708 | [M+H]+ | C_5_H_9_NO_2_ | L-Proline | 145742 | HMDB0000162 | ↑ | 0.047 | 1.65 |
| PB34 | 7.14 | 131.0823 | [M+H]+ | C_6_H_10_O_3_ | Ketoleucine | 70 | HMDB0000695 | ↑ | 0.006 | 2.01 |
| PB35 | 7.20 | 179.0794 | [M+H]+ | C_14_H_10_ | Phenanthrene | 995 | HMDB0256390 | ↑ | 0.003 | 1.58 |
| PB36 | 7.20 | 343.2097 | [M+H]+ | C_14_H_26_N_6_O_4_ | PC (16:1(9Z)/20:3(8Z,11Z,14Z)) | 14236207 | HMDB0008014 | ↑ | 0.012 | 1.78 |
| PB37 | 8.61 | 167.9971 | [M-H]- | C_3_H_7_NO_5_S | Cysteic acid | 371 | HMDB0002757 | ↑ | 0.013 | 1.29 |
| PB38 | 9.10 | 174.1239 | [M+H]+ | C_7_H_15_N_3_O_2_ | 2,7-Diamino-7-iminoheptanoic acid | 107984 | HMDB0245671 | ↑ | 0.010 | 1.83 |

## Table S7 The list of 41 immunomodulation PBs of AROL in urine.

| No. | RT  (min) | *m/z*(Da) | Ion Type | Formula | Identity | Pubchem CID | HMDB ID | Trend | *p*-value | FC  d15/d0 |
| --- | --- | --- | --- | --- | --- | --- | --- | --- | --- | --- |
| **Metabolomics analysis** | | | | | | | | | | |
| PB1 | 1.00 | 408.7797 | [M-H]- | C_12_H_3_Cl_7_O | 4-Hydroxy-2,2',3,4',5,5',6-heptachlorobiphenyl | 178007 | HMDB0246441 | ↓ | 0.037 | 0.79 |
| PB2 | 1.19 | 335.0489 | [2M-H]- | C_5_H_4_N_4_O_3_ | Uric acid | 1175 | HMDB0000289 | ↓ | 0.032 | 0.82 |
| PB3 | 1.19 | 210.0844 | [M-H]- | C_9_H_13_N_3_O_3_ | Zalcitabine | 24066 | HMDB0015078 | ↓ | 0.018 | 0.80 |
| PB4 | 1.23 | 123.0565 | [M+H]+ | C_6_H_6_N_2_O | Nicotinamide | 936 | HMDB0001406 | ↓ | 0.018 | 0.59 |
| PB5 | 1.81 | 177.0983 | [M+H]+ | C_5_H_12_N_4_O_3_ | Canavanine | 439202 | HMDB0002706 | ↑ | 0.000 | 31.55 |
| PB6 | 1.88 | 148.0454 | [M-H]- | C_5_H_11_NO_2_S | L-Methionine | 6137 | HMDB0000696 | ↓ | 0.048 | 0.80 |
| PB7 | 1.89 | 142.0868 | [M+H]+ | C_7_H_11_NO_2_ | Ethosuximide | 3291 | HMDB0014731 | ↓ | 0.047 | 0.65 |
| PB8 | 2.03 | 215.1392 | [M+H]+ | C_10_H_18_N_2_O_3_ | Pro-Val | 142984 | HMDB0029030 | ↓ | 0.046 | 0.67 |
| PB9 | 2.27 | 261.0071 | [M-H]- | C_9_H_10_O_7_S | Homovanillic acid sulfate | 29981063 | HMDB0011719 | ↓ | 0.043 | 0.34 |
| PB10 | 2.28 | 173.1087 | [M+H]+ | C_6_H_14_N_4_O_2_ | L-Arginine | 28782 | HMDB0000517 | ↑ | 0.007 | 2.78 |
| PB11 | 2.39 | 169.0136 | [M-H]- | C_7_H_6_O_5_ | 1,2,3-Trihydroxybenzene | 1057 | HMDB0013674 | ↓ | 0.018 | 0.50 |
| PB12 | 4.91 | 153.0661 | [M+H]+ | C_7_H_8_N_2_O_2_ | N1-Methyl-2-pyridone-5-carboxamide | 69698 | HMDB0004193 | ↓ | 0.046 | 0.73 |
| PB13 | 5.73 | 262.1646 | [M+H]+ | C_12_H_23_NO_5_ | hydroxyisovaleroyl carnitine | 57357187 | HMDB0062555 | ↓ | 0.038 | 0.77 |
| PB14 | 5.95 | 206.9963 | [M-H]- | C_11_H_12_O_4_ | (R)-2-Benzylsuccinic acid | 446168 | HMDB0012127 | ↑ | 0.012 | 2.78 |
| PB15 | 6.73 | 132.0459 | [M-H]- | C_8_H_7_NO | 4-Hydroxybenzeneacetonitrile | 26548 | HMDB0029757 | ↓ | 0.014 | 0.32 |
| PB16 | 8.01 | 147.0653 | [M+H]+ | C_6_H_10_O_4_ | Methylglutaric acid | 12284 | HMDB0000752 | ↑ | 0.024 | 1.89 |
| PB17 | 8.66 | 348.1025 | [M-H]- | C_16_H_19_N_3_O_4_S | Ampicillin | 6249 | HMDB0014559 | ↓ | 0.036 | 0.58 |
| PB18 | 8.85 | 307.1652 | [2M+H]+ | C_8_H_11_NO_2_ | p-Octopamine | 440266 | HMDB0004825 | ↓ | 0.010 | 0.75 |
| PB19 | 8.86 | 263.1126 | [M-H]- | C_13_H_16_N_2_O_4_ | Phenylacetylglutamine | 92258 | HMDB0006344 | ↓ | 0.029 | 0.47 |
| PB20 | 8.92 | 245.094 | [M-H]- | C_13_H_14_N_2_O_3_ | N-acetyl tryptophan | 700653 | HMDB0013713 | ↓ | 0.015 | 0.73 |
| PB21 | 9.11 | 274.1185 | [M+H]+ | C_14_H_15_N_3_O_3_ | Metazachlor | 49384 | HMDB0254508 | ↓ | 0.020 | 0.44 |
| PB22 | 9.35 | 348.0693 | [M+H]+ | C_10_H_14_N_5_O_7_P | dGMP | 65059 | HMDB0001044 | ↓ | 0.034 | 0.65 |
| PB23 | 9.35 | 364.0431 | [M+Na]+ | C_17_H_11_NO_7_ | Aristolochic acid | 2236 | HMDB0248598 | ↓ | 0.036 | 0.69 |
| PB24 | 10.87 | 501.1986 | [2M-H]- | C_9_H_17_NO_7_ | Muramic acid | 22833605 | HMDB0003254 | ↑ | 0.032 | 3.37 |
| PB25 | 11.08 | 190.1186 | [M+H]+ | C_8_H_15_NO_4_ | Castanospermine | 2592 | HMDB0249700 | ↑ | 0.049 | 2.01 |
| PB26 | 15.70 | 166.0298 | [M-H]- | C_3_H_9_N_3_O_3_S | Taurocyamine | 46926103 | HMDB0003584 | ↑ | 0.033 | 1.23 |
| PB27 | 16.63 | 342.1693 | [M+H]+ | C_20_H_23_NO_4_ | Naltrexone | 5360515 | HMDB0014842 | ↑ | 0.035 | 2.41 |
| PB28 | 18.92 | 459.0927 | [M-H]- | C_22_H_20_O_11_ | Wogonoside | 12004622 | HMDB0259901 | ↑ | 0.000 | 9.48 |
| PB29 | 19.19 | 639.1951 | [M-H]- | C_29_H_36_O_16_ | Plantamajoside | 4484595 | HMDB0256632 | ↑ | 0.000 | 38.19 |
| PB30 | 19.24 | 457.1189 | [M-H]- | C_21_H_22_N_4_O_6_S | Raltitrexed | 104758 | HMDB0014438 | ↑ | 0.021 | 2.20 |
| PB31 | 20.20 | 239.1389 | [M+H]+ | C_12_H_18_N_2_O_3_ | Secobarbital | 5193 | HMDB0014562 | ↑ | 0.036 | 1.92 |
| PB32 | 20.20 | 267.1343 | [M+H]+ | C_13_H_18_N_2_O_4_ | Thr-Phe | 7010579 | HMDB0029068 | ↑ | 0.031 | 2.01 |
| PB33 | 20.20 | 249.1215 | [M+Na]+ | C_11_H_18_N_2_O_3_ | Pentobarbital | 4737 | HMDB0014457 | ↑ | 0.016 | 1.85 |
| PB34 | 21.07 | 321.0402 | [M-H]- | C_11_H_12_Cl_2_N_2_O_5_ | 5-Thymidylic acid | 9700 | HMDB0001227 | ↑ | 0.043 | 3.46 |
| PB35 | 21.70 | 365.0334 | [M+Na]+ | C_12_H_14_N_4_O_4_S_2_ | Thiophanate-methyl | 3032791 | HMDB0259025 | ↑ | 0.000 | 51.79 |
| PB36 | 21.71 | 285.0756 | [M+H]+ | C_16_H_12_O_5_ | Biochanin A | 5280373 | HMDB0002338 | ↑ | 0.000 | 28.04 |
| PB37 | 21.98 | 361.1389 | [M+H]+ | C_18_H_20_N_2_O_6_ | Nitrendipine | 4507 | HMDB0015187 | ↑ | 0.001 | 1.37 |
| PB38 | 22.06 | 286.2015 | [M+NH_4_]+ | C_15_H_24_O_4_ | Acoric acid | 25750964 | HMDB0038165 | ↓ | 0.024 | 0.62 |
| PB39 | 22.96 | 496.1782 | [M+NH_4_]+ | C_23_H_26_O_11_ | Isolindleyin | 5273567 | HMDB0039378 | ↑ | 0.000 | 51.78 |
| PB40 | 23.20 | 476.1909 | [M+H]+ | C_24_H_29_NO_9_ | Codeine-6-glucuronide | 5489029 | HMDB0060464 | ↑ | 0.008 | 1.90 |
| PB41 | 24.52 | 380.1146 | [M+H]+ | C_13_H_21_N_3_O_8_S | Lactoylglutathione | 440018 | HMDB0001066 | ↑ | 0.030 | 2.01 |

## Table S8 The list of 104 immunomodulation PBs of AROL in feces.

| No. | RT  (min) | *m/z*(Da) | Ion Type | Formula | Identity | Pubchem CID | HMDB ID | Trend | *p*-value | FC  d15/d0 |
| --- | --- | --- | --- | --- | --- | --- | --- | --- | --- | --- |
| **Lipidomics analysis** | | | | | | | | | | |
| PB1 | 0.57 | 178.0549 | [M-H]- | C_6_H_13_NO_3_S | Cyclamic acid | 46781088 | HMDB0031340 | ↓ | 0.034 | 0.36 |
| PB2 | 0.63 | 121.0672 | [M-H]- | C_8_H_10_O | 3-Ethylphenol | 12101 | HMDB0059873 | ↓ | 0.015 | 0.20 |
| PB3 | 0.66 | 207.0663 | [M-H]- | C_11_H_12_O_4_ | (R)-2-Benzylsuccinate | 3858 | HMDB0012127 | ↓ | 0.029 | 0.40 |
| PB4 | 0.66 | 164.0728 | [M-H]- | C_9_H_11_NO_2_ | L-Phenylalanine | 994 | HMDB0000159 | ↓ | 0.040 | 0.68 |
| PB5 | 0.66 | 151.0371 | [M-H]- | C_8_H_8_O_3_ | 4-Hydroxy-3-methylbenzoic acid | 68138 | HMDB0004815 | ↓ | 0.033 | 0.42 |
| PB6 | 0.69 | 149.0613 | [M-H]- | C_9_H_10_O_2_ | 2-Phenylpropionate | 12073 | HMDB0011743 | ↓ | 0.016 | 0.53 |
| PB7 | 0.75 | 405.2661 | [M-H]- | C_24_H_38_O_5_ | 3-Oxocholic acid | 5283956 | HMDB0000502 | ↓ | 0.029 | 0.39 |
| PB8 | 0.76 | 452.274 | [M-H]- | C_21_H_44_NO_7_P | LysoPE (0:0/16:0) | 53480922 | HMDB0011503 | ↓ | 0.018 | 0.33 |
| PB9 | 0.81 | 267.0656 | [M-H]- | C_16_H_12_O_4_ | Formononetin | 5280378 | HMDB0005808 | ↑ | 0.000 | 12.80 |
| PB10 | 0.82 | 269.0813 | [M+H]+ | C_16_H_12_O_4_ | Dalbergin | 129641556 | HMDB0303925 | ↑ | 0.000 | 12.02 |
| PB11 | 0.90 | 366.3081 | [M+H]+ | C_22_H_39_NO_3_ | N-Linoleoyl GABA | 6438152 | HMDB0062334 | ↓ | 0.020 | 0.51 |
| PB12 | 0.91 | 323.1739 | [M-H]- | C_18_H_28_O_5_ | Hymeglusin | 6440895 | HMDB0341330 | ↓ | 0.035 | 0.67 |
| PB13 | 0.98 | 436.2768 | [M-H]- | C_21_H_44_NO_6_P | LysoPE (P-16:0/0:0) | 23425815 | HMDB0011152 | ↓ | 0.029 | 0.54 |
| PB14 | 0.98 | 389.2699 | [M-H]- | C_24_H_38_O_4_ | 12-Ketodeoxycholic acid | 3080612 | HMDB0000328 | ↓ | 0.035 | 0.55 |
| PB15 | 0.99 | 381.309 | [M+NH_4_]+ | C_22_H_37_NO_3_ | 20-HETE ethanolamide | 35027640 | HMDB0013630 | ↓ | 0.042 | 0.46 |
| PB16 | 1.03 | 583.2551 | [M+H]+ | C_33_H_34_N_4_O_6_ | Biliverdin | 5353439 | HMDB0001008 | ↓ | 0.033 | 0.43 |
| PB17 | 1.03 | 391.2835 | [M+Na]+ | C_24_H_38_O_4_ | Dioctyl phthalate | 8346 | HMDB0251427 | ↓ | 0.009 | 0.54 |
| PB18 | 1.05 | 407.325 | [M+Na]+ | C_27_H_44_O | Cholestenone | 91477 | HMDB0000921 | ↓ | 0.047 | 0.51 |
| PB19 | 1.10 | 403.2937 | [M+NH_4_]+ | C_24_H_35_NO_3_ | N-Docosahexaenoyl Glycine | 53393963 | HMDB0242014 | ↓ | 0.022 | 0.52 |
| PB20 | 1.13 | 424.247 | [M-H]- | C_19_H_40_NO_7_P | LysoPE (14:0/0:0) | 9547070 | HMDB0011500 | ↓ | 0.037 | 0.60 |
| PB21 | 1.84 | 440.2784 | [M+H]+ | C_20_H_42_NO_7_P | LysoPE (0:0/15:0) | 53480921 | HMDB0011472 | ↓ | 0.033 | 0.54 |
| PB22 | 2.22 | 496.3389 | [M+H]+ | C_24_H_50_NO_7_P | LysoPC (16:0) | 460602 | HMDB0010382 | ↓ | 0.004 | 0.41 |
| PB23 | 3.20 | 360.2671 | [M+Cl]- | C_20_H_39_NO_2_ | Oleoylethanolamide | 5283454 | HMDB0002088 | ↓ | 0.043 | 0.58 |
| PB24 | 3.26 | 482.3245 | [M+H]+ | C_23_H_48_NO_7_P | LysoPE (18:0) | 46891690 | HMDB0011130 | ↓ | 0.045 | 0.55 |
| PB25 | 3.32 | 563.2651 | [M+H]+ | C_34_H_34_N_4_O_4_ | protoporphyrin | 387115842 | HMDB0000241 | ↓ | 0.010 | 0.45 |
| PB26 | 3.48 | 466.3283 | [M+H]+ | C_23_H_48_NO_6_P | LysoPE (P-18:0/0:0) | 42607470 | HMDB0240598 | ↓ | 0.029 | 0.51 |
| PB27 | 4.81 | 585.4106 | [M+Na]+ | C_34_H_58_O_6_ | Campesterol glucoside | 12895785 | HMDB0303802 | ↓ | 0.030 | 0.61 |
| PB28 | 4.82 | 401.3875 | [M+H]+ | C_28_H_48_O | campesterol | 5283637 | HMDB0002869 | ↓ | 0.029 | 0.49 |
| PB29 | 5.09 | 415.3931 | [M+H]+ | C_29_H_50_O | 4,4-Dimethyl-5a-cholesta-8-en-3b-ol | 23724604 | HMDB0006840 | ↓ | 0.006 | 0.49 |
| PB30 | 5.09 | 594.4718 | [M+NH_4_]+ | C_35_H_60_O_6_ | DG (12:0/0:0/20:3(5Z,8Z,11Z)-O(14R,15S)) | 157002714 | HMDB0294607 | ↓ | 0.003 | 0.62 |
| PB31 | 5.72 | 714.5515 | [M+H]+ | C_40_H_75_NO_9_ | Soyacerebroside I | 131751281 | HMDB0032677 | ↓ | 0.010 | 0.54 |
| PB32 | 5.81 | 747.5629 | [M+HCOO]- | C_39_H_79_N_2_O_6_P | SM (d18:1/16:0) | 164199100 | HMDB0010169 | ↓ | 0.009 | 0.46 |
| PB33 | 6.02 | 703.5731 | [M+H]+ | C_39_H_79_N_2_O_6_P | SM (d18:0/16:1(9Z)) | 53481781 | HMDB0013464 | ↓ | 0.017 | 0.36 |
| PB34 | 6.05 | 862.6254 | [M+H]+ | C_46_H_87_NO_13_ | Galabiosylceramide (d18:1/16:0) | 20057274 | HMDB0004833 | ↓ | 0.005 | 0.45 |
| PB35 | 6.10 | 744.5634 | [M+HCOO]- | C_40_H_77_NO_8_ | GlcCer (d18:1/16:0) | 45039325 | HMDB0004971 | ↓ | 0.009 | 0.48 |
| PB36 | 6.12 | 738.5496 | [M+Na]+ | C_40_H_78_NO_7_P | PC (P-16:0/16:1(9Z)) | 52923882 | HMDB0011207 | ↓ | 0.042 | 0.51 |
| PB37 | 6.13 | 698.5549 | [M+H]+ | C_40_H_75_NO_8_ | N-[(4E,8Z)-1,3-dihydroxyoctadeca-4,8-dien-2-yl]hexadecanamide 1-glucoside | 14522366 | HMDB0034623 | ↓ | 0.006 | 0.51 |
| PB38 | 6.13 | 554.5045 | [M+H]+ | C_34_H_67_NO_4_ | Heptacosanoylcarnitine | 156962556 | HMDB0241643 | ↓ | 0.017 | 0.60 |
| PB39 | 6.17 | 702.5057 | [M+H]+ | C_38_H_72_NO_8_P | PC (14:1(9Z)/16:1(9Z)) | 52922252 | HMDB0007903 | ↓ | 0.012 | 0.46 |
| PB40 | 6.28 | 705.5906 | [M+H]+ | C_39_H_81_N_2_O_6_P | SM (d18:0/16:0) | 5283591 | HMDB0010168 | ↓ | 0.014 | 0.47 |
| PB41 | 6.43 | 718.5844 | [M+H]+ | C_40_H_80_NO_7_P | PC (P-18:0/14:0) | 52923926 | HMDB0011236 | ↓ | 0.012 | 0.61 |
| PB42 | 6.51 | 413.3648 | [M+H]+ | C_29_H_48_O | Stigmasterol | 5280794 | HMDB0000937 | ↓ | 0.010 | 0.69 |
| PB43 | 6.79 | 731.6031 | [M+H]+ | C_41_H_83_N_2_O_6_P | SM (d18:0/18:1(9Z)) | 44260139 | HMDB0012089 | ↓ | 0.013 | 0.32 |
| PB44 | 6.82 | 734.5666 | [M+H]+ | C_40_H_80_NO_8_P | PC (16:0/16:0) | 452110 | HMDB0000564 | ↓ | 0.030 | 0.43 |
| PB45 | 6.90 | 760.585 | [M+H]+ | C_42_H_82_NO_8_P | PC (14:1(9Z)/20:0) | 52922276 | HMDB0007911 | ↓ | 0.029 | 0.46 |
| PB46 | 7.45 | 772.6224 | [M+H]+ | C_44_H_86_NO_7_P | PC (P-18:0/18:1(9Z)) | 42607428 | HMDB0011243 | ↓ | 0.039 | 0.36 |
| PB47 | 7.63 | 759.5636 | [M+CH_3_COO]- | C_39_H_77_N_2_O_6_P | SM (d18:1/16:1(9Z)) | 52931235 | HMDB0240613 | ↓ | 0.007 | 0.70 |
| PB48 | 7.66 | 838.6309 | [M+H]+ | C_48_H_88_NO_8_P | PC 40:4 | 53479183 | HMDB0008567 | ↓ | 0.027 | 0.47 |
| PB49 | 8.25 | 854.6739 | [M+HCOO]- | C_48_H_91_NO_8_ | GlcCer (d18:1/24:1(15Z)) | 126503397 | HMDB0004975 | ↓ | 0.021 | 0.55 |
| PB50 | 8.29 | 828.6549 | [M+HCOO]- | C_46_H_89_NO_8_ | GlcCer (d18:1/22:0) | 10373084 | HMDB0004974 | ↓ | 0.039 | 0.56 |
| PB51 | 8.36 | 846.6664 | [M+Cl]- | C_48_H_93_NO_8_ | GlcCer (d18:1/24:0) | 6321361 | HMDB0004978 | ↓ | 0.003 | 0.60 |
| PB52 | 8.89 | 799.6851 | [M+Na]+ | C_49_H_92_O_6_ | TG (14:0/18:1(11Z)/14:0) | 131753473 | HMDB0042331 | ↓ | 0.022 | 0.41 |
| PB53 | 9.05 | 850.67 | [M+H]+ | C_50_H_92_NO_7_P | PC (O-22:1(13Z)/20:4(8Z,11Z,14Z,17Z)) | 53481757 | HMDB0013451 | ↓ | 0.013 | 0.59 |
| PB54 | 9.21 | 834.6798 | [M+Na]+ | C_48_H_93_NO_8_ | GlcCer (d18:1/24:0) | 6321361 | HMDB0004978 | ↓ | 0.027 | 0.49 |
| PB55 | 9.30 | 852.6874 | [M+NH_4_]+ | C_57_H_86_O_4_ | 3,4-dihydroxy-5-all-trans-decaprenylbenzoate | 25202855 | HMDB0062204 | ↓ | 0.025 | 0.59 |
| PB56 | 9.55 | 856.7004 | [M+NH_4_]+ | C_53_H_90_O_7_ | Sitosterol 3-O-(6'-O-linoleyl-beta-D-glucoside) | 157009924 | HMDB0302624 | ↓ | 0.024 | 0.46 |
| PB57 | 9.55 | 857.7029 | [M+K]+ | C_52_H_98_O_6_ | TG (15:0/18:1(11Z)/16:0) | 131754349 | HMDB0043222 | ↓ | 0.036 | 0.44 |
| PB58 | 9.74 | 885.6841 | [M+CH_3_COO]- | C_52_H_90_O_7_ | Campesterol 6'-(9Z-octadecenoyl)-glucoside | 70699342 | HMDB0036292 | ↓ | 0.024 | 0.60 |
| PB59 | 9.77 | 818.6865 | [M+NH_4_]+ | C_50_H_88_O_7_ | Campesterol 6'-hexadecanoylglucoside | 13051625 | HMDB0036291 | ↓ | 0.013 | 0.54 |
| PB60 | 9.95 | 699.5874 | [M+Na]+ | C_43_H_80_O_5_ | DG (22:2(13Z,16Z)/18:0/0:0) | 53478411 | HMDB0007651 | ↓ | 0.012 | 0.53 |
| PB61 | 9.98 | 832.7028 | [M+NH_4_]+ | C_51_H_90_O_7_ | Sitoindoside I | 13051622 | HMDB0030836 | ↓ | 0.005 | 0.53 |
| PB62 | 10.01 | 859.7227 | [M+K]+ | C_52_H_100_O_6_ | TG (14:0/20:0/15:0) | 131753324 | HMDB0042182 | ↓ | 0.018 | 0.41 |
| PB63 | 10.02 | 858.718 | [M+NH_4_]+ | C_53_H_92_O_7_ | Sitoindoside II | 131751526 | HMDB0034089 | ↓ | 0.030 | 0.45 |
| PB64 | 11.57 | 867.6818 | [M+K]+ | C_53_H_96_O_6_ | TG 50:3 | 25240357 | HMDB0005433 | ↓ | 0.035 | 0.64 |
| **Metabolomics analysis** | | | | | | | | | | |
| PB1 | 0.93 | 426.9675 | [2M-H]- | C_4_H_7_O_8_P | 2-Oxo-3-hydroxy-4-phosphobutanoic acid | 119688 | HMDB0006801 | ↑ | 0.000 | 60.42 |
| PB2 | 0.93 | 869.5614 | [M-H]- | C_47_H_82_O_12_S | PG (PGJ2/i-21:0) | 156975368 | HMDB0272016 | ↑ | 0.045 | 2.56 |
| PB3 | 0.96 | 601.4246 | [M-H]- | C_40_H_58_O_4_ | Karpoxanthin | 5282164 | HMDB0034977 | ↑ | 0.027 | 4.17 |
| PB4 | 0.96 | 615.4419 | [M-H]- | C_41_H_60_O_4_ | Oryzanol B | 9920169 | HMDB0302834 | ↑ | 0.025 | 3.25 |
| PB5 | 0.98 | 563.5039 | [M-H]- | C_36_H_68_O_4_ | 2-OAHSA | 134778484 | HMDB0112140 | ↑ | 0.025 | 2.84 |
| PB6 | 0.99 | 365.3403 | [M-H]- | C_24_H_46_O_2_ | Nervonic acid | 5281120 | HMDB0002368 | ↑ | 0.027 | 1.83 |
| PB7 | 0.99 | 685.5417 | [M-H]- | C_43_H_74_O_6_ | DG (20:5(5Z,8Z,11Z,14Z,16E)-OH(18R)/0:0/20:0) | 157004461 | HMDB0296354 | ↑ | 0.022 | 2.34 |
| PB8 | 1.01 | 616.4973 | [M-H]- | C_35_H_71_NO_5_S | Cer (t18:0/20:5(6E,8Z,11Z,14Z,17Z)-OH(5)) | 156997779 | HMDB0290182 | ↑ | 0.043 | 1.77 |
| PB9 | 1.02 | 281.2487 | [M-H]- | C_18_H_34_O_2_ | Oleic acid | 445639 | HMDB0000207 | ↑ | 0.034 | 2.17 |
| PB10 | 1.03 | 253.2189 | [M-H]- | C_16_H_30_O_2_ | Palmitoleic acid | 445638 | HMDB0003229 | ↑ | 0.016 | 1.65 |
| PB11 | 1.05 | 950.7087 | [M-H]- | C_58_H_97_NO_9_ | PE-NMe2 (24:1(15Z)/22:4(7Z,10Z,13Z,16Z)) | 131821761 | HMDB0114654 | ↑ | 0.038 | 3.11 |
| PB12 | 1.10 | 573.4515 | [M-H]- | C_36_H_62_O_5_ | FA 40:9 | 131801731 | HMDB0056004 | ↑ | 0.033 | 1.94 |
| PB13 | 1.28 | 537.4389 | [M+H]+ | C_40_H_56_ | B-Carotene | 5280489 | HMDB0000561 | ↑ | 0.011 | 3.14 |
| PB14 | 1.28 | 536.437 | [M+NH_4_]+ | C_32_H_54_O_5_ | Ganoderiol C | 15602259 | HMDB0037781 | ↑ | 0.012 | 2.88 |
| PB15 | 1.37 | 204.124 | [M-H]- | C_9_H_19_NO_4_ | Pantothenol | 131204 | HMDB0004231 | ↓ | 0.035 | 0.66 |
| PB16 | 2.01 | 277.2164 | [M+H]+ | C_18_H_28_O_2_ | 19-Norandrosterone | 6426906 | HMDB0002697 | ↑ | 0.047 | 1.49 |
| PB17 | 2.01 | 505.3514 | [M+H]+ | C_30_H_48_O_6_ | Cyclopassifloic acid D | 45359819 | HMDB0035948 | ↑ | 0.032 | 1.59 |
| PB18 | 2.01 | 489.3484 | [M+H]+ | C_30_H_48_O_5_ | Rotundic acid | 119034 | HMDB0257316 | ↑ | 0.024 | 1.69 |
| PB19 | 2.02 | 351.2146 | [M+H]+ | C_20_H_30_O_5_ | PGH3 | 45783059 | HMDB0013040 | ↑ | 0.022 | 1.25 |
| PB20 | 2.02 | 311.2214 | [M+H]+ | C_18_H_30_O_4_ | 12(13) Ep-9-KODE | 5283007 | HMDB0013623 | ↑ | 0.048 | 1.42 |
| PB21 | 2.03 | 481.2808 | [M+H]+ | C_26_H_40_O_8_ | Neoandrographolide | 9848024 | HMDB0255516 | ↑ | 0.034 | 1.42 |
| PB22 | 2.85 | 539.0864 | [2M-H]- | C_15_H_10_O_5_ | Genistein | 5280961 | HMDB0003217 | ↓ | 0.027 | 0.20 |
| PB23 | 3.77 | 220.1177 | [M+NH_4_]+ | C_9_H_14_O_5_ | 4-Heptenal diethyl acetal | 10998061 | HMDB0032306 | ↓ | 0.007 | 0.43 |
| PB24 | 4.35 | 225.0945 | [M+H]+ | C_10_H_12_N_2_O_4_ | Hydroxykynurenine | 25244175 | HMDB0000732 | ↑ | 0.008 | 3.13 |
| PB25 | 4.49 | 207.0686 | [M-H]- | C_11_H_12_O_4_ | 5-(3',4'-Dihydroxyphenyl)-gamma-valerolactone | 444797 | HMDB0029185 | ↓ | 0.021 | 0.45 |
| PB26 | 4.57 | 487.1963 | [M-H]- | C_26_H_32_O_9_ | Terretonin | 16196970 | HMDB0035860 | ↓ | 0.025 | 0.32 |
| PB27 | 4.58 | 731.2682 | [M+HCOO]- | C_32_H_46_O_16_ | Secoisolariciresinol 9,9'-diglucoside | 9917980 | HMDB0036323 | ↓ | 0.043 | 0.40 |
| PB28 | 4.98 | 380.1446 | [M-H]- | C_16_H_23_N_5_O_4_S | 2-Methylthio-N6-(delta2-isopentenyl) adenosine | 53297381 | HMDB0301782 | ↑ | 0.026 | 1.41 |
| PB29 | 5.00 | 149.1079 | [M+H]+ | C_9_H_12_N_2_ | Nornicotine | 412 | HMDB0001126 | ↓ | 0.028 | 0.58 |
| PB30 | 5.65 | 224.0795 | [M+CH_3_COO]- | C_6_H_7_N_5_O | N2-Methylguanine | 16212222 | HMDB0006040 | ↓ | 0.020 | 0.55 |
| PB31 | 6.42 | 248.0249 | [M-H]- | C_9_H_13_ClNO_3_P | Phaclofen | 1641 | HMDB0256382 | ↓ | 0.032 | 0.52 |
| PB32 | 6.62 | 214.0734 | [M-H]- | C_10_H_9_N_5_O | Kinetin | 3830 | HMDB0012245 | ↑ | 0.040 | 1.71 |
| PB33 | 6.70 | 173.0816 | [M-H]- | C_8_H_14_O_4_ | Suberic acid | 10457 | HMDB0000893 | ↓ | 0.028 | 0.51 |
| PB34 | 7.22 | 89.0265 | [M-H]- | C_3_H_6_O_3_ | Glyceraldehyde | 751 | HMDB0001051 | ↓ | 0.043 | 0.76 |
| PB35 | 8.16 | 87.0473 | [M-H]- | C_4_H_8_O_2_ | Isobutyric acid | 6590 | HMDB0001873 | ↓ | 0.048 | 0.52 |
| PB36 | 8.58 | 182.9971 | [M-H]- | C_5_H_4_N_4_O_2_S | 6-Thiourate | 3032417 | HMDB0060417 | ↓ | 0.009 | 0.36 |
| PB37 | 8.62 | 226.0163 | [M-H]- | C_7_H_5_N_3_O_6_ | Trinitrotoluene | 8376 | HMDB0245483 | ↓ | 0.015 | 0.33 |
| PB38 | 9.25 | 253.0563 | [M-H]- | C_15_H_10_O_4_ | Daidzein | 5281708 | HMDB0003312 | ↓ | 0.045 | 0.60 |
| PB39 | 9.37 | 303.1301 | [M+H]+ | C_15_H_10_O_7_ | 2-(2,6-Dihydroxyphenyl)-3,5,7-trihydroxychromen-4-one | 5320471 | HMDB0041689 | ↑ | 0.000 | 7.46 |
| PB40 | 10.03 | 342.1393 | [M+H]+ | C_20_H_23_NO_4_ | Isocorydine | 10143 | HMDB0030184 | ↓ | 0.029 | 0.42 |
